# Peer Support Intervention for Suicide Prevention Among High-Risk Adults in Michigan: A Randomized Clinical Trial

**DOI:** 10.1001/jamanetworkopen.2025.10808

**Published:** 2025-05-28

**Authors:** Paul N. Pfeiffer, Kristen M. Abraham, Adrienne Lapidos, Eduardo Vega, Jennifer Jagusch, James Garlick, Sara Pasiak, Dara Ganoczy, H. Myra Kim, Brian Ahmedani, Mark Ilgen, Cheryl King

**Affiliations:** 1 Department of Psychiatry, University of Michigan Medical School, Ann Arbor; 2 VA Center for Clinical Management Research, Ann Arbor, Michigan; 3 Department of Psychology, University of Detroit Mercy, Detroit, Michigan; 4 Palliance Institute for Peer Support and Lived Expertise, Los Angeles, California; 5 TRAILS, Ann Arbor, Michigan; 6 Consulting for Statistics, Computing, and Analytics Research, University of Michigan, Ann Arbor; 7 Henry Ford Health, Center for Health Policy and Health Services Research, Detroit, Michigan

## Abstract

**Importance:**

Innovative approaches to address interpersonal risk factors for suicide attempts and suicidal ideation may help reduce persistently elevated suicide rates in the US.

**Objective:**

To determine whether Peers for Valued Living (PREVAIL), a posthospital peer support intervention, reduces suicide attempts and suicidal ideation among adults psychiatrically hospitalized for suicide risk.

**Design, Setting, and Participants:**

This single-masked, randomized clinical trial recruited adult psychiatric inpatients aged 18 years or older from 3 Michigan-based facilities between June 22, 2018, and December 30, 2022. The study compared 3 months of the PREVAIL intervention plus enhanced usual care with enhanced usual care only, with 3 months of postintervention follow-up.

**Intervention:**

One-on-one support from a peer specialist initiated during hospital admission and continued 3 months after discharge.

**Main Outcomes and Measures:**

Suicide attempts were measured using the Columbia Suicide Severity Rating Scale, and suicidal ideation was measured using the Beck Scale for Suicidal Ideation at 3 and 6 months after randomization.

**Results:**

Of 5310 patients screened, 455 were randomized using a minimization algorithm. A total of 229 participants were randomized to the PREVAIL peer mentorship arm (mean [SD] age, 32.4 [14.0] years, 134 women [58.5%]), and 226 were randomized to the enhanced usual care arm (mean [SD] age, 31.6 [13.5] years; 139 women [61.5%]). In the intention-to-treat sample with nonmissing data, the percentage of participants with any suicide attempt over 6 months (including participants who only completed 3-month follow-up assessments) was 17.2% (28 of 163) for enhanced usual care and 14.9% (24 of 161 with 2 deaths by suicide) for PREVAIL. Mean (SD) suicidal ideation scores were 4.3 (95% CI, 3.2-5.3) for enhanced usual care and 4.9 (95% CI, 3.7-6.1) for PREVAIL at 6 months. There were no statistically significant effects of the intervention for either outcome in the primary analyses. In post hoc analyses, the interaction between study arm and the COVID-19 pandemic was statistically significant. The enhanced usual care arm had a mean (SD) 6-month suicidal ideation score of 3.5 (6.1) before and 6.1 (7.4) after the pandemic vs the PREVAIL arm (mean [SD] scores, 5.0 [7.7] and 4.5 [6.6], respectively). There were 2 study-related adverse events, with 1 resulting in participant withdrawal from the intervention arm.

**Conclusions and Relevance:**

In this randomized clinical trial, the peer-delivered PREVAIL intervention up to 3 months after discharge did not significantly differ from enhanced usual care in terms of reducing subsequent suicide attempts or suicidal ideation.

**Trial Registration:**

ClinicalTrials.gov Identifier: NCT03373916

## Key Points

**Question:**

Does 3 months of an individual peer support intervention reduce suicide attempts or suicidal ideation among adults following psychiatric hospitalization for suicide risk?

**Findings:**

In this randomized clinical trial of 455 participants, suicide attempt and suicidal ideation outcomes at 6 months did not differ significantly between the peer support and enhanced usual care control groups. The percentage of suicide attempts at 6 months after randomization was 14.9% for peer support recipients (including 2 deaths by suicide) vs 17.2% for control recipients (no deaths by suicide).

**Meaning:**

These findings suggest that the addition of a peer support intervention is not more effective than enhanced usual care alone for reducing suicide attempts or suicidal ideation.

## Teaser

This randomized clinical trial investigates whether a posthospital peer support intervention reduces suicide attempts and suicidal ideation among adults psychiatrically hospitalized for suicide risk.

## Introduction

Persistently elevated suicide rates in the US underscore the need for novel approaches to suicide prevention.^1^ Peer support, including when provided by a Certified Peer Specialist with expertise in using their lived experience with mental health treatment and recovery to help others,^2^ has the potential to reduce interpersonal risk factors for suicide.^3^ Peer support interventions may improve hope by providing role models of recovery from a suicidal crisis or by helping individuals identify and work toward personally meaningful goals.^4,5^ By establishing a shared experience related to mental health and engaging in nonjudgmental discussions, a peer support intervention may also increase belongingness.^6^ Working with a peer to strengthen natural social supports or to take on valued roles in social activities may also increase belongingness while reducing burdensomeness.^7^

Evidence for the effectiveness of peer-led interventions is supported by a small number of clinical trials finding positive effects on hope^8,9,10^; however, subsequent meta-analyses found null associations with hope, hopelessness, social support, and social functioning.^11,12,13,14,15^ These meta-analyses did not include studies designed specifically for suicide prevention, and prior studies have not examined suicidal ideation or suicide attempts as outcomes.^16^ Peers for Valued Living (PREVAIL), developed specifically to address interpersonal risk factors for suicide through 3 months of 1:1, semistructured peer support by state-certified peer specialists, was previously shown to be feasible and acceptable in a small-scale pilot study.^17^ We now present the findings from a larger randomized clinical trial designed to test the effects of the intervention on suicide attempts and suicidal ideation among adults at high risk for suicide.

## Methods

### Participants

This randomized clinical trial recruited participants from 3 inpatient psychiatry units in southeast Michigan (1 from an academic medical center and 2 from a large private health system), from June 22, 2018, to December 30, 2022. The study received University of Michigan Institutional Review Board approval, and participants provided written informed consent. Participants recruited during and after the COVID-19 pandemic provided verbal informed consent. The full trial protocol was previously published^18^ and is provided in Supplement 1. The study followed the Consolidated Standards of Reporting Trials (CONSORT) reporting guideline.

Patients were screened for the following inclusion criteria: (1) aged at least 18 years, (2) medical record documentation of suicidal ideation or recent suicide attempt, (3) Beck Scale for Suicidal Ideation (BSI) score of at least 5 one week prior to admission,^19^ (4) English speaking, and (5) reliable telephone access. Exclusion criteria were as follows: (1) attending psychiatrist determination that a peer relationship would not be feasible or helpful due to unstable psychosis, cognitive disorder, or severe personality disorder; (2) cognitive impairment according to the Mini-Cog screening test^20^; (3) already receiving or intending to receive peer specialist support for suicide prevention; (4) residing 50 miles or more from any study peer (dropped after the COVID-19 pandemic led to a shift to virtual meetings); (5) planned discharge to another inpatient or residential facility; (6) receipt of electroconvulsive therapy; or (7) homicidal ideation or violent behavior in the past 6 months.

With a planned enrollment of 490 total participants and an estimated 25% dropout rate based on pilot study results,^17^ our projected 184 participants per group in the analytic sample was expected to provide 84% power to detect a difference in a 6-month suicide attempt rate of 22% in the intervention arm vs 36% in the control arm (estimated from a prior suicide prevention trial^21^) using a 2-sided .05-level test. We also expected to detect with 90% power a standardized effect size (Cohen *d*) of 0.34 for suicidal ideation, corresponding to a BSI score of 2.5 in the intervention arm vs 4.0 in the control arm at 6 months. Suicide attempts and suicidal ideation were both considered primary outcomes, and we did not adjust for multiple comparisons.

### Randomization

Following completion of all baseline assessments, we assigned participants to PREVAIL plus enhanced usual care or enhanced usual care alone using a web-based sequential treatment assignment algorithm stratified by recruitment site.^22^ The algorithm used nonuniform assignment probabilities with a randomization component to nondeterministically reduce imbalance on gender and recent suicide attempt.

### PREVAIL Intervention

PREVAIL consisted of 3 months of 1:1 peer support from a State of Michigan Certified Peer Support Specialist or Certified Peer Recovery Coach. Peers typically carried caseloads of 2 to 5 participants at a time. All study peers had at least 1 year of work experience providing peer support, a lived experience of suicidal ideation or suicide attempt, and a history of receiving mental health services. Peers were recruited using email campaigns to individuals who had completed state certification trainings. Training in the PREVAIL intervention consisted of 24 hours of didactic instruction, group discussion, and role play.

Participants selected the peer they worked with after reviewing autobiographical sketches of available peers. Meeting frequency and location were flexible, but weekly meetings in the community (eg, a coffee shop) were most common until the COVID-19 pandemic, after which all meetings occurred via telephone or videoconferencing. Participants were contacted by study staff after their first meeting with the peer and were provided instructions to reach study staff with any concerns related to peer interactions.

Meeting content included discussing the participants’ struggles or successes. Peers also engaged participants in semistructured conversations related to hope, social support, managing distress, and maintaining wellness. Peers asked about suicidal ideation at each meeting and followed a scripted algorithm for positive responses to determine whether to involve an on-call study clinician for further risk management. Fidelity to the PREVAIL intervention was maintained by reviewing participant interactions, training materials, and audio recordings of sessions during weekly group supervision meetings facilitated by clinical psychologists (K.M.A. and A.L.). These meetings also allowed for peers to connect with and support one another.

### Enhanced Usual Care

In addition to their usual care, participants in both arms received a follow-up contact from the study team within 72 hours of their hospital discharge. As part of each follow-up assessment, a study-specific suicide risk screening was administered and local mental health resource information provided (eg, telephone numbers for community mental health clinics) in case of difficulties with postdischarge follow-up plans.

### Measures

Self-reported demographic characteristics were assessed at baseline. Race and ethnicity were assessed in a combined survey item in which participants could check all that apply (American Indian or Alaska Native; Black, Afro-Caribbean, or African American [hereafter referred to as Black]; East Asian or Asian American [hereafter referred to as Asian]; Latino or Hispanic American; Middle Eastern or Arab American; Native Hawaiian or Pacific Islander; non-Hispanic White or European American [hereafter referred to as White]; South Asian or Indian American; or other [self-described]). Race and ethnicity were measured to inform the generalizability of the sample and because suicide risk varies by race and ethnicity. Primary and secondary outcome measures were collected at baseline prior to randomization and at 3 and 6 months after randomization. Staff who administered study assessments were masked to participant study arm.

Primary outcomes were suicidal ideation according to the BSI and suicide attempts according to the Columbia Suicide Severity Rating Scale.^19,23^ The BSI has a total score range from 0 to 38 (higher scores indicating greater risk) and was administered primarily using the past-week time frame and secondarily as the worst point since the prior assessment.^24^ Suicide attempts were defined dichotomously as any actual, aborted, or interrupted attempt according to the Columbia Suicide Severity Rating Scale. Deaths by suicide were considered suicide attempts. A battery of assessments was used to measure secondary outcomes, including hopelessness (Beck Hopelessness Scale)^25^; hope (Hope Scale)^26^; perceived burdensomeness and thwarted belongingness (Interpersonal Needs Questionnaire subscales)^27^; depression (Patient Health Questionnaire-9)^28^; quality of life (Quality of Life Enjoyment and Satisfaction Questionnaire Short Form)^29^; mental and physical health status (12-Item Short Form)^30^; emotional support, instrumental support, friendship, loneliness, and perceived rejection (National Institutes of Health Adult Toolbox Social Relationship Scales [SRS])^31^; relationships with friends, family, and significant others (Multidimensional Scale of Perceived Social Support [MSPSS])^32^; and use of mental health and substance use services (Health Services Inventory).^33^

After the 6-month follow-up measures were completed, study arm assignment was unmasked, and PREVAIL participants completed a semistructured interview regarding their experiences. Qualitative content analysis was applied to contemporaneous interviewer notes to characterize participants’ perceptions of the impact of PREVAIL on their suicidal thoughts and behaviors.^34^ Fidelity to the PREVAIL intervention was assessed by rating a 20% random selection of recordings of all peer-participant meetings with respect to the skill (categorized as adequate or better vs inadequate) with which peers provided general peer support (ie, listening, sharing, validation), semistructured conversations, and adherence to suicide risk assessment protocols.

### Statistical Analysis

We examined differences between study arms in an a priori–specified set of potentially confounding baseline participant characteristics, and variables that showed significant differences were included as covariates in subsequent analytic models. We also included in analytic models any baseline characteristics that had statistically significant associations with completion of any follow-up assessment.

Primary intention-to-treat analyses included all randomized participants regardless of participation in the intervention. For the primary outcome of suicide attempts over 6 months, we used a logistic regression model with primary estimators of intervention arm, study site, self-selected gender (man, woman, nonbinary, transgender, gender not listed), and any lifetime prior suicide attempt, adjusting for potentially confounding covariates shown to differ between arms at baseline and baseline estimators of missing follow-up. For past-week suicidal ideation assessed at 6 months, we used a multiple regression model with the same estimators and covariates as the logistic regression model for suicide attempts plus baseline values for BSI suicidal ideation score. With respect to missing data, we imputed 10 sets of complete data based on chained equations (sequential regression multivariable imputation) and obtained pooled estimates based on the multiply imputed data accounting for the uncertainty in the imputations.^35^ For each model, the intervention arm coefficient allowed testing for the intervention effect. We used generalized linear mixed-effects models to assess primary outcomes over time (eMethods in Supplement 2).

Secondary per-protocol analyses compared participants assigned to PREVAIL who completed 6 or more meetings with the peer (engagers) with those assigned to enhanced usual care. Post hoc analyses assessed the potential impact of the COVID-19 pandemic and associated change to all virtual peer encounters by including an indicator for participants enrolled during the pandemic (vs before) and an interaction term between that indicator and the intervention arm in the primary analytic models. All analyses were conducted using SAS, version 9.4 (SAS Institute Inc).

## Results

### Participants

**Figure 1.**
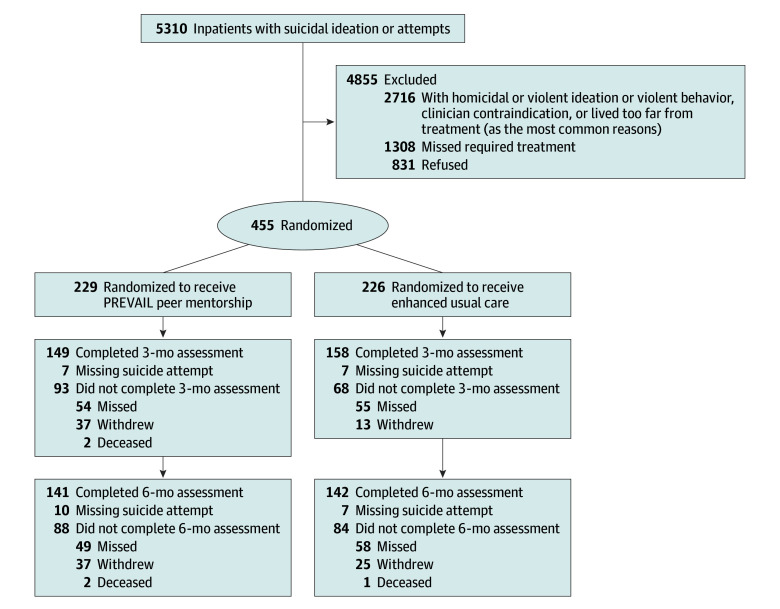
Consolidation Standards of Reporting Trials Diagram

Among 5310 screened patients who had suicidal ideation or a recent suicide attempt at admission to a psychiatric inpatient unit, 455 were enrolled and randomized to 1 of the study arms (Figure 1). A total of 229 participants were randomized to the PREVAIL intervention (mean [SD] age, 32.4 [14.0] years; 78 men [34.1%], 134 women [58.5%], and 17 nonbinary, transgender, or a gender not listed [7.4%]; and 6 of American Indian or Alaska Native [2.6%], 9 Asian [4.0%], 45 Black [19.7%], 8 Latino or Hispanic American [3.5%], 4 South Asian or Indian American [1.8%], 172 White [75.4%], and 4 other [1.8%] race and ethnicity). A total of 226 were randomized to enhanced usual care (mean [SD] age, 31.6 [13.5] years; 76 men [33.6%], 139 women [61.5%], and 11 nonbinary, transgender, or a gender not listed [4.9%]; and 7 of American Indian or Alaska Native [3.1%], 7 Asian [3.1%], 46 Black [20.4%], 15 Latino or Hispanic American [6.6%], 6 Middle Eastern or Arab American [2.7%], 4 South Asian or Indian American [1.8%], 161 White [71.2%], and 4 other [1.8%] race and ethnicity) (Table 1; eTable 1 in Supplement 2). At baseline, PREVAIL participants had a lower mean score on the Beck Hopelessness Scale compared with those receiving enhanced usual care (mean [SD], 8.4 [5.9] vs 9.8 [6.1]) and had greater mean family support on the MSPSS family subscale (mean [SD], 4.4 [2.0] vs 4.1 [1.8]) (eTable 2 in Supplement 2).

**Table 1.** Sample Characteristics

Characteristic	Participants, No. (%)	
	PREVAIL peer mentorship (n = 229)	Enhanced usual care (n = 226)
Gender		
Man	78 (34.1)	76 (33.6)
Woman	134 (58.5)	139 (61.5)
Nonbinary, transgender, gender not listed	17 (7.4)	11 (4.9)
Race and ethnicity^a^		
American Indian or Alaska Native	6 (2.6)	7 (3.1)
Black, Afro-Caribbean, or African American	45 (19.7)	46 (20.4)
East Asian or Asian American	9 (4.0)	7 (3.1)
Latino or Hispanic American	8 (3.5)	15 (6.6)
Middle Eastern or Arab American	0 (0)	6 (2.7)
Native Hawaiian or Pacific Islander	2 (0.9)	0 (0)
Non-Hispanic White or European American	172 (75.4)	161 (71.2)
South Asian or Indian American	4 (1.8)	4 (1.8)
Other, please describe	4 (1.8)	4 (1.8)
Age, mean (SD), y	32.4 (14.0)	31.6 (13.5)
Occupation		
Homemaker/caregiver	11 (4.9)	15 (6.7)
Retired	10 (4.4)	9 (4.0)
Working full or part time	115 (50.7)	123 (54.7)
Volunteer	3 (1.3)	5 (2.2)
Unemployed or on disability	58 (25.6)	57 (25.3)
Student	67 (29.5)	62 (27.6)
Psychiatric discharge diagnoses		
Unipolar depressive disorders	154 (67.5)	155 (68.6)
Anxiety disorders	38 (16.7)	34 (15.0)
Bipolar disorders	25 (11.0)	20 (8.9)
Posttraumatic stress disorder	12 (5.3)	8 (3.5)
Alcohol use disorders	7 (3.1)	9 (4.0)
Other substance use disorders	8 (3.5)	2 (0.9)
Schizophrenia or schizoaffective disorder	2 (0.9)	3 (1.3)
Other psychiatric disorders^b^	77 (33.8)	76 (33.6)
Baseline outcome measures		
Lifetime suicide attempt	197 (86.0)	210 (92.9)
Beck Scale for Suicidal Ideation score, mean (SD)^c^	22.0 (7.9)	22.7 (7.0)
Beck Hopelessness Scale score, mean (SD)^d^	8.4 (5.9)	9.8 (6.1)
Thwarted belongingness score, mean (SD)^e^	22.3 (7.1)	23.0 (6.9)
Perceived burdensomeness score, mean (SD)^f^	16.0 (8.9)	16.2 (8.5)

Abbreviation: PREVAIL, Peers for Valued Living.

^a^ Participants were instructed to check all that apply for the listed race and ethnicity options.

^b^ For example, personality disorders, eating disorders, sleep disorders, attention-deficit/hyperactivity disorder.

^c^ Scored on a scale of 0 to 38, with higher scores indicating greater risk.

^d^ Scored on a scale of 0 to 20, with higher scores indicating more severe hopelessness.

^e^ Scored on a scale of 4 to 28, with higher scores indicating greater thwarted belongingness.

^f^ Scored on a scale of 4 to 28, with higher scores indicating greater perceived burdensomeness.

### Completion of Study Activities and Fidelity

The percentage of all randomized participants who completed follow-up assessments did not differ between arms and was overall 67.5% (307 participants) at 3 months and 62.2% (283 participants) at 6 months. Baseline characteristics associated with not completing follow-up assessments were recruitment site A (odds ratio [OR], 0.56; 95% CI, 0.37-0.84), homelessness (OR, 0.45; 95% CI, 0.23-0.90), and SRS perceived rejection (OR, 0.97; 95% CI, 0.97-0.99), whereas factors associated with follow-up completion were MSPSS friend support (OR, 1.18; 95% CI, 1.06-1.32) and SRS emotional support (OR, 1.18; 95% CI, 1.02-1.07), instrumental support (OR, 1.03; 95% CI, 1.01-1.05), and friendship (OR, 1.03; 95% CI, 1.00-1.05). The 229 participants assigned to the PREVAIL arm completed a median (IQR) of 7 (2-11) peer meetings; 180 (78.6%) completed more than 1 meeting; and 133 (58.1%) were engagers, meaning that they completed 6 or more meetings. The modal days between meetings was 7, and the mean (SD) was 8.3 (4.5) days. eTable 3 in Supplement 2 provides the missing data for each measure by arm and time point. Of 265 sessions rated for fidelity, at least adequate skill was achieved in 256 (96.6%) for listening, 244 (92.1%) for sharing, 259 (97.7%) for empathy, 207 (78.1%) for semistructured conversations, and 223 (84.2%) for suicide risk assessments.

### Suicidal Ideation and Suicide Attempt Outcomes

**Figure 2.**
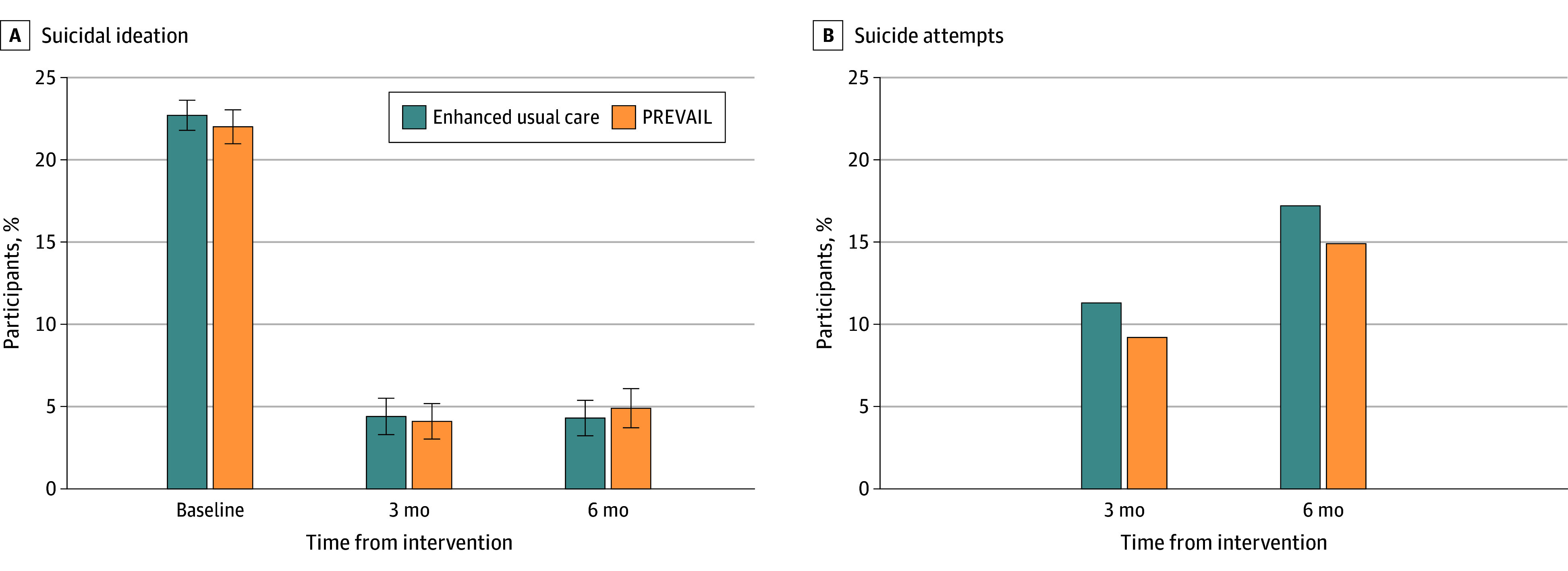
Primary Outcomes by Study Arm Suicidal ideation measured according to the Beck Scale for Suicide Ideation. Error bars indicate the 95% confidence intervals. Suicide attempts measured cumulatively as any aborted, interrupted, or actual attempts according to the Columbia Suicide Severity Rating Scale or deaths by suicide. None of the differences were statistically significant. PREVAIL indicates Peers for Valued Living.

The percentage of participants with a suicide attempt (including deaths) by 3 months was 11.3% (17 of 151) for enhanced usual care and 9.2% (13 of 142) for the PREVAIL intervention, and the number of participants with a suicide attempt over 6 months (including participants who only completed 3-month follow-up assessments) was 17.2% (28 of 163) and 14.9% (24 of 161), respectively (Figure 2). The adjusted logistic regression model showed that assignment to the PREVAIL intervention was not associated with a suicide attempt at 6 months (OR, 1.00; 95% CI, 0.91-1.09) (Table 2).

**Table 2.** Primary Analytic Models Including Intervention Indicator and Baseline Covariates

Parameter	Logistic regression of suicide attempt at 6 mo		Linear regression of suicide ideation at 6 mo	
	OR (95% CI)	*P* value	β (95% CI)	*P* value
Intercept	1.12 (0.74 to 1.68)	.59	−2.55 (−10.22 to 5.13)	.51
PREVAIL intervention (reference, enhanced usual care)	1.00 (0.91 to 1.09)	.93	0.81 (−0.57 to 2.19)	.25
Recruitment site A (reference, site B)	0.96 (0.87 to 1.05)	.37	−1.70 (−3.29 to −0.12)	.04
Man (reference, woman)	0.94 (0.87 to 1.03)	.20	1.11 (−0.64 to 2.86)	.21
Nonbinary, transgender, gender not listed (reference, woman)	0.92 (0.77 to 1.10)	.34	1.86 (−1.35 to 5.08)	.25
Unstable housing	1.10 (0.90 to 1.34)	.35	0.33 (−3.47 to 4.14)	.86
Lifetime suicide attempt	1.12 (0.96 to 1.30)	.14	1.29 (−1.17 to 3.75)	.30
Hopelessness	1.01 (1.00 to 1.02)	.06	0.25 (0.11 to 0.39)	<.001
Family support	0.98 (0.95 to 1.00)	.08	−0.30 (−0.81 to 0.21)	.25
Friend support	0.99 (0.97 to 1.02)	.71	0.10 (−0.54 to 0.73)	.76
Emotional support	1.00 (1.00 to 1.01)	.24	0.15 (0.01 to 0.29)	.03
Instrumental support	1.00 (0.99 to 1.00)	.57	−0.09 (−0.20 to 0.01)	.08
Perceived rejection	1.00 (0.99 to 1.01)	.62	0.01 (−0.11 to 0.12)	.91
Suicide ideation	NA	NA	0.09 (−0.04 to 0.21)	.16

Abbreviations: NA, not applicable; OR, odds ratio; PREVAIL, Peers for Valued Living.

In the intention-to-treat cohort, BSI scores for past-week suicidal ideation decreased significantly in both the PREVAIL and enhanced usual care arms from mean baseline scores of 22.0 (95% CI, 21.0-23.0) and 22.7 (95% CI, 21.8-23.6), respectively, to 4.1 (95% CI, 3.1-5.2) and 4.4 (95% CI, 3.3-5.4) at 3 months and 4.9 (95% CI, 3.7-6.1) and 4.3 (95% CI, 3.2-5.4) at 6 months (Figure 2). The adjusted between-group difference (PREVAIL vs enhanced usual care) in BSI was 0.81 (95% CI, −0.57 to 2.19; *P* = .25) at 6 months based on the multiple regression model. In the longitudinal data models, the interaction term between the PREVAIL arm and time was not significant for suicide attempts or suicidal ideation at either time point (eTable 4 in Supplement 2).

There were no statistically significant differences between PREVAIL engagers and enhanced usual care participants on the primary outcomes (eTable 2 in Supplement 2). The interaction between study arm and the COVID-19 pandemic was not significant for suicide attempts at 6 months (interaction coefficient [SE], −0.83 [0.66]; *P* = .21) but was significant for suicidal ideation at 6 months (interaction coefficient [SE], −3.99 [1.72]; *P* = .02). The enhanced usual care arm had a mean 6-month suicidal ideation score of 3.5 (SD, 6.1; 101 participants) before and 6.1 (SD, 7.4; 41 participants) after the pandemic, while the PREVAIL arm had a mean score of 5.0 (SD, 7.7; 92 participants) before and 4.5 (SD, 6.6; 49 participants) after the pandemic.

### Secondary Outcomes

There were no significant differences between intention-to-treat study arms on any secondary outcome measures at either time point in adjusted models. In unadjusted bivariate comparisons, PREVAIL engagers had significantly greater scores on the Hope Scale compared with enhanced usual care participants at 3 months (mean [SD], 33.4 [9.8] vs 30.6 [9.8]; *P* = .03) (eTable 2 in Supplement 2), and in adjusted analyses, engagers had significantly greater perceived social support from friends on the MSPSS at 3 months (point estimate, 0.50; 95% CI, 0.09-0.91; *P* = .02).

### PREVAIL Semistructured Qualitative Interviews

Among 132 participants interviewed, 64 (48.5%) indicated that PREVAIL had a positive impact on their suicidal ideation or likelihood of making a suicide attempt, 29 (22.0%) stated that PREVAIL had no impact, and 39 (29.5%) did not comment on PREVAIL’s impact. No participants indicated that PREVAIL worsened their thoughts or behaviors related to suicide. Table 3 provides a subcategorization of responses and example quotes.

**Table 3.** Suicide-Related Outcomes in Participant Interview Responses: Themes, Subthemes, and Example Quotes^a^

Theme	Participants, No. (%)	Example quote
Peer had positive impact on suicide risk		
Less likely to act on thoughts of suicide	23 (17.4)	“Yes, she decreased the likelihood of me attempting again talking to her about her life now. It’s one thing when someone says it gets better, but to know someone who it actually did get better for is really nice.” “I think it changed the likelihood of me (making a suicide attempt) because I am more aware of the support around me.”
Changed how one thinks about suicide	18 (13.6)	“[Peer] was really great at reframing my thoughts about it. We had talked about acceptance and helped me rethink my mental health. It was helpful.” “She helped me see the suicidal tendencies or the thoughts are like tunnel vision. And that it’s really easy to get sidetracked that you’ve come to the end of the road. That’s not my reality. That’s not even what other people see of me. And it’s not easy to tell yourself that until you meet someone who you can relate to sometimes.”
Impacted suicide risk by increasing hope	10 (7.6)	“I think it helped keep me safe. I didn’t think of things differently, but it made me feel like it is possible to have a life without thinking of suicide. It helped me see that maybe things can be less awful.” “She encouraged me. Her encouragement helped me to keep going.”
Impacted suicide risk by increasing sense of belongingness	6 (4.5)	“I think that it really helped me realize how many people there are that do care. Even if it doesn’t seem like it. It put it in perspective that somebody is willing to help strangers and made me realize the thought nobody cares about me is false.” “The fact that she had similar experiences. Made me feel like I’m not alone. Not sure if a therapist can relate in the same way.”
Positive impact in general (no specific reason given)	6 (4.5)	“I don’t know that it changed my thinking, but it certainly reinforced my desire to live.” “Yes. She definitely changed my outlook on everything. For the better. She was wonderful.”
Peer had no impact on suicide risk	29 (22.0)	“Truthfully, it really didn’t. It was nice to work with him, I guess. But as far between that and my therapist, obviously my therapist was more helpful as far as my mental health and well-being. And following my suicide plan with my therapist after being in the hospital was more helpful and important than just talking to the peer. It was nice to have someone who understood though because sometimes even my therapist doesn’t understand.” “I don’t think it really did. I had a lot of tools and methods to help prevent suicide or maintain mental health and well-being, but my anxiety problems led me to drink and put me in a dark place. I really think the alcohol drove me to places of suicide. Once I started abstinence from alcohol, I’ve never really had those thoughts. And I remember my tools.”
Did not comment on peer’s impact on suicide risk	39 (29.5)	NA

Abbreviation: NA, not applicable.

^a^ No participants who completed the participant interview reported that the PREVAIL intervention worsened their thoughts or behaviors related to suicide in the qualitative debriefing responses.

### Participant Deaths and Adverse Events

Two participants died by suicide, both in the PREVAIL arm. Both participants met only once with the peer while still admitted to the inpatient unit, expressed no concerns to study staff at follow-up immediately after discharge, were unresponsive to peers’ subsequent contact attempts, and died within 2 to 3 weeks after discharge. There were 2 study-related adverse events. One participant withdrew from the study and attributed relapse to alcohol use and presentation to psychiatric emergency in part to negative interactions with a peer, and another participant stated that the loss of peer support at the end of the PREVAIL intervention partially contributed to a medically serious suicide attempt. Two other participants declined further participation in PREVAIL after reporting negative peer interactions (eg, excessive discussion of religion) that did not result in an adverse event. All participants were offered referral information for mental health treatment resources.

## Discussion

In this randomized clinical trial, PREVAIL participants had fewer suicide attempts during the follow-up period compared with enhanced usual care participants, although these differences were not statistically significant. Two deaths by suicide occurred in the intervention arm compared with none in the enhanced usual care arm, though these occurred during the highest risk period immediately following hospital discharge^36^ and prior to engaging in the substantive components of PREVAIL beyond an introductory meeting with a peer. No statistically significant effects were seen for PREVAIL on suicidal ideation or a wide range of secondary outcomes. Positive findings included improved hope and perceived support from friends at 3 months among PREVAIL engagers compared with enhanced usual care participants. Although improved hope is consistent with other peer support intervention trials,^8,9,10^ the causal direction between engagement and these outcomes is uncertain. Post hoc analyses also suggested that the PREVAIL intervention may have buffered against increased suicidal ideation seen among the enhanced usual care participants during the COVID-19 pandemic, though potential mechanisms (eg, greater social support) underlying this finding requires further exploration.

Overall null effects of the intervention may indicate that usual care is sufficient for reducing suicide risk, although the finding that approximately 1 in 6 participants in both arms attempted suicide over 6 months suggests that improvements to usual care are still needed. Potential reasons for the lack of effectiveness of PREVAIL include insufficient intensity (approximately 1 hour a week for 3 months), lack of integration with the patient’s other mental health care, and redundancy of PREVAIL content (eg, goal setting) with services participants already received from therapists. Although we found no evidence that PREVAIL was harmful across comparisons of primary and secondary outcomes, the intervention may not have had the intended beneficial effects on average if some patients found the peer meetings helpful while others found them counterproductive. Hearing another person’s story of recovery from mental health challenges could be a source of hope and connection for some, while others may have difficulty relating to others’ experiences or feel burdened when they are shared.^37^ While nearly one-half of PREVAIL participants who completed semistructured interviews reported that the intervention had a positive impact on their suicidal thoughts and none reported a negative effect, 2 deaths by suicide occurred in the PREVAIL arm after 1 meeting with a peer, 3 participants dropped out of the study due to negative peer interactions, and 2 attributed worsening mental health to peer interactions. Increasing the intervention intensity, greater integration of peer support with patients’ ongoing care, developing novel content for peer meetings, identifying which patients are most likely to benefit from peer support, and minimizing negative interactions with peers may be necessary for effectiveness. Additional research is also needed to identify and mitigate the potential negative impact of ending a course of peer support among vulnerable patients.

### Limitations

Limitations of the study include that participants were not masked to the study condition after completing their baseline assessments. Participation in follow-up assessments or interviews may have been biased by perceptions of the intervention, and overall rates of completion of follow-up assessments were low. Providing peer support in a research context (ie, audio recording each meeting, suicide risk assessment protocols) may have affected the quality of the peer-participant relationship. Findings may not generalize to populations outside of Michigan.

## Conclusions

This randomized clinical trial found that providing 3 months of individual peer support to psychiatric inpatients who had attempted suicide or with suicidal ideation was not effective beyond usual care for improving suicide-related outcomes or interpersonal risk factors. Given that peer specialists are integrated into services for individuals at increased risk for suicide, alternative peer support interventions for suicide prevention (eg, with different session content) deserve further study.

## Notes

Supplement 1.
Trial Protocol

Supplement 2. **eTable 1.** Full Demographic Characteristics, Including Missing Values **eTable 2.** Complete Baseline and Follow-Up Measure by Study Arm and Engager Status **eTable 3.** Missing Values by Study Arm and Engager Status **eMethods.** Generalized Linear Mixed-Effects Model Methods and Results **eTable 4.** Generalized Linear Mixed-Effects Models of Primary Outcomes Across All Assessment Time Points

Supplement 3.
Data Sharing Statement
