# Supplementary material for: Peer Support Intervention for Suicide Prevention Among High-Risk Adults in Michigan: A Randomized Clinical Trial
Source: JAMA Netw Open. 2025 May 28;8(5):e2510808. doi: 10.1001/jamanetworkopen.2025.10808 (PMC12120652; doi:10.1001/jamanetworkopen.2025.10808)
Supplement: Supplement 1. — Trial Protocol [file jamanetwopen-e2510808-s001.pdf]

Trial Protocol and Statistical Analysis Plan: Peer Support Intervention for Suicide Prevention among High-Risk Adults: A Randomized Clinical Trial

Contents:

A. Trial Protocol and Statistical Analysis Plan (initial approved version)
B. Trial Protocol and Statistical Analysis Plan (final version)
C. Protocol Reference List
D. Summary of Protocol Amendments

A. Trial Protocol & Statistical Analyses Plan (Initial)

Version Date: February 20, 2018

BACKGROUND & RATIONALE

Background. After years of steady increases, in 2014 the suicide rate in the U.S. reached its highest rate in decades with over 42,000 suicide deaths in that year.(2) Suicide has surpassed motor vehicle accidents as a leading cause of death in the U.S., is the tenth leading cause of death overall, and is in the top 4 leading causes of death for those younger than 65.(17) For every suicide death, approximately 25 times as many people (over 1 million Americans) attempt suicide, resulting in emergency department visits or hospitalizations costing the U.S. $10.4 billion dollars in health care expenditures and lost productivity.(18) The costs of suicide extend to include negative health and social functioning effects on surviving loved ones.(19) The strategic action plan for suicide prevention in the U.S. emphasizes the need for a combination of universal, selective, and indicated interventions.(1) Successful universal strategies include restricting public access to various means for suicide such as firearms, jump points, and toxic substances.(20-23) Other population-level approaches such as gatekeeper training (e.g., training public service professionals to identify and refer individuals at risk for suicide), universal screening in health systems, or crisis telephone/internet services have generally low levels of evidence (i.e., few randomized controlled trials) for preventing suicides or suicide attempts despite instances of wide scale implementation.(4, 24-26) Universal strategies that include linkage of at-risk individuals to additional selective or indicated prevention services may depend on the effectiveness of those subsequent services. However, there are very few interventions shown to be effective for high-risk adults, and even fewer that have been widely adopted by health systems. (4, 27) Selective or indicated prevention interventions include use of brief follow-up contacts (e.g., by phone, mail, or face-to-face) by health professionals after a suicide attempt or hospital discharge. (28-31) The findings of these studies are mixed and limited by samples of only treatment refusers or those without basic access to mental health treatment, though they suggest that even relatively minor expressions of caring may improve some recipients' belongingness. The medications lithium and clozapine have been shown to reduce suicides among those at risk due to a mental health condition, though their side effect burden, risks of serious medical complications, and indications for only a subset of mental health conditions limit their use.(32, 33) Various psychotherapies, including dialectical behavioral therapy, cognitive behavioral therapy, and problem-solving therapy have been shown to reduce the incidence of suicide attempts, though a pooled analysis of these trials demonstrated no effect on suicide deaths.(34) Psychotherapy as a mental health service also faces major barriers related to workforce availability and patient adherence.(35, 36) Receipt of psychotherapy specifically for suicide prevention is not known; however, it is almost certainly underutilized considering less than 20% of patients receive an adequate course of *any* psychotherapy after psychiatric hospitalization.(37, 38) The steady rise in suicide rates is a testament to the need for interventions, such as peer mentorship, that offer new approaches to addressing suicide risk and have potential to be implemented at wide scale.

Peer mentorship and the spectrum of peer support. Peer mentors are individuals who have achieved stable recovery from a mental health condition and who incorporate their personal experiences into providing support to others currently experiencing mental health challenges. Peer mentorship exists within a spectrum of peer support and occupies a middle ground between less structured mutual/reciprocal peer support and more structured forms where peers provide services traditionally delivered by clinically trained professionals (e.g., case management) and rely less on their lived experience of mental health challenges. (Figure 1). (5)

Figure 1. Continuum of Helping Relationships Including Peer Support (adapted from Davidson(5))

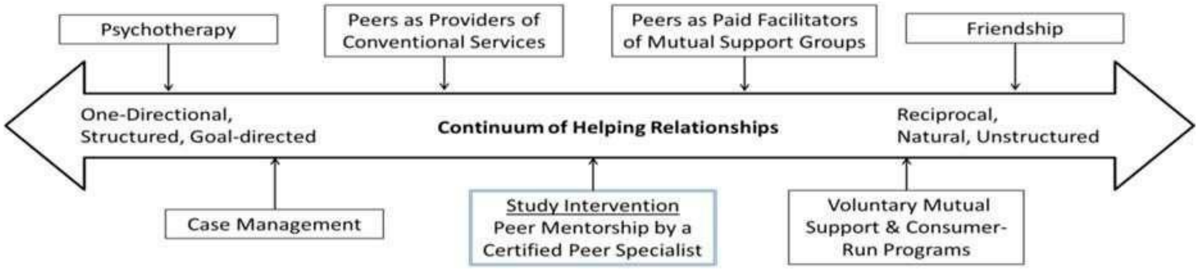

We chose a mentorship model of peer support for the PREVAIL intervention rather than mutual peer support because in a recent study we found a mutual one-to-one peer support intervention was no more effective for depression (a risk factor for suicide) or suicidal ideation than usual care.(39) A group-based mutual support intervention for VA patients with any mental health diagnosis also had null effects with respect to depression, functioning, hope, social support, and self-harm.(40) In contrast, peer mentorship has demonstrated effectiveness for post-partum depression compared to usual care, and peer mentors are as effective as traditional providers of case management, suggesting that peers who can speak to and role model successful recovery may be particularly effective.(8, 41) We did not choose a model where peers act as providers of traditional services because the disclosure of the peer

mentor's own experiences with suicide and recovery is core to the distinct mechanisms by which the intervention might reduce suicide risk (see below).

Intervention mechanisms of peer mentorship. Our theoretical model for peer mentorship as a method for preventing suicide attempts draws from the interpersonal theory of suicide (ITS) and the postulated effects of peer support (Figure 2) (3). The ITS groups empirically-supported suicide risk factors into four domains: hopelessness, thwarted belongingness, burdensomeness, and acquired capability for suicide. The theory posits that active suicidal ideation (e.g., thoughts of killing oneself) occurs when belongingness and burdensomeness occur in the presence of hopelessness. Suicide attempts occur in an actively suicidal individual who also has an acquired capability for suicide. The ITS domains explain a greater portion of the variance of suicidal ideation and attempts than models using demographic characteristics and diagnoses (42). While the theorized interactions between domains have not been consistently replicated, the independent effects of the ITS domains have been repeatedly found to be associated with suicide outcomes, thereby supporting the use of these constructs as targets of this intervention. (43, 44)

Figure 2. Interpersonal Theory of Suicide (adapted from Van Orden(3)) and Peer Mentorship

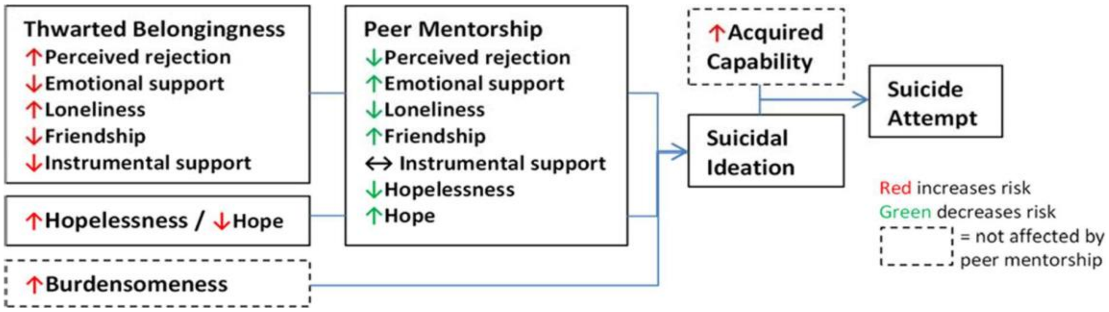

There are many postulated benefits of peer mentorship, notably including improved belongingness achieved by establishing shared experiences of mental health challenges and decreased hopelessness achieved as the peer mentor provides a role model of recovery. While the effects of poor belongingness (e.g., perceived rejection, loneliness, low emotional support) and hopelessness on increased risk of suicide are well established according to several reviews encompassing dozens of trials(3, 45-47), whether peer mentorship improves an individual's sense of belongingness or hopelessness has not been shown definitively as most trials of peer support have not included measures of these constructs.(48) In our review of 40 trials of peer support cited in 3 recent meta-analyses, 17 studies included measures of belongingness with 6 showing positive effects, and 4 studies included measures of hope or hopelessness with 3 showing positive effects.(49- 51) Based on the suggestive but inconsistent evidence that peer mentorship can improve belongingness and hopelessness, PREVAIL was designed with explicit structured content (e.g., identifying reminders of hope) to address these risk factors. In addition to the core mechanisms of suicide prevention drawn from the ITS, peer mentorship can potentially further reduce the risk of suicide through several other mechanisms including: monitoring and detection of escalating risk, greater activation and engagement in treatment, and improved self-management of mental health symptoms. (52) Aim 1 of this study will determine whether the aggregate benefits of peer mentorship are effective for reducing suicide attempts, while Aim 2 will assess whether the intervention has the hypothesized effects on hopelessness and belongingness. (Note: hope and hopelessness are used interchangeably in the writing of this proposal for brevity, as are thwarted belongingness and belongingness, but each may represent separate but correlated constructs and thus will be measured separately).

Implementation of an effective peer support intervention. Although peer support services began historically as an alternative to psychiatric treatment, there has been increasing integration of peer services into mental health treatment with the advent of professionalized, certified peer specialists. (5, 53) Peer specialists are individuals with a lived experience of mental illness who receive formal training, certification, and employment in providing peer support. PREVAIL was designed to be delivered by peer specialists in part because they are already integrated with many community mental health programs and provide a wide range of recovery-oriented, patient-centered services. (54) The State of Michigan has trained and certified 1,640 peer specialists, and an estimated 24,000 have been certified nationwide. (12) In 2014, 34 states also offered Medicaid reimbursement for peer support services, furthering sustainability of these services. (55) The State of Michigan also trains peer recovery coaches, individuals who have lived experience receiving public mental health services specific to substance use, and who serve similar populations in community mental health programs; Michigan offers reimbursements for peer recovery coach services through public substance abuse treatment funds. With the integration of peer providers into mental health treatment services, programs are increasingly involving peers in crisis services. (56-58) However, peer specialist certification training and peer recovery coach training does not currently address suicide or how peers might be most useful to individuals with suicidal ideation or behaviors. Thus, there is likely substantial demand for an effective protocol for peer providers who are supporting high-risk individuals. The training director for peer specialists in the State of Michigan, who has supported the development and study of PREVAIL, would offer training in the intervention immediately to currently employed peer specialists if found effective, as would potentially many other peer training directors. However, the decision by peer specialists, community mental health clinics, and hospitals to implement PREVAIL (if effective) could be negatively or positively influenced by a variety of factors. Prior studies of peer support implementation (59, 60) (including by consultant Chinman) identified confusion about the peer's role within a clinical team as a barrier. Whether the PREVAIL training and

protocols address this barrier, and whether other factors rise in prominence when accounting for the health system perspective (e.g., effect on readmissions) or the context of suicide prevention (e.g., acceptability of safety protocols) requires further, structured examination.(61) Examination of implementation concurrent with effectiveness (i.e., the hybrid trial design) will help explain trial findings if null and will increase population health impact of PREVAIL by reducing delays in implementation if effective.(14)

Preliminary Studies

PREVAIL R34 Pilot Study. All protocols necessary to conduct the proposed fully powered randomized controlled trial to test the effectiveness and mechanisms of PREVAIL have been successfully pilot tested.

Intervention development and peer mentor training. During the intervention development phase of the R34 project, an expert panel including suicide prevention researchers (King, Ilgen, Holloway), peer support researchers (Valenstein, Chinman), peer specialists (Coe, Derosa), the peer support training director for the State of Michigan (Werner), and an inpatient psychiatry unit director (Hirshbein) were convened to create an outline of the essential training needs for peer mentors to work with high-risk patients and address suicide risk according to the interpersonal theory of suicide. The outline of the training and intervention content was then used as the basis for developing the training manual (Other Attachment IIA). This manual was developed by the PI (Pfeiffer) in conjunction with Eduardo Vega and Dequincy Jones of the Center for Dignity, Recovery, and Empowerment (a peer support advocacy, service, and research organization); Kristen Abraham, a clinical psychologist with expertise in peer support; and Debra Levine, a clinical psychologist and post-doctoral fellow. Drafts of the training materials were circulated among the expert panel and revised in an iterative process. The training was then delivered over 3 days to the study's 4 peer mentors utilizing adult learning theory with small group discussions, role plays, and video vignettes. (The term "peer mentor" indicates a peer specialist or peer recovery coach with PREVAIL training.)

Figure 3. Consort Diagram for PREVAIL Pilot Study

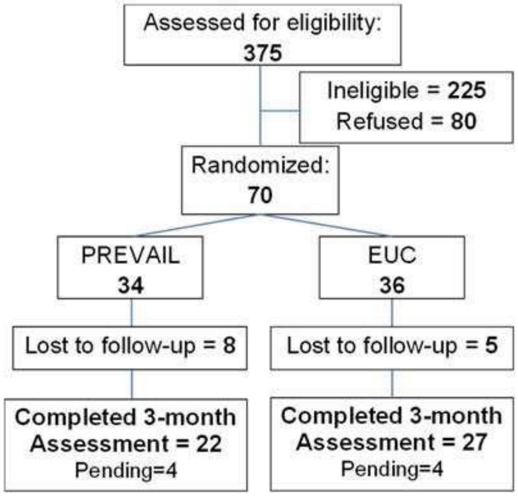

Acceptability and feasibility. A "pre-pilot" in which each peer mentor delivered the intervention to 1 or 2 participants was conducted; the only significant change to the study protocol was to allow mobile device and social media communication with participants. The pilot RCT phase of the study was launched and successfully enrolled 70 participants (Figure 3). 375 patients with medical record documentation of suicidal ideation or suicide attempt at admission were screened, 225 of whom were not eligible and 80 of whom refused participation. The most frequent reasons for ineligibility were unstable psychosis, cognitive disorder, or severe personality disorder as determined by the attending psychiatrist (n=89), distance (n=30), and prolonged hospitalization due to receipt of electroconvulsive therapy (n=30). Participation in the intervention exceeded our target thresholds with 95% of intervention participants completing a meeting with the peer mentor prior to discharge (target 80%) and a mean completion of 5.6 (SD 4.8) peer encounters over 3 months (target mean of 4). The most common reason for not completing any encounters after discharge (n=9) was feeling overwhelmed or too busy; participants were offered to switch to a new peer mentor, but none chose this option, suggesting the reasons for poor engagement were not specific to the individual peer mentors. Excluding participants pending follow-up (pilot study is still collecting follow-up data), 79% (49 of 62) have completed the 3- month assessments and 80% (28 of 35) have completed the 6-month study assessments. Exceeding our target of 60 for enrollment and target of 75% for follow-up assessments demonstrates feasibility to proceed to a larger-scale trial.

Supervision, fidelity, and refinement of the intervention. Fidelity to the intervention, including suicide safety protocols, was maintained by weekly group supervision meetings consisting of the peer mentors and the principal investigator (a psychiatrist) or a co-investigator psychiatrist or psychologist. Each participant encounter was discussed, training materials and intervention protocols were reviewed, and audio-recordings of individual sessions were presented for feedback. Following the initial phases of the trial, the structure of the intervention session content was refined to facilitate fidelity by establishing a consistent format across content areas. The format used the mnemonic "ILSM" which stands for "Invite, Learn, Share, Motivate", and a semi- structured conversation guide was created for each content domain in this format (Other Attachment IIB). The format incorporates motivational interviewing concepts and techniques—encouraging peers to first understand the participant's experiences and ideas for change with intentional sharing of the peer mentor's experiences to offer new ideas and validate shared struggles. The ILSM conversation guides were developed collaboratively with the 4 peer mentor interventionists and study co-investigators. A fidelity measure of adherence to the ILSM format was developed and used to rate sessions via audio-recordings (Other Attachment IIC). The measure, based on the Yale Adherence and Competence Scale(62), uses a 4-point scale to rate the skill (1 representing "very poor" to 4 representing "skilled") and extensiveness (1 representing "not discussed" to 4 representing "a lot of discussion" [e.g., >15 minutes]) with which ILSM conversations are delivered. A rating of 2 or greater on both skill and extensiveness was considered adequate fidelity to the

intervention. Fidelity to general communication and peer support skills (e.g., validation, sharing, avoidance of medical advice) were also rated on the 1 to 4 point "skill" scale with a score of 3 or greater indicating fidelity to the intervention. Two research assistants were trained on use of the fidelity measure until inter-rater agreement was >80%. Among 20 sessions rated using the measure, 85% demonstrated adequate fidelity to the ILSM model and 72.5% of general skills were performed with adequate fidelity. These ratings clearly demonstrate peer mentors were able to incorporate PREVAIL content into their contacts with participants.

Outcomes and safety. Comparisons of outcomes between the intervention and enhanced usual care control groups were not conducted because any finding would be an unreliable estimate of the true effect given the small sample size.(11) The R34 randomized control group allowed pilot testing of all necessary protocols in preparation for this R01 study. Across the entire sample, 24% of participants reported any post-enrollment suicide attempt at 3 months according to the Columbia Suicide Severity Rating Scale.(63) Suicidal ideation according to the Beck Scale for Suicidal Ideation improved from 23.3 (SD 7.6) at baseline to 6.6 (SD 7.7) at 3 months.(64) Despite limited power, improvements on belongingness measures of loneliness and perceived rejection, but not emotional support, were statistically significant at 3 months.(65) There was also statistically significant improvement on a measure of hopelessness, but not hope, at 3 months.(66, 67) These findings demonstrate feasibility of measuring the primary outcomes and potential mediators in a larger trial. A data and safety monitoring board (DSMB) was convened every 6 months to review adverse events. Two participants were readmitted after study staff contacted Emergency Medical Services due to acute suicide risk, and in one of these cases the participant had already overdosed on medication when she met with the peer mentor. One participant randomized to the enhanced usual care condition died by suicide within 2 weeks of enrollment; his only interaction with the study was the enrollment meeting and baseline assessment on the inpatient unit. No adverse events were attributed to study participation. Peer mentors were not treated differently from other research staff in terms of monitoring their mental health; however, there were no incidents in which the mental health of the peer mentors interfered with their work.

Other Studies of Peer Support. Drs. Pfeiffer and Valenstein have conducted prior multi-site RCTs involving peer support, including an ongoing trial that utilizes peer specialists to enhance engagement in computer- based cognitive behavioral (c-CBT) therapy for depression and a completed trial of mutual dyadic peer support among depressed VA patients. The latter of these trials found no differences in improvement in depression among participants randomized to be paired for purposes of mutual peer support compared to those randomized to usual care.(39) These findings directly informed the use of a peer mentor model vs. a mutual support model for the PREVAIL intervention. Drs. Pfeiffer and Valenstein also conducted a single-arm VA pilot study of peer support by a peer mentor following psychiatric hospitalization for depression, which informed the need for the more extensive, suicide-specific training program developed during the R34.(68)

Other Studies of Suicide Risk & Prevention. Dr. King is internationally recognized for her expertise in the conduct of RCTs of suicide prevention interventions for youth and young adults, including completion of a randomized controlled trial of a social network intervention.(69,70) Dr. King has also contributed substantially to our understanding of risk-management and ethical considerations in intervention research to prevent suicide.(71) Drs. King and Pfeiffer served as co-investigators on a DoD-funded randomized controlled trial of a CBT-based suicide prevention trial (PI: Holloway). The core CBT intervention in the study was administered while patients were admitted to a military psychiatric hospital, and Drs. King and Pfeiffer led the development of the post-discharge therapist-delivered booster sessions. Co-I Dr. Ahmedani is a nationally recognized suicide prevention researcher who has investigated patterns of care within health systems prior to suicide and is an advisor to the National Zero Suicide Initiative.(72) Drs. Pfeiffer, Valenstein, and Kim have also previously examined the associations between hopelessness, belongingness, and suicidal ideation.(44)

Implementation and Qualitative Studies. Co-I Dr. Forman is a qualitative methodologist and implementation scientist who has worked with Drs. Pfeiffer and Valenstein on a qualitative evaluation of the implementation of the Buddy 2 Buddy peer support program in the Michigan National Guard and a mixed methods study of barriers and facilitators to receipt of quality depression care following psychiatric hospitalization across two VA medical centers.(36, 73) Dr. Forman is a Co-I on a VA peer-supported c-CBT study which uses a similar hybrid effectiveness-implementation design to the current study. Consultant Laura Damschroder is an implementation scientist and the lead developer of the Consolidated Framework for Implementation Research (CFIR), the conceptual framework guiding Aim 3 of this study.(61) Dr. Forman and Ms. Damschroder have collaborated previously on implementation studies. (74-76) Consultant Dr. Chinman has conducted a series of studies regarding the implementation of peer support services, including the integration of peer specialists into mental health treatment settings across the VA health system. (53)

Study Rationale. New approaches to suicide prevention are needed to meet the rising tide of suicide deaths in the U.S. Peer mentorship represents a novel approach to reducing suicide risk by addressing poor belongingness and hopelessness, two risk factors for suicide supported by theory, evidence, and advocates. This study of peer mentorship will be the first effectiveness trial of a peer-delivered intervention designed to reduce suicide attempts and suicidal ideation. This study advances peer support research by the inclusion of intermediate targets in the intervention design and assessment of potential mediators. A safe and effective peer-based intervention to prevent suicide attempts could achieve wide scale implementation due to a growing, professionalized, and integrated peer specialist workforce that increasingly supports high-risk individuals. The hybrid effectiveness-implementation design used in this study is an innovative approach to reducing delays in translating evidence from clinical trials into practice. A major limitation to clinical trial research is that widespread implementation of effective interventions may take decades or never occurs after publication

of the trial results. (13) Hybrid effectiveness-implementation trial designs are intended to address this problem by studying the barriers and strategies for future implementation simultaneous to the determination of effectiveness. (14) Even if the specific intervention proves not to be effective, assessment of stakeholder experiences with the intervention may provide explanations for the null findings, guide future research, and inform ongoing initiatives to increase peer involvement in suicide prevention.

STUDY DESIGN

We will conduct a single-blind randomized controlled trial of the PREVAIL peer mentorship for suicide prevention intervention compared to enhanced usual care among 490 participants at high-risk for suicide recruited from inpatient psychiatric units.

Study Flow

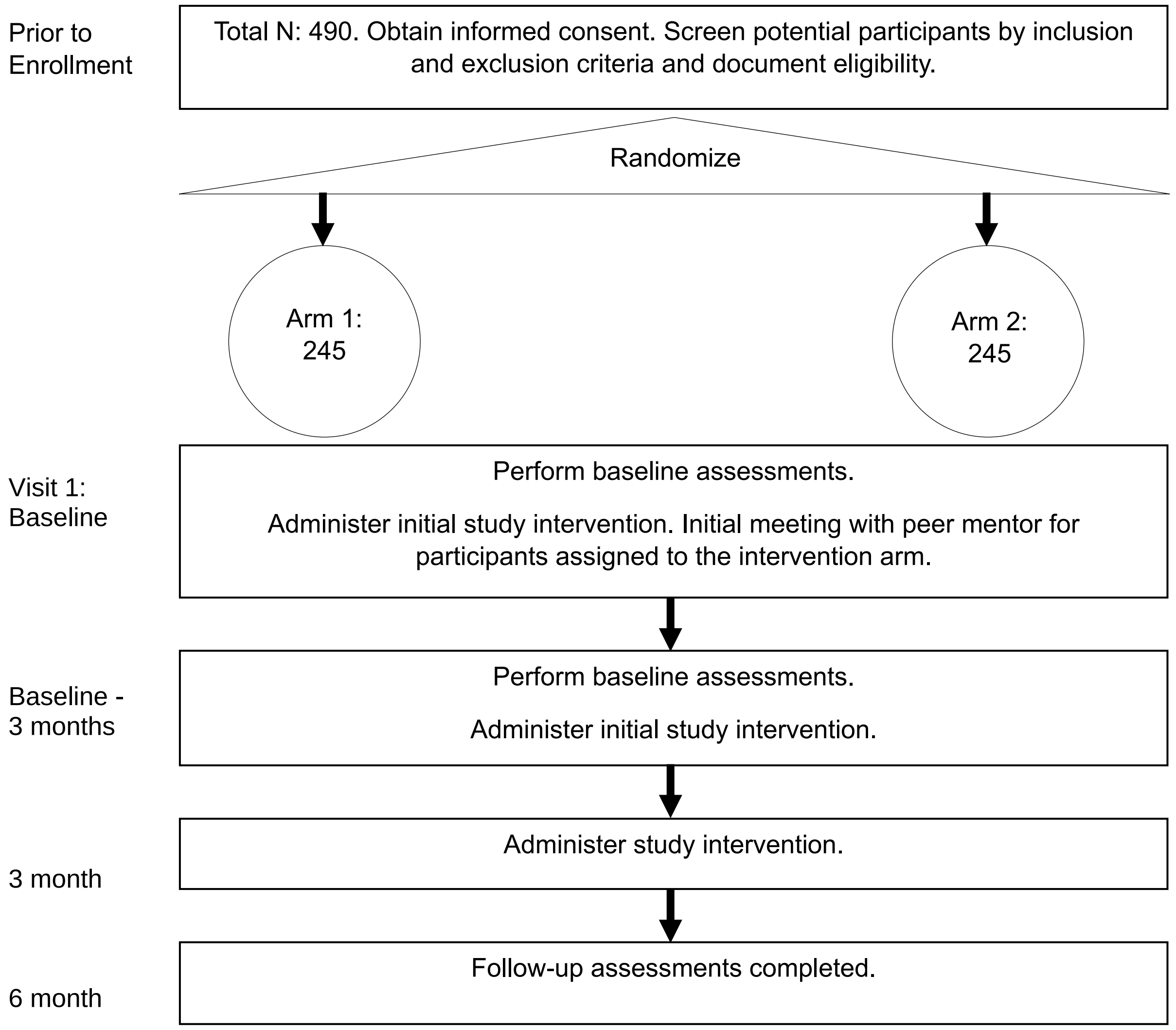

STUDY PROCEDURES

Schedule of Activities

	Pre-screening (Pre-Consent)	Enrollment - Week 1, Day 1	Baseline - Week 1, Day 1	Intervention - Week 1	Intervention - Week 2	Intervention - Week 3	Intervention - Week 4	Intervention - Week 5	Intervention - Week 6	Intervention - Week 7	Intervention - Week 8	Intervention - Week 9	Intervention - Week 10	Intervention - Week 11	Intervention - Week 12	Intervention - Weeks 12-16	Intervention - Weeks 16-23	Intervention - Week 24	Withdrawal
Review eligibility	X																		
Eligibility confirmation		X																	
Informed consent		X	X																
Outcome Evaluation																			
Suicide Attempts (Columbia Suicide Severity Rating Scale)			X												X			X	
Suicidal Ideation (Beck Suicide Scale)			X												X			X	
Medically serious suicide attempts (medical record)															X			X	
Self efficacy (Self Efficacy to Avoid Suicidal Action Scale)			X												X			X	
Hopelessness (Beck Hopelessness Scale)			X												X			X	
Quality of life (Quality of Life Enjoyment and Satisfaction Questionnaire Short Form)			X												X			X	
Functional status (Short Form 12)			X												X			X	
Perceived social support (Multidimensional Scale of Perceived Social Support)			X												X			X	
Depression (Patient Health Questionnaire (PHQ-9))			X												X			X	
Hope (State Hope Scale)			X												X			X	
Burdensomeness (Interpersonal Needs Questionnaire)			X												X			X	
Perceived emotional support (NIH Toolbox Adult Social Relationship Scales)			X												X			X	
Perceived instrumental support (NIH Toolbox Adult Social Relationship Scales)			X												X			X	

Perceived friendship (NIH Toolbox Adult Social Relationship Scales)			X												X			X	
Loneliness (NIH Toolbox Adult Social Relationship Scales)			X												X			X	
Perceived rejection (NIH Toolbox Adult Social Relationship Scales)			X												X			X	
Utilization of health care services (NIH Toolbox Adult Social Relationship Scales)			X												X			X	
Medication adherence (Single-Item Self Rating Scale for Medication Adherence)			X												X			X	
Perceived meaning in life (Meaning in Life Questionnaire)			X												X			X	
Randomization			X																
Enhanced Usual Care & Experimental Interventions – Peer mentorship			X	X	X	X	X	X	X	X	X	X	X	X	X	X			
Event reporting		X	X	X	X	X	X	X	X	X	X	X	X	X	X	X	X	X	X

DESCRIPTION OF ACTIVITIES

<u>Pre-consent activities</u>

Pre-screening - Each day, study staff at each recruitment site will review the medical records of patients admitted to their inpatient psychiatric unit to determine if they meet preliminary inclusion criteria. Patients will be eligible for the study if they: 1) are age 18 years or older, 2) are currently admitted to an inpatient psychiatric unit and have medical record documentation of suicidal ideation or suicide attempt at the time of admission, 3) have a Beck Suicide Scale score of 5 or higher for the 1-week period prior to admission, 4) are fluent in English, and 5) are able to be reached reliably by telephone.

Eligibility confirmation - Study staff will contact the attending psychiatrist of each patient who meets preliminary inclusion criteria. The attending psychiatrist will determine if peer mentorship is not appropriate due to unstable psychosis, cognitive disorder, or severe personality disorder. Study staff will approach patients who remain eligible, and will administer the Mini-Cog to determine if the patient is unable to provide informed consent due to substantial cognitive impairment.

Informed consent - Study staff will obtain informed consent from interested individuals before they are discharged from the inpatient unit. The study staff member will review all study activities, emphasize that participation is voluntary, discuss potential risks and benefits of participating, and review the participant's responsibilities. An individual is considered enrolled once informed consent has been provided.

<u>Post-consent activities and outcomes assessments</u>

Outcome assessments are performed at three time points across an individual's participation in the study: baseline (while the participant is admitted to the inpatient unit), 3 months, and 6 months from their enrollment in the study. Beck Hopelessness Scale, Beck Scale for Suicidal Ideation, Columbia Suicide Severity Rating Scale, State Hope Scale, Interpersonal Needs Questionnaire, Multidimensional Scale of Perceived Social Support, Meaning of Life Questionnaire, Single-Item Self Rating of Medication Adherence, NIH Adult Toolbox Social Relationship Scales, Patient Health Questionnaire (PHQ-9), Quality of Life Enjoyment and Satisfaction Questionnaire Short Form, Self-Efficacy to Avoid Suicidal Action Scale, Working Alliance Inventory – Client, Short Form 12, and health service utilization inventory (lifetime or 3 month interval) – these will be collected via self-reported measures at baseline, 3 month, and 6 month assessments.

Randomization and Blinding. As was done in the R34, randomization will be conducted using a minimization algorithm implemented by the University of Michigan's Consulting for Statistics, Computing & Analytics Research (CSCAR) center.(80) The two variables included in the minimization are gender and whether the participant made a suicide attempt immediately prior to hospital admission. These variables were chosen to avoid the potential confounding that could arise if by chance these variables were not equally distributed during random assortment.(81) Randomization will be stratified by study site. After a participant has completed consent procedures and the baseline assessment, a research assistant will access the CSCAR web application, input the two variables, and receive the allocation assignment. All follow-up assessments will be completed by a separate research assistant who will be blinded to study arm assignment.

Control & Experimental Interventions. Once study arm is ascertained, the study team will commence with study intervention activities. Full details of each therapy are described in Section 5. Briefly, the study staff member will schedule the participant's 3 month assessment and provide them with a copy of the Informed Consent Document, and then either (1) the baseline visit will be complete, or (2) the study staff member will instruct the participant to select a peer mentor from a list of short written descriptions of peer mentors currently available to accept a new patient, and will coordinate an initial meeting between the participant and their selected peer mentor to take place on the inpatient unit before the participant is discharged.

Outcome Assessments. The 3 and 6 month outcome assessments should occur within the windows listed on the schedule of activities, through in-person meetings with separate research staff blinded to study arm assignment. In cases where in-person attendance is not feasible, the measures can be completed over the phone or internet, though in these cases the participant's location and availability of an emergency contact person will first be confirmed in the event of acute suicidality. If a participant fails to complete an assessment within the prescribed window, that assessment will be documented as incomplete.

Withdrawal. If participants withdraw or decline to finish the peer mentorship intervention, every effort will be made to gather primary outcome data (Columbia Suicide Severity Rating Scale, Beck Scale for Suicide Ideation). These surveys may be done in person or over the phone.

SELECTION AND ENROLLMENT OF PARTICIPANTS

Inclusion Criteria. The goal of the study inclusion and exclusion criteria is to identify a broadly representative sample of inpatient psychiatric patients at high risk for suicide based on a suicide attempt or suicidal ideation prior to admission. Including only those with a suicide attempt would limit the generalizability of the intervention. Recruitment from inpatient units facilitates the initial in-person peer support contact in a safe setting and ensures some intervention has taken place prior to when patients are at maximal risk immediately

following discharge.(77) Patients will be eligible for the study if they: 1) are age 18 years or older, 2) are currently admitted to an inpatient psychiatric unit and have medical record documentation of suicidal ideation or suicide attempt at the time of admission, 3) have a Beck Suicide Scale score of 5 or higher for the 1-week period prior to admission, 4) are fluent in English, and 5) are able to be reached reliably by telephone. The age and language criteria are necessary because peer mentors will be English-speaking adults.

Exclusion Criteria. Patients will be excluded if they are: 1) substantially cognitively impaired (according to the Mini-Cog(78, 79)), 2) unable to provide informed consent for any reason (including incompetency), 3) determined by the patient's attending psychiatrist that peer mentorship is not appropriate due to unstable psychosis, cognitive disorder, or severe personality disorder, 4) already receiving or intending to receive peer mentorship (i.e., sponsor from Alcoholics Anonymous) or group-based peer support on a biweekly or more frequent basis, 5) residing more than 50 miles from any peer mentor, 6) planning to be discharged to another inpatient or residential facility, or 7) receiving electroconvulsive therapy (which prolongs the hospital stay and complicates informed consent and peer interactions due to risks of memory impairment).

Recruitment. Participants will be recruited from the University of Michigan Health System's inpatient psychiatry unit (UM) and the Henry Ford Health System's Kingswood psychiatric hospital (HF). The UM 25- bed unit was the primary source of recruitment for the R34 pilot study, whereas the HF site was added near the end of the pilot study to demonstrate feasibility of the intervention in more than one setting, ensure adequate accrual rates for a full scale trial, and increase the sociodemographic diversity of study participants to improve generalizability of findings. The UM site admits approximately 800 patients per year, primarily from the hospital's Psychiatric Emergency Service, the largest such service for Washtenaw County. Kingswood is a 100-bed hospital located in Oakland County in a suburb of Detroit. We will recruit from a 28-bed adult unit of Kingswood that admits approximately 1200 patients per year. Recruitment from these sites during the R34 pilot study resulted in a population with severe suicidal ideation and frequent prior suicide attempts that is representative of the population in Michigan in terms of race/ethnicity (Table 1). Directors of both recruitment sites strongly endorse the study (see Hirshbein and Rahman Letters of Support).

All patients admitted to the UM and HF inpatient psychiatry units will be initially screened for the intervention via review of their electronic medical records and consultation with the patient's attending psychiatrist. Those who meet initial study inclusion/exclusion criteria will then be approached by the research assistant (RA) to describe the study and complete the remaining screening items.

Enrollment. Based on estimates from the participating sites and pilot study results, we anticipate 480 (60% of 800) patients will be admitted to UM and 720 will be admitted to HF (60% of 1200) per year with medical record documentation of suicidal ideation or a suicide attempt. Of these, we estimate 60% will not meet eligibility criteria and 22% will refuse, such that 216 could be recruited per year. Enrollment of 490 participants during 3 years of recruitment is therefore feasible. The PI of the study (Paul Pfeiffer) will oversee recruitment via weekly calls with HF site PI (Brian Ahmedani) and coordinators from both sites. Informed consent will be obtained from all participants (see Protection of Human Subjects). Baseline measures (see D2j) will be completed at the time of enrollment, prior to study arm assignment.

OUTCOMES

Primary Outcome

The primary outcome is to determine the effectiveness of the PREVAIL peer mentorship intervention for reducing suicide attempts and suicidal ideation among recently hospitalized adult psychiatric patients at high risk for suicide. In a two-site, randomized controlled trial (N=490), participants in the 3-month peer mentorship intervention arm will be less likely to report a suicide attempt on the Columbia Suicide Severity Rating Scale (CSSR-S) and will report less severe suicidal ideation on the Beck Scale for Suicidal Ideation (BSI) over the 6 months following enrollment compared to those in an enhanced usual care control condition.

1. Suicide attempts (measured by the Columbia Suicide Severity Rating Scale) [Time Frame: 6-months]. Any suicide attempt as measured according to an electronic self-report version of the Columbia Suicide Severity Rating Scale (CSSR-S). The definition of suicide attempt for the primary outcome will consist of any actual suicide attempt, aborted suicide attempt, or interrupted suicide attempt according to the CSSR-S.

2. Change in suicidal ideation (measured by the Beck Suicide Scale (BSS)) [Time Frame: Baseline, 6-months]. Patient's current suicidal ideation as measured by the Beck Suicide Scale (BSS). The BSS is a self-report 19-item scale preceded by five screening items. The BSS and its screening items assess thoughts, plans and intent to commit suicide. All 24 items are rated on a three-point scale (0 to 2). Total scores could range from 0 to 38 (if the screening items are included). No specific cut-off scores exist to classify severity. Increasing scores reflect greater suicide risk.

Secondary Outcomes

1. Medically serious suicide attempts (measured by the medical record) [Time Frame: 6-months]. Any suicide attempt as notated in the patients electronic medical record. The definition of suicide attempt for the primary outcome will consist of any actual

suicide attempt, aborted suicide attempt, or interrupted suicide attempt.

2. Self efficacy to avoid suicidal action (measured by the Self Efficacy to Avoid Suicidal Action Scale) [Time Frame: Baseline, 6 months]. Patient's self efficacy to avoid suicidal action, as measured by the Self Efficacy to Avoid Suicidal Action Scale (SEASA). Lower scores on the SEASA were found to be associated with a higher incidence and greater severity of suicide attempts (117).

Other Pre-Specified Outcomes

Exploratory analysis on potential mediators will also measure the effects of PREVAIL on the following outcomes, and the potential role of these factors as mediators:

1. Change in hopelessness, as measured by the Beck Hopelessness Scale (BHS), a 20-item measure of the degree of pessimism and negativity about the future. Summed scores range from 0 to 20. Scores provide a measure of the severity of self-reported hopelessness: 0-3 minimal, 4-8 mild, 9-14 moderate, and 15-20 severe. (66)
2. Change in quality of life, as measured by the Quality of Life Enjoyment and Satisfaction Questionnaire Short Form (Q-LES-Q-SF), a 14-item measure of satisfaction with a variety of life domains such as physical health, work, and social relationships. The scoring of the Q-LES-Q-SF involves summing only the first 14 items to yield a raw total score. The last two items are not included in the total score but are standalone items. The raw total score ranges from 14 to 70. Higher level of enjoyment and satisfaction with life are reflected in higher scores. (96)
3. Change in functional status, as measured by the Short Form 12 (SF-12), which covers domains including: (1) physical functioning; (2) role-physical; (3) bodily pain; (4) general health; (5) vitality; (6) social functioning; (7) role emotional; and (8) mental health. Summary scores are calculated by summing factor-weighted scores across all 8 subscales, with factor weights derived from a US-based general population sample. Physical and Mental Health Composite Scores (PCS & MCS) are computed using the scores of twelve questions and range from 0 to 100, where a zero score indicates the lowest level of health measured by the scales and 100 indicates the highest level of health. (100)
4. Change in perceived social support, as measured by the Multidimensional Scale of Perceived Social Support (MDPSS), which contains 12-item Likert scale items with three subscales to address different sources of support: family, friends, and significant other (118)
5. Change in depression, as measured by the Patient Health Questionnaire (PHQ-9), a measure of the severity of depression. (103, 104)
6. Change in hope, as measured by the State Hope Scale, a 6-item measure that contains two sub-scales reflecting respondents' personal capacity for change (agency) and knowledge regarding how to achieve change (pathways) (114).
7. Change in burdensomeness, as measured by the burdensomeness subscale of the Interpersonal Needs Questionnaire (INQ), a measure of the interpersonal theory of suicide construct of burdensomeness.
8. Changes in perceived emotional support, as measured by the 8-item emotional support scale of the NIH Toolbox Adult Social Relationship Scales (ASRS) (65)
9. Changes in perceived instrumental support, as measured by the 8-item instrumental support scale of the NIH Toolbox Adult Social Relationship Scales (ASRS) (65)
10. Changes in perceived friendship, as measured by the 8-item friendship scale of the NIH Toolbox Adult Social Relationship Scales (ASRS) (65)
11. Changes in loneliness, as measured by the 5-item loneliness scale of the NIH Toolbox Adult Social Relationship Scales (ASRS) (65)
12. Changes in perceived rejection, as measured by the 8-item perceived rejection scale of the NIH Toolbox Adult Social Relationship Scales (ASRS) (65)
13. Self-reported utilization of health care services, as measured by the adapted Health Services Inventory (87).
14. Medication adherence, as measured by the Single-Item Self Rating (SISR) Scale for Medication Adherence
15. Change in perceived meaning in life, as measured by the Meaning in Life Questionnaire (MLQ), a 10-item measure of the presence of and search for meaning in life (115).

STUDY INTERVENTIONS

Delivery and Duration. The peer mentorship intervention will be delivered by peer specialists trained and certified by the State of Michigan. Michigan is a leader in the training and deployment of peer specialists with over 1,600 peer specialists currently trained. The State training consists of 24 hours of didactic and group-based learning sessions covering role definitions and boundaries, effective communication, combating negative self-talk, problem solving, and supporting recovery through the use of the peer specialist's own recovery story and other recovery dialogues. The study will hire certified peer specialists with at least one year of professional experience providing peer support to individuals with mental health conditions. Peer specialists will also be required to be in stable recovery (i.e., continuous employment with no psychiatric hospitalization in the past year) and must be capable of speaking to a lived experience with suicidal thoughts or behaviors.

Peer specialists will be hired for the study from the pool of those already trained in the intervention during the R34, and new peer specialists will be recruited with the assistance of the director of peer specialist training in the State of Michigan, Pam Werner, who maintains a database of all state-trained peer specialists. Ms. Werner served on the expert panel that developed the PREVAIL intervention, assisted with hiring during the R34, and will continue to support the project throughout the R01 (see Letter of Support). Ms. Werner will facilitate recruitment of peer specialists for the study via direct personal contacts and e-mail announcements to peer specialists who reside in or near Washtenaw and Oakland Counties. In order to allow participants the opportunity to choose from among several PREVAIL peer mentors, we will hire 6 to 8 peer specialists with diverse backgrounds to deliver the intervention on a part-time hourly basis. We do not anticipate difficulty hiring peer specialists based on our experience during the R34 and with other peer support research trials.

Concomitant Treatment. Contamination of the EUC group would most likely occur via receipt of non-study sources of peer support. We will exclude participants who are currently receiving or intending to receive peer support outside of the study and will measure non-study peer support via the adapted Health Services Inventory measure. (87) Indirect contamination could occur via communication between PREVAIL and EUC participants while on the inpatient unit, though any such occurrences are expected to be of minor significance relative to the subsequent greater dose of peer mentorship received by PREVAIL participants.

Allowed Interventions. Once a participant is enrolled into the study, there is no restriction on the types of other care they may receive during their participation. If a participant is assigned to the peer mentorship intervention arm, study staff will send a letter to the participant's outpatient mental health provider informing them that the patient will be receiving peer mentorship as part of this study.

Prohibited Interventions. Participants are not prohibited from receiving any other type of intervention once they are enrolled in this study. Participants are informed during the enrollment visit that participation in more than one study at the same time may increase risks to them, and encouraged to gain approval from researchers before taking part in more than one study.

Lifestyle Considerations. Participants are not required to alter any lifestyle behavior or activities while participating in this study.

Intervention Discontinuation. Researchers may discontinue a participant's involvement in the PREVAIL intervention if it is not in the participant's best interest to remain in the study. If a participant informs the researchers that they wish to stop participating in intervention activities, the researchers will inform the participant that they may still complete the 3 and 6 month outcome assessments. Every effort will be made to retain participants who do not complete the prescribed research intervention in order to collect outcome measures from them.

Treatment Fidelity. Peer mentors will attend a weekly group supervision meeting (one for each recruitment site) led by a psychiatrist or clinical psychologist to review each active participant. The meeting allows clinicians to provide guidance regarding difficulties the peer mentors may encounter that are beyond their training or scope. The meetings also reinforce fidelity to the intervention by reviewing training materials, planning future sessions, or reviewing audio-recorded portions of prior sessions to provide feedback on the use of ILSM conversations and general support skills. The group format also allows peer mentors to receive support from other mentors. Fidelity to the PREVAIL intervention will also be monitored through formal fidelity ratings of a random sample of 20% of each peer mentor's audio-recorded sessions utilizing the fidelity rating scale developed during the pilot study (see C1c). Peer mentors who deliver a session without adequate fidelity will receive individual supervision and more frequent monitoring.

Suicide risk is managed by peer mentors via a structured protocol (Other Attachment IIA, page 39-40). At each session, peers ask participants if they are thinking about suicide or have made a recent suicide attempt. If endorsed, additional questions regarding plans and intent are asked. Depending on the participant's responses, the peer mentor will immediately contact a study clinical supervisor (psychiatrist or clinical psychologist) who may speak with the participant to determine the appropriate disposition (e.g., activate emergency medical services) or provide further instructions. The participant's mental health clinician(s) will also be notified of an increase in suicide risk. This protocol was successfully used during the R34 pilot study, and in two instances participants were referred to emergency medical services based on acute risk.

Overall Compliance. A participant will be done with the intervention when they have completed the last session with the peer specialist, which will typically occur within one week of the 3-month outcome assessment.

Treatment Design. During the intervention development phase of the R34 project, an expert panel including suicide prevention researchers (King, Ilgen, Holloway), peer support researchers (Valenstein, Chinman), peer specialists (Coe, Derosa), the peer support training director for the State of Michigan (Werner), and an inpatient psychiatry unit director (Hirshbein) were convened to create an outline of the essential training needs for peer mentors to work with high-risk patients and address suicide risk according to the interpersonal theory of suicide. The outline of the training and intervention content was then used as the basis for developing the training manual (Other Attachment IIA). This manual was developed by the PI (Pfeiffer) in conjunction with Eduardo Vega and Dequincy Jones of the Center for Dignity, Recovery, and Empowerment (a peer support advocacy, service, and research organization); Kristen Abraham,

a clinical psychologist with expertise in peer support; and Debra Levine, a clinical psychologist and post-doctoral fellow. Drafts of the training materials were circulated among the expert panel and revised in an iterative process.

Interventionist Training. The PREVAIL intervention training will be delivered to new hires over 3 days according to the intervention training manual (Other Attachment IIA). The training will be modified slightly from the R34 training in that the ILSM conversations (Other Attachment IIB) will be integrated within appropriate modules, and the "PAUSE" communication skills module will removed due to poor fit among the peer mentors during the R34. The first day of the PREVAIL training is intended to review the basic skills of peer support from the perspective of working with individuals at high risk for suicide. This includes a focus on listening and validation skills. The second day focuses on specific techniques for addressing hopelessness and belongingness. This includes sharing of one's own recovery story as it relates to suicide, developing physical reminders of hope (e.g., a hope kit), setting hopeful goals, increasing the support one receives from others, and coping with loss. The third day focuses on skills related to motivational interviewing and addressing suicidal crises (e.g., reinforcing safety plans, self-soothing coping skills, and escalating management to a clinical supervisor). The training also includes modules on prevention of vicarious trauma among peer mentors, working with individuals who are difficult to engage, and guidelines for tailoring session content to the individual. The training includes a mix of small group discussion, role plays, and exemplar video vignettes.

Delivery of Treatment

Participants randomized to the peer mentorship intervention will be given a short written description of each of the peer mentors based on who is currently available to accept additional participants. These descriptions are written in the peer mentor's own voice and generally include some demographic information, description of his or her prior mental health challenges, and philosophy of peer support. These descriptions are intended to improve fit between participant and peer mentor and provide the participant with agency in establishing the relationship, as one would have with a natural support.

The peer mentor selected by the participant will then meet with the participant the next day while the participant is still in the hospital. This first session is intended to break the ice, establish "peerness" by the peer mentor briefly sharing some of his or her personal experience with mental health challenges and/or suicidal thinking or behaviors, and discuss how future sessions will proceed and the limits of confidentiality, particularly in the case of acute suicide risk.

Subsequent sessions will be scheduled according to the participant's preferences, with a suggested maximum frequency of twice weekly for the first two weeks, weekly for weeks 3 to 8, and then every other week for the last month. In the R34 trial, participants' preferences and availability for contact varied widely, though several participants who completed only a few mentorship sessions still expressed appreciation of the visits during semi-structured qualitative assessments at 3 months. Allowable meeting locations include public places in the community (e.g., coffee shop, park), the participant's home, or a research clinic space. Meetings can also be by phone, which comprised 26% of sessions in the R34.

Preference for the 3-month timeframe was assessed and confirmed by peer mentors and participants during the R34. In order to minimize possible distress that participants may experience at the loss of support at 3 months, and to protect participants to whom an abrupt end to the peer relationship may be harmful, peer mentors may offer participants the option of 3 additional sessions over 4 weeks following the end of the 3-month intervention. These additional sessions will take place over the phone.

Session content is flexible and allows for the peer mentor to provide general supportive listening, validation, and sharing. Session duration is on average 1 hour with at least 15 minutes discussing hope or belongingness according to the ILSM conversation guides. The choice of conversation is determined by the peer mentor based on the participant's needs with input during group supervision. The session structure and content are intentionally highly flexible to allow for genuineness in the peer relationship, thereby increasing acceptability and implicit belongingness. One exception to this flexibility is that during the first session after hospital discharge, peer mentors are instructed to review the participant's suicide safety plan that was created during their inpatient stay and revise as necessary. The 6-week session is also a "check in" point for the peer mentor to gauge preferences for more- or less-structured conversations or sharing and remind participants they are half-way through the intervention so they anticipate termination at 3 months. Without this reminder, some participants were distressed (e.g., tearful, but not suicidal) at the loss of support at 3 months during the pilot.

Enhanced Usual Care Condition. An enhanced usual care (EUC) control condition was chosen: 1) to address ethical concerns that high-risk study participants should receive some enhancement in care, 2) to address concerns that comparing peer mentorship to no enhancement in care lacks equipoise, 3) to increase expectancy and credibility among control participants, and 4) to increase external validity by demonstrating the intervention is superior to another credible health system approach to suicide prevention.(82) The EUC condition will consist of a "caring message" from the study team via e-mail or text message (based on the participant's preference) 24-72 hours after discharge. An example message is, "We hope things are going well for you since you left the hospital. If you wish to reply, we'd be glad to hear from you". A list of local mental health resources will be available if participants reply and during the 3 and 6-month follow-up assessments.

The EUC condition is modeled on prior studies of caring letters and brief contacts by health professionals after suicidal crisis and

national recommendations to provide post-crisis follow-up contacts.(31, 83-86) Responses will be addressed by a research assistant trained in suicide risk protocols; after-hours responses will be automatically sent crisis resources. Participants randomized to the peer mentorship arm will also receive EUC.

A control condition consisting of contacts with a peer specialist without PREVAIL training was not chosen due to ethical concerns that well-intended but poorly trained individuals may provide harmful messages to suicidal individuals ("suicide is selfish", "suicide is an easy way out") or fail to detect or manage acute risk. An attention-only control group using trained professionals would diminish the relative effect of peer mentorship (attention is a nonspecific but active component of the intervention) and would require an increase in sample size that would greatly increase the complexity and cost of the trial. We decided it would be a more judicious and pragmatic use of resources to first test the effectiveness of PREVAIL compared to EUC, and consider future comparative effectiveness trials if effectiveness is demonstrated in this initial trial. We note we will measure the hypothesized mechanisms of peer mentorship and, if effective, future studies could explore differences between peer mentorship and non-specific attention on these intermediate outcomes.

SAFETY

Potential Risks. A potential risk to study participants is clinical deterioration of their psychiatric condition and/or an increase in suicidal thoughts or behaviors related to receiving the peer mentorship intervention. This risk is anticipated to be minimal based on the conduct of the R34 pilot study, where suicidal ideation improved for the whole population from baseline to 3 months according to the Beck Scale for Suicidal Ideation, and no adverse events were attributed to study participation. Additionally results of a prior RCT showed peer mentorship resulted in fewer psychiatric hospital readmissions (Sledge et. al., 2011); another large RCT demonstrated that peer mentorship was effective for preventing worsening depression (Dennis et. al., 2009). An additional RCT of mutual peer support for depression conducted by study team members (Valenstein et. al., 2015) showed no indication of clinical deterioration or increased suicidal ideation from participation in the intervention.

Another potential risk to study participants is loss of confidentiality related to self-report assessment data, medical record data, or audio recorded sessions or interviews. The risk of an inadvertent breach of confidentiality is minimal (see below regarding protections against risk). Because of the nature of the study (i.e., peer mentorship to reduce future suicide risk), it is necessary to obtain detailed information about past and current suicidal thoughts, plans and behaviors. Loss of confidentiality may also occur in the event that the participant is assessed by a study clinician to be high risk for suicide and a breach of confidentiality is conducted to ensure the participants safety (e.g., contacting a participant's family member or health care provider). Such a breach would only occur if there is no reasonably safe alternative. The informed consent process and documents will explain mandatory reporting requirements for information regarding intention to harm self or others (e.g., suicide, homicide) prior to participating in the study.

There is also a minimal risk of psychological discomfort to study participants from the questions asked in the assessments. Participants may become anxious or uncomfortable as a result of being asked personal questions. The study team members conducting assessments are trained to respond to this emotional distress and to refer the participant to their mental health provider or other appropriate resources as necessary. All participants are free to terminate the assessments at any time or refuse to respond to any questionnaire item. Additionally, there is a popular misperception that enquiring about suicidal thoughts, plans or prior behaviors could increase the likelihood that an individual will make a suicide attempt. Research has not found that suicide assessments increase the risk of suicidal behaviors and this misperception has had the unfortunate consequence of decreasing research on potentially suicidal individuals (see review in Pearson et al., 2001). However, the study of individuals at elevated risk for suicide does require a clear set of procedures to manage potential crisis situations. These are reviewed in detail in the Data Safety and Monitoring plan.

Potential Benefits. It is believed that research participants may be helped in a number of ways. Participants who take part in the peer mentorship arm may benefit from an improved sense of hope and belongingness as the peer mentor establishes a supportive relationship and role-models recovery. The peer mentors are trained and supervised to handle potential distress caused by participating in the intervention conditions. The peer mentors will have immediate "on call" access to a supervising mental health clinician at all times they are meeting with a participant. The peer mentorship condition includes standard care treatment through the University of Michigan and Henry Ford Kingswood inpatient psychiatry units, including assessments and referrals if deemed appropriate by the clinical staff. All participants in the control condition will receive the same standard of care. In addition, participants will receive a caring contact 24 to 72 hours after discharge and written referral information regarding services and community resources for individuals experiencing suicidal thoughts, plans or related psychiatric problems. The potential benefits for the research are expected to outweigh the risks to participants.

Assessment of Potential Risks & Benefits. After years of steady increases, in 2014 the suicide rate in the U.S. reached its highest rate in decades with over 42,000 suicide deaths in that year. Suicide has surpassed motor vehicle accidents as a leading cause of death in the U.S., is the 10th leading cause of death overall, and is in the top 4 leading causes of death for those younger than 65. For every suicide death, approximately 25 times as many people (over 1 million Americans) attempt suicide, resulting in emergency department visits or hospitalizations costing the U.S. $10.4 billion dollars in health care expenditures and lost productivity. The costs of suicide extend to include negative health and social functioning effects on surviving loved ones. Preventing suicide is a priority across a range of US federal health departments, and new approaches to suicide prevention are critically needed. Randomized controlled trials have shown

that peer support interventions are effective in reducing symptoms of depression, reducing the likelihood of psychiatric hospital readmission, increasing hopefulness, and improving quality of life. However, there are few data on the effectiveness of these approaches in suicide prevention. Developing an effective intervention for preventing suicidal behaviors would represent a major advancement given the few existing effective interventions to date. This study will add to the knowledge base in this critical area. Given the potential for this study to improve the quality of life for patients with varying levels of access to evidence-based mental health services, the potential benefits outweigh the risks outlined above.

Event	Relation to study	Reporting Timeframe
Suicide death	Reported as "unlikely related" at minimum.	Individually within 7 days of study team discovery.
Medically serious suicide attempt	Reported as "unlikely related" at minimum.	Individually within 7 days of study team discovery.
Psychiatric hospitalizations or emergency room visits due to suicidal intent or behavior	Unrelated	Not reported per standard reporting guidelines.
	Related	Reported individually within 14 calendar days of study

<u>Event Reporting Schedule</u>

Any adverse events not listed as "expected" below will be reported to the UM or Henry Ford Hospital IRB per standard reporting guidelines when relevant, and to the DSMB and the Project Officer for this grant. The person responsible for reporting adverse events will be Dr. Pfeiffer. The timing of the reporting of any adverse event to the IRB and DSMB by Dr. Pfeiffer will be dependent on the severity of the event. For all *related* adverse events not listed as "expected" below, we will adhere to the standard reporting guidelines regarding the timing of report. More specifically, life-threatening adverse events will be reported as soon as possible but within 7 calendar days; non-threatening potentially serious adverse events that are causally related to the research will be reported in writing within 14 calendar days, and non-serious adverse events will be reported in aggregate form with annual reviews.

<u>Expected Adverse and Serious Adverse Events</u>

Given that this study is recruiting from inpatient psychiatric units, and that the eligibility criteria include suicidal ideation immediately prior to current inpatient psychiatric admission, there are several serious and non-serious adverse events that are expected, not as result of the study activities, but rather as a result of the characteristics of the study population. The following adverse events are considered expected as a result of the characteristics of the study population:

1. Suicide-related death
2. Medically serious suicide attempts (e.g., requiring *medical* hospitalization)
3. Psychiatric hospitalizations or emergency room visits due to suicidal intent or behavior
4. Suicide attempts not requiring medical hospitalization
5. Breach of confidentiality associated with reporting suicidality to agency staff, appropriate authorities, and/or mental health personnel.
6. Suicidal thoughts

<u>Reporting Guidelines</u>

Expected adverse events and reporting plan:

		team discovery per standard reporting guidelines.
Suicide attempts not requiring medical hospitalization	Unrelated	Not reported per standard reporting guidelines.
	Related	Reported individually within 14 calendar days of study team discovery per standard reporting guidelines.
Breach of confidentiality associated with reporting suicidality to agency staff, appropriate authorities, and/or mental health personnel.	Unrelated or Related	Not reported per standard reporting guidelines.
Suicidal thought	Unrelated or Related	Not reported per standard reporting guidelines.

Note: Adverse events ***not*** *listed above will be reported using the standard reporting guidelines and timetable*

Though these adverse events are considered expected for the study population; determining that an event is “unrelated” to study activities requires comprehensive evaluation and tracking for the population of this study. In order to ensure patient safety and monitor any disparity between the intervention and enhanced usual care groups, a study specific reporting plan will be used for the two most serious expected adverse event categories. Suicide deaths and medically serious suicide attempts will be reported to the IRB as “unlikely related” at minimum, as potential changes in trajectory due to study participation should be monitored for both intervention groups. A brief records review will be completed for suicides and hospitalizations in order to confirm that there is no mention of study participation as a causal factor. Please see below for reporting timetable and guideline detail. These events will be reported to the DSMB bi-annually, unless frequency increases beyond that which is expected for the population. Of note: If there is evidence that an adverse event is “related” to study participation, it will be reported individually within 14 calendar days of study team discovery per standard reporting guidelines.

Safety Monitoring
The Principal Investigator, Dr. Pfeiffer, ultimately will be responsible for monitoring the data and safety with involvement from all of the study investigators. It should be noted that all research projects involving human participants at the University of Michigan require approval from the University of Michigan Medical School’s Institutional Review Board (IRB); IRB approval will also be obtained from the Henry Ford Health System IRB. In addition, because of the sensitive nature of the data being collected, a Certificate of Confidentiality will be obtained for this study from the National Institute on Health (NIH).

Dr. Pfeiffer will ensure that all relevant IRB policies, procedures and stipulations are being followed. Dr. Pfeiffer also will be responsible for ensuring that other investigators and project staff adhere to the UM IRB policies including: (1) all participants will understand, agree to and sign a written consent form before participating; (2) strict adherence to a participant’s right to withdraw or refuse to answer questions will be maintained; (3) the assessments will be completely confidential and no names will be associated with the assessment data; (4) consent forms and identifying information will be kept separate from the actual participant data; (5) all identifying information (consents, tracking data) will be kept locked at all times and computer files will be saved with passwords; (6) participants will be informed in writing in the consent form how to contact the PI, the study coordinator, and UM IRB office with any questions and/or concerns.

Dr. Pfeiffer will directly supervise the data manager and the project coordinator and will be responsible for monitoring confidentiality procedures. Quality control and reliability of screening, baseline and follow-up assessments will be monitored by Dr. Pfeiffer throughout the trial via regular meetings and observation of the project coordinator conducting standardized assessments. Dr. Kim, the study biostatistician, will monitor the quality of the data files via supervision of the data manager.

A Data Safety and Monitoring Board will be created for this project. The DSMB will be composed of three faculty not involved with the project, who have expertise in randomized controlled trials, psychiatric emergencies, and statistical analysis. The DSMB will review the protocol before the study is initiated, with an emphasis on participant safety, and can recommend changes. Once the study begins, the committee will meet bi-annually throughout the study to review data on adverse events, recruitment, and adherence to the protocol. For more information on the DSMB and monitoring procedures, see *Section: Monitoring.*

Definitions for Adverse Events and Serious Adverse Events

- **Adverse Event (AE)** is any experience or abnormal finding that has taken place during the course of a research project and was harmful to the subject participating in the research, or increased the risks of harm from the research, or had an unfavorable impact on the risk/benefit ratio.
- **Serious Adverse Event (SAE)** is any adverse experience occurring at any dose or level of participation that results in any of the following outcomes: death, a life-threatening experience, hospitalization or prolongation or existing hospitalization, a persistent or significant disability or capacity, or a congenital anomaly or birth defect.

STATISTICAL ANALYSES PLAN

Sample Size. Based on our pilot study findings and those of other randomized controlled trials of psychosocial interventions that included suicide attempts as a primary outcome (91, 92, 109), we powered the study with the assumption that frequency of suicide attempts at 6 months will be 22% in the intervention arm vs. 36% in the control arm. We will enroll 490 patients in total, and assuming a 25% dropout rate, 368 (184 per group) will provide primary outcome data. The proposed sample size is expected to provide 84% power to detect the desired difference between the two groups based on a two-sided 0.05 level test. The estimate of effect is based on an assumption that the effect of PREVAIL will be similar to that reported in a trial of cognitive behavioral therapy for suicide among suicide attempters (N=120) where the re-attempt rate at 6 months was 25% among intervention recipients vs. 40% for controls. (91) Although there is no prior literature comparing the effects of peer support to psychotherapy on suicide-specific outcomes, two meta-analyses have concluded the effects of peer support on depression are similar to psychotherapy. (49, 51) Our estimate of effect is conservatively less than that found for brief psychodynamic interpersonal therapy, where in one trial (N=119) the self-harm rate at 6 months was 9% in the intervention arm compared to 28% for controls. (92) For the primary outcome of suicidal ideation according to the Beck Scale for Suicidal Ideation at 6 months and measures of hope and belongingness at 3 months (Aim 2), our sample of 368 will provide 90% power to detect a standardized effect size (Cohen's d) of .34. For the mediator analysis, the proposed sample size is expected to provide 80% power to detect a significant mediator assuming that the intervention explains about 2% of the variance of the mediator (corresponding to "small" effect based on Cohen's criteria), and about 7% of the variance of suicide ideation is explained by the mediator adjusting for the intervention effect, or vice versa based on joint significance testing for the mediation effect. (110)

Data Analysis

Baseline Analyses: We will examine the randomization process to determine if there were any between-group differences at baseline, particularly in variables that are thought to be potentially associated with suicide attempts or ideation. T-tests or chi-square analyses, as appropriate, will be used to test for differences in the following baseline variables between study groups: age, severity of suicidal ideation prior to admission, burdensomeness, hope/hopelessness, and measures of belongingness. When there are significant differences in the distribution of these variables, they will be included as covariates in multivariable analyses. Balance is expected for sex and recent suicide attempt due to randomization by minimization.

Data Verification/Univariate Analyses: We will examine the distribution of all study variables for extreme values, missing data, variances, possible coding errors, skewness and whether or how to categorize skewed data. We will describe means (SD) for the continuous measures and frequencies of dichotomous outcomes by study group for each assessment time for the entire sample and for each of the study sites, separately.

Primary Outcome

Primary analyses will determine the effectiveness of PREVAIL in decreasing the risk of any suicide attempt and in decreasing suicidal ideation at 6 months post-randomization. Primary analytic cohort will be "intent to treat", such that all randomized participants will be included according to their original study arm assignment, regardless of whether they subsequently drop out of participation in the intervention. Secondary analyses will be conducted using a "per protocol" cohort where only participants who complete 6 or more peer encounters will be compared to those assigned to the EUC arm. We will evaluate predictors of compliance by defining 6 or more peer encounters as compliance and will also obtain complier average difference (a.k.a. complier average causal effect) assuming a balanced proportion of compliance between arms. The estimates of the three analytic cohorts (intent to treat, per protocol and compliance average difference) will provide more robust understanding of the effect of PREVAIL. (106)

Bivariate/Multivariate Analyses*:* To test for intervention effects at 6 months on the primary outcomes, a logistic regression model will be used for the dichotomous outcome of suicide attempt (as assessed by CSSR-S) and a multiple regression model will be used for suicidal ideation (as assessed by BSI). Each model will include the study arm as the primary predictor and will also include study site, gender, and prior suicide attempt, which were the balancing variables used in randomization. We will also adjust for additional covariates that show imbalances in baseline analyses. Outcomes at 6 months post-randomization will be our primary analysis in order to assess whether the intervention has lasting effects, but we will also assess immediate effects at 3 months using similar analytic approaches.

Effects over time: We will also assess the intervention effects over time using longitudinal data for both suicidal ideation and suicide attempt. For suicidal ideation, we will use a linear mixed-effects model with ideation assessed at baseline, 3 months, and 6 months. The model will allow the full use of all observed data despite missing outcomes at one or two assessment times and will give unbiased estimates as long as the missingness does not depend on the unobserved missing data (missing at random). For suicide attempt, we will use a generalized linear mixed-effects model with logit link for any suicide attempt in the prior 3 months as a binary response variable assessed at 3 and 6 months. These models will include

participant as random intercepts to take into account the correlation of data within person over time. The models will allow estimation of the time-averaged effect of the intervention if the response to the intervention is immediate and lasting, or estimate the differential effect of the intervention over time if a significant interaction between time and intervention is found, such as different trends between the study groups. Whether we compare the time- averaged effects or the rates of changes between two groups, we will be guided by careful graphical exploration of the longitudinal data over time between the two groups. For example, if we find decreasing BSI scores from baseline to 3 months in both groups, but with a larger decrease in the intervention group, this will be modeled with interaction terms of time indicators for 3 and 6 months each by intervention group. The coefficients from the interaction terms of the BSI model will then allow testing for the between-arm difference in decrease in BSI from baseline to 3 months and baseline to 6 months. Similarly, the interaction term of the suicide attempt model will allow testing if the odds ratio of suicide attempt associated with the intervention differs from month 3 to month 6.

Secondary Outcomes

The effects of the intervention on the primary hypothesized mediators of hope (Hope Scale and Beck Hopelessness Scale) and belongingness (NIH Toolbox measures) will be assessed using multiple regression models based on a similar model as described under aim 1 for suicide ideation. Primary interest will be comparisons between treatment arms at 3 months (primary analysis) and 6 months (secondary analysis). Similar exploratory analyses will be used to assess intervention effects at 3 and 6 months on functioning, quality of life, depression, patient activation, healthcare utilization, and medication adherence.

Figure 4. Mediation Model of Intervention Effectiveness

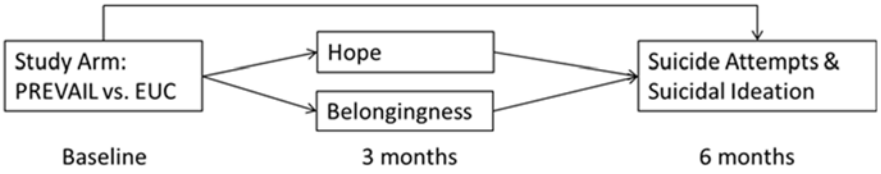

Mediation of Intervention Effects on Suicide Attempts and Suicidal Ideation: We will examine whether any improvements in the primary outcomes of suicide attempts or suicidal ideation are mediated through improvements in hope and belongingness in primary exploratory analyses. The role of functioning, quality of life, depression, patient activation, healthcare utilization and medication adherence as mediators will be assessed in secondary exploratory analyses. To test whether a variable might be a mediator, we will first test the associations between the intervention arm and the mediator (described in above bivariate analyses, section 8.2.2), and then the mediator and the outcome of interest using bivariate analyses (e.g., hope at 3 months and suicidal ideation at 6 month). A variable will be considered a potential mediator if the variable at the 3-month assessment is associated with the intervention arm and with the outcome at 6 months as determined by a significance level of $p<.10$.

Potential mediators (at 3 months) will then be included as covariates in multivariate regression models predicting the primary outcomes at 6 months (Figure 4). If, as hypothesized, the intervention's effects on the primary outcomes are mediated through hope and belongingness (or other exploratory mediators), the variance in the 6-month outcomes explained by the intervention group will be less once the mediator variables are included in the models. Changes from baseline to 3 months will also be examined as potential mediators.

The intervention effects on any and number of readmissions to a psychiatric inpatient unit, emergency department visits, and outpatient mental health treatment visits (e.g., medication prescriber or psychotherapist) will be analyzed using generalized linear models with appropriate link function.

Approach to Missing Data: Our initial analyses will only use observed data. When data are missing for items within scales, we will use recommended imputation procedures rather than excluding participants from the analyses. For missing follow-up assessments, we will check if missingness is associated with any baseline variables including sites, and will include any such baseline variables in the models evaluating the intervention effect described above. In addition, as described under Effect Over time, the mixed-effects model is expected to provide an unbiased estimate of the intervention effect if missingness is at random. As a secondary analysis, we will also conduct an analysis that imputes missing data. We will likely have some missing baseline values in addition to missing follow-up assessments. Thus we will conduct multiple imputations based on chained equations (sequential regression multivariate imputation) to impute both baseline and follow-up data and will summarize the data using appropriate combination rules. (107, 108)

REGULATORY, ETHICAL AND STUDY OVERSIGHT CONSIDERATIONS

Informed Consent Process

Patients who appear to meet study inclusion and exclusion criteria based on review of medical records and consultation with the attending psychiatrist will be approached by the research assistant (RA) to describe the study, provide information about the randomization and 3 and 6-month assessments, as well as the risks, benefits, and limitations to confidentiality. Patients who state they are interested will be asked to complete the Mini-Cog to assess whether they are substantially cognitively impaired and therefore unable to

give informed consent. Patients who are not substantially cognitively impaired (based on the Mini-Cog) will complete a pre-screening consent form allowing study staff to administer the Beck Suicide Scale and items 4 and 5 of the Columbia Suicide Scale. Patents who have a score >5 on the Beck Suicide Scale will be eligible to participate and obtain informed consent. During the informed consent process, the study will be described and patients will have ample and repeated opportunities to ask questions.

We are requesting a waiver of consent for recruitment purposes. We will be screening medical records to determine patient eligibility. Due to the large number of patients admitted to the inpatient psychiatric unit, it would be difficult to obtain consent from each patient prior to screening. The risk to patients is minimal in order to allow staff to pre-screen medical records. Patients who are interested in participating will provide informed consent before study activities begin.

Confidentiality and Privacy. Every effort will be made to ensure that study data are always confidential, and never stored so that data can be linked to a particular person. Confidentiality will be explained to participants, including assurances that law enforcement personnel, and any others outside the research staff will not be informed of their responses. Limits to confidentiality will be explained verbally and in writing (e.g., physical or sexual abuse of minors, imminent threat to harm self or others).

Future Use of Stored Data. Data will be retained for study recordkeeping purposes until the end of analysis period and at least seven years from the end of the study period, in keeping with the relevant record-keeping guideline. There will not be any change in the conditions or arrangements for storage of research data/specimens during the retention period.

Data Handling & Recordkeeping

Data collection is the responsibility of the clinical trial staff at the site under the supervision of the site investigator. The investigator is responsible for ensuring the accuracy, completeness, legibility, and timeliness of the data reported. All source documents should be completed in a neat, legible manner to ensure accurate interpretation of data. Preliminary eligibility data will be collected from participant's electronic medical records and entered into a 21 CFR Part 11-compliant Microsoft Access database provided by the University of Michigan. Data backups will be performed regularly. This data will be linked by a coded identifier to direct identifiers (name, phone number, address, and medical record number) but stored separately. Data need to be linked to subjects' identities to help facilitate follow-up data collection procedures. Identifiers will be retained in accordance with the requirements of record-keeping for regulatory purposes. Clinical data will be entered directly from the electronic medical record.

Outcome assessment data will be collected at baseline, 3 months, and 6 months using Qualtrics, an Application Server Provider (ASP) with a Software-as-a-Service (SaaS) platform for the creation and distribution of online surveys and related services. The platform records response data, and performs analysis and reporting. All services are located online. Qualtrics servers are protected by high-end firewall systems, and vulnerability scans are performed regularly. Complete penetration tests are performed yearly. All have quick fail over points with redundant hardware, and complete backups are performed nightly.

Qualtrics uses Transport Layer Security (TLS) encryption, known as SSLv3.1, for all Internet transmitted data. Surveys may be protected with passwords. Qualtrics services are hosted by trusted third party data centers that are SSAE-16 SOC 1 Type II audited. All data at rest are encrypted, and data on deprecated hard drives are destroyed by U.S. DOD methods and delivered to third-party data destruction service.

Outcome assessments may be collected using paper surveys if internet access is unavailable during the time of the assessment, or based on a participant's needs or preferences. Paper copies of outcome assessment data will be linked to direct identifiers using a coded identifier. Deidentified data will always be stored separately from identifiable data. All paper records will be stored behind lock and key in locations that are only accessible to study staff who have been granted permission to use the data.

If outcome assessments are conducted off-site, paper copies of outcome assessment data may be transported by study staff from the location of the outcome assessment to the secure storage site. Data will be transported directly back to the storage site by study staff, and will remain on our person at all times.

Quality Assurance and Quality Control. Dr. Pfeiffer will directly supervise the data manager and the project coordinator and will be responsible for monitoring confidentiality procedures. Quality control and reliability of screening, baseline and follow-up assessments will be monitored by Dr. Pfeiffer throughout the trial via regular meetings and observation of the project coordinator conducting standardized assessments. Dr. Kim, the study biostatistician, will monitor the quality of the data files via supervision of the data manager.

Training

Dr. Pfeiffer will ensure that all relevant IRB policies, procedures and stipulations are being followed. Dr. Pfeiffer also will be responsible for ensuring that other investigators and project staff adhere to the UM IRB policies including: (1) all participants will understand, agree to and sign a written consent form before participating; (2) strict adherence to a participant's right to withdraw or refuse to answer

questions will be maintained; (3) the assessments will be completely confidential and no names will be associated with the assessment data; (4) consent forms and identifying information will be kept separate from the actual participant data; (5) all identifying information (consents, tracking data) will be kept locked at all times and computer files will be saved with passwords; (6) participants will be informed in writing in the consent form how to contact the PI, the study coordinator, and UM IRB office with any questions and/or concerns.

Every effort is made to ensure that all study data are always confidential, in terms of staff training and data storage, so that data cannot be linked to a particular person. The research coordinator, RA, peer specialists, and clinicians will sign a pledge of confidentiality and will understand that violation of confidentiality is reason for dismissal. Training of staff will include information about the importance of confidentiality and techniques to maintain confidentiality of all information reported by research participants. All study staff will be required to attend University of Michigan training in human research study protections

Dr. Pfeiffer in cooperation with Co-Investigators will be responsible for providing training to all research staff working with participants with regard to procedures for managing potential clinical deterioration, suicidal crisis situations, and/or adverse events. This training will include information regarding evaluating warning signs of acute suicidal ideation, planning, or intent that could occur during contact with participants, and means of addressing such issues. Peer mentors will inquire about suicidal ideation at every encounter with participants, and participants complete self-report measures of suicidal ideation at baseline, 3-month, and 6-month assessments conducted by RAs. In the case that a participant indicates any level of suicidal ideation or recent attempt during encounters with any study staff (including peer specialists), a suicide risk management protocol will be used. The protocol utilizes an algorithm of scripted risk assessment questions and "action steps" dependent on the participant responses. Based on the level of risk determined by the algorithm, study staff may perform one or more of the following: a) recommend the participant call or be transferred to the national suicide crisis hotline, the University of Michigan's Psychiatric Emergency Services (PES), or the nearest emergency department to immediately address suicide risk and determine whether emergency medical services or police should be activated to conduct a wellness check in the patient's home or community, b) notify the participant's outpatient treatment providers regarding a non-imminent increase in suicide risk, c) notify a designated on-call study team clinician (Pfeiffer, Ahmedani, or Abraham,) when the suicide risk management algorithm has been activated, and when any questions arise regarding risk management. The on-call study clinician will be available via pager or cell phone at all times when there is potential contact between study staff (including peer specialists) and participants.

Participants will be instructed on how to contact the national suicide crisis hotline, PES, or closest emergency department if they are experiencing a suicidal crisis. Peer specialists and study team members are not to further assess or manage suicide risk beyond the established protocol. In order to avoid participants contacting peer specialists in crisis when study clinicians are not available, peer specialists will be provided with cellular phones specifically for communication with study participants, and participants will be clearly informed that these cell phones will be off when not in use, and they are not monitored at all times. The voicemail messages on these phones will have instructions on how to contact crisis services. When Peer specialists will meet with participants outside of regular business hours; clinician back up will be prearranged. Written safety procedures for conducting community-based meetings include use of public locations, during daylight hours, and mandatory call-in procedures. In our prior research, using such procedures with similar populations, no incidents where staff safety concerns became an issue occurred during follow-up assessments. All safety concerns will be reported immediately to Dr. Pfeiffer.

Protocol Deviations. A protocol deviation is any noncompliance with the clinical trial protocol, International Conference on Harmonisation Good Clinical Practice (ICH GCP), or Manual of Procedures (MOP) requirements. The noncompliance may be either on the part of the participant, the investigator, or the study site staff. As a result of deviations, corrective actions are to be developed by the site and implemented promptly. It is the responsibility of the site investigator to use continuous vigilance to identify and report major protocol deviations that may adversely impact safety of participants, or impact integrity/validity of the data, and minor protocol deviations as part of a pattern or suggesting a systemic problem in study conduct that potentially places subjects or others at a greater risk of harm than was previously known or recognized, within seven (7) calendar days of becoming aware of the event or information.

Monitoring

The Principal Investigator, Dr. Pfeiffer, will be responsible for monitoring the data safety and quality. It should be noted that all research projects involving human participants at the University of Michigan, including the proposed, require approvals from the University of Michigan Medical School's Institutional Review Board (IRBMED). In addition, because of the sensitive nature of the data being collected, a Certificate of Confidentiality will be requested.

Dr. Pfeiffer will ensure that all relevant IRBMED policies, procedures, and stipulations are being followed. They will also will be responsible for ensuring that other investigators and project staff adhere to the UM IRBMED policies including: (1) All participants will understand, agree to and sign a written consent form before participating; (2) strict adherence to a participant's right to withdraw or refuse to answer questions will be maintained; (3) study assessments will be confidential and no names will be associated with the assessment data; (4) consent forms and identifying information will be kept separate from the actual participant data; (5) all identifying information (consents, tracking data) will be kept locked in a filing cabinet at all times and computer files will be saved with passwords;

and (6) participants will be informed, in the consent form, how to contact the PI, the study coordinator, and the IRBMED office with any questions and/or concerns. Dr. Pfeiffer will be responsible for reporting any amendments to the UM IRBMED prior to implementation of any changes to the protocol.

Dr. Pfeiffer in cooperation with Co-Investigators will be responsible for providing training to all research staff working with participants with regard to procedures for managing potential clinical deterioration, suicidal crisis situations, and/or adverse events. This training will include information regarding evaluating warning signs of acute suicidal ideation, planning, or intent that could occur during contact with participants, and means of addressing such issues. All safety concerns will be reported immediately to Dr. Pfeiffer. For more information on how research staff will be trained to manage potential crisis situations and/or adverse events, see *Section 9.4.2 – Training.*

The timing of the reporting of an adverse event (unexpected events) to the UM IRBMED by Dr. Pfeiffer will be dependent on the severity of the event, and whether such adverse events were expected (included in the informed consent). All research projects involving human participants at the University of Michigan require approval from the University of Michigan Medical School's Institutional Review Board (IRBMED). SAEs will be reported to the IRBMED per the study-specific reporting guidelines outlined in section 7.4 and to the Project Officer for this grant. Dr. Pfeiffer will be responsible for reporting adverse events.

Because participants are at increased risk for suicide, we felt it was necessary to enhance usual care. All participants who enroll in the study will be offered a list of local mental health resources and social service organizations at the 3 and 6-month follow-up assessments. Participants assigned to the enhanced usual care control condition will receive a "caring message" from the study team via e-mail or text message (based on the participant's preference) 24-72 hours after discharge. An example message is, "We hope things are going well for you since you left the hospital. If you wish to reply, we'd be glad to hear from you". A list of local mental health resources will be available if participants reply. This procedure was elected as a compromise between research integrity (e.g., not wanting to unduly influence outcome in this study) and responsibility to human participants protection.

An independent Data Safety Monitoring Board will be established that will review: (a) research protocols and plans for data and safety monitoring, (b) adverse events and unforeseen outcomes, (c) ethical issues related to recruitment activities, (d) risk management policies and activities, and (e) conduct intermediary data analyses, when appropriate. The board will be approved by the PIs, sponsoring Institute, and the University of Michigan Medical School IRB. DSMB members will be responsible for defining the events that will trigger additional "as needed" reviews and the criteria for retaining or removing patients from studies if they suffer from an adverse event. In addition, the DSMB will make recommendations to the study investigators about continuation or discontinuation of the project. These responsibilities are in line with the "NIH Policy for Data and Safety Monitoring" and further Guidance on Data and Safety Monitoring for Phase I and Phase II Trials." The DSMB members include scientists and clinicians with expertise in randomized controlled trials, psychiatric emergencies, and statistical analysis. The DSMB will meet in year 1 prior to the enrollment of the first participant and then every 6 months until the trial concludes, and will be informed promptly of all serious adverse events. The DSMB will meet "as-needed" to discuss new study protocols and adverse events occurring between scheduled meetings.

The DSMB members are: Rachel Glick, Lisa Brenner, and Timothy Hofer. Rachel Glick, M.D. is the Associate Chair for clinical and Administrative Affairs and a clinical professor of psychiatry at the University of Michigan, with expertise in emergency psychiatry outcomes and quality. Lisa Brenner, Ph.D. is an Associate Professor for the University of Colorado Denver School of Medicine and the Director of the VA Rocky Mountain Mental Illness Research Education and Clinical Center (MIRECC). Dr. Brenner conducts suicide research with a focus on individuals with traumatic brain injury. Timothy Hofer, M.D. is a senior health services researcher and professor of internal medicine at the University of Michigan with expertise in quality, safety, and analytic methods. Drs. Glick and Hofer served on the DSMB for the R34 pilot study. All DSMB members are free of conflicts of interest with the proposed study. In keeping with the "NIH Policy for Data and Safety Monitoring", additional members will be included in the DSMB meeting as necessary to interpret the data and ensure patient safety. After each meeting, a summary report of discussion and resulting recommendations will be drafted and submitted to the local IRB and to NIH at each annual review.

The Data and Safety Monitoring Plan will be reviewed and approved by the Medical IRB at the University of Michigan. Monitoring is intended to protect subjects' rights and safety, and to ensure the integrity and quality of the data collected. The project coordinator will be responsible for creating a Regulatory Binder, which will contain the C.V. of all of the investigators, research staff, and safety monitoring committee (see below). All of the investigators and research staff involved in this trial will have completed the University of Michigan on-line educational programs on protection of human rights, Good Clinical Practice, and scientific ethics. Documentation of completion will be filed in the Regulatory Binder. The binder will also contain all communications to the IRB, including the initial application, study protocol, any amendments, annual IRB renewal, IRB approvals, and a summary of adverse events. It will be the responsibility of the project coordinator to maintain and update the Regulatory Binder.

The project coordinator will review consent forms, case report forms (CRF), and source data two weeks after the first subject is consented for screening, two weeks after the first subject is randomized, and then on at least a quarterly basis. Each review will be documented in the Monitoring Log and accompanied by a written Monitoring Report to be filed in the Regulatory Binder. The following materials will be monitored at each review:

a. Written informed consent for 100% of subjects
b. Inclusion/exclusion criteria for 100% of subjects
c. Adverse events and serious adverse events for 100% of subjects
d. All communications with the IRB, the sponsor, and other regulatory agencies
e. Any protocol violations
f. Completeness of data collection

In addition, all CRF for the first 3 randomized subjects will be verified completely, including completeness and consistency of questionnaires and interviews. Thereafter, every fifth subject will be verified for 100% of entries. If more than five errors are noted, then every case will be checked at the next review.

The PI will have the following responsibilities pertaining to data safety and monitoring during the course of the study:

a. Review of all adverse events as they occur
b. Review of all breaches of confidentiality if they occur
c. Communication with the IRB, the sponsor, or other regulatory agencies in the event of a serious adverse event or breach of confidentiality according to the specified guidelines
d. Periodic reviews of the literature regarding procedures used in the study to determine if any new information affects the safety or benefit-to-risk ratio of study participation. If such information emerges, then amendments documenting that information will be filed with the IRB.

B. Trial Protocol & Statistical Analyses Plan (Final)

Version Date: December 8, 2021

BACKGROUND & RATIONALE

Background. After years of steady increases, in 2014 the suicide rate in the U.S. reached its highest rate in decades with over 42,000 suicide deaths in that year.(2) Suicide has surpassed motor vehicle accidents as a leading cause of death in the U.S., is the tenth leading cause of death overall, and is in the top 4 leading causes of death for those younger than 65.(17) For every suicide death, approximately 25 times as many people (over 1 million Americans) attempt suicide, resulting in emergency department visits or hospitalizations costing the U.S. $10.4 billion dollars in health care expenditures and lost productivity.(18) The costs of suicide extend to include negative health and social functioning effects on surviving loved ones.(19) The strategic action plan for suicide prevention in the U.S. emphasizes the need for a combination of universal, selective, and indicated interventions.(1) Successful universal strategies include restricting public access to various means for suicide such as firearms, jump points, and toxic substances.(20-23) Other population-level approaches such as gatekeeper training (e.g., training public service professionals to identify and refer individuals at risk for suicide), universal screening in health systems, or crisis telephone/internet services have generally low levels of evidence (i.e., few randomized controlled trials) for preventing suicides or suicide attempts despite instances of wide scale implementation.(4, 24-26) Universal strategies that include linkage of at-risk individuals to additional selective or indicated prevention services may depend on the effectiveness of those subsequent services. However, there are very few interventions shown to be effective for high-risk adults, and even fewer that have been widely adopted by health systems. (4, 27) Selective or indicated prevention interventions include use of brief follow-up contacts (e.g., by phone, mail, or face-to-face) by health professionals after a suicide attempt or hospital discharge. (28-31) The findings of these studies are mixed and limited by samples of only treatment refusers or those without basic access to mental health treatment, though they suggest that even relatively minor expressions of caring may improve some recipients' belongingness. The medications lithium and clozapine have been shown to reduce suicides among those at risk due to a mental health condition, though their side effect burden, risks of serious medical complications, and indications for only a subset of mental health conditions limit their use.(32, 33) Various psychotherapies, including dialectical behavioral therapy, cognitive behavioral therapy, and problem-solving therapy have been shown to reduce the incidence of suicide attempts, though a pooled analysis of these trials demonstrated no effect on suicide deaths.(34) Psychotherapy as a mental health service also faces major barriers related to workforce availability and patient adherence.(35, 36) Receipt of psychotherapy specifically for suicide prevention is not known; however, it is almost certainly underutilized considering less than 20% of patients receive an adequate course of *any* psychotherapy after psychiatric hospitalization.(37, 38) The steady rise in suicide rates is a testament to the need for interventions, such as peer mentorship, that offer new approaches to addressing suicide risk and have potential to be implemented at wide scale.

Peer mentorship and the spectrum of peer support. Peer mentors are individuals who have achieved stable recovery from a mental health condition and who incorporate their personal experiences into providing support to others currently experiencing mental health challenges. Peer mentorship exists within a spectrum of peer support and occupies a middle ground between less structured mutual/reciprocal peer support and more structured forms where peers provide services traditionally delivered by clinically trained professionals (e.g., case management) and rely less on their lived experience of mental health challenges. (Figure 1). (5)

Figure 1. Continuum of Helping Relationships Including Peer Support (adapted from Davidson(5))

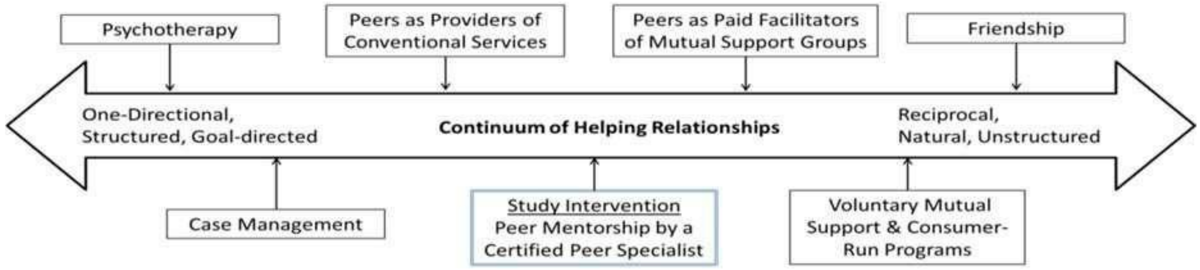

We chose a mentorship model of peer support for the PREVAIL intervention rather than mutual peer support because in a recent study we found a mutual one-to-one peer support intervention was no more effective for depression (a risk factor for suicide) or suicidal ideation than usual care.(39) A group-based mutual support intervention for VA patients with any mental health diagnosis also had null effects with respect to depression, functioning, hope, social support, and self-harm.(40) In contrast, peer mentorship has demonstrated effectiveness for post-partum depression compared to usual care, and peer mentors are as effective as traditional providers of case management, suggesting that peers who can speak to and role model successful recovery may be particularly effective.(8, 41) We did not choose a model where peers act as providers of traditional services because the disclosure of the peer

mentor's own experiences with suicide and recovery is core to the distinct mechanisms by which the intervention might reduce suicide risk (see below).

Intervention mechanisms of peer mentorship. Our theoretical model for peer mentorship as a method for preventing suicide attempts draws from the interpersonal theory of suicide (ITS) and the postulated effects of peer support (Figure 2) (3). The ITS groups empirically-supported suicide risk factors into four domains: hopelessness, thwarted belongingness, burdensomeness, and acquired capability for suicide. The theory posits that active suicidal ideation (e.g., thoughts of killing oneself) occurs when belongingness and burdensomeness occur in the presence of hopelessness. Suicide attempts occur in an actively suicidal individual who also has an acquired capability for suicide. The ITS domains explain a greater portion of the variance of suicidal ideation and attempts than models using demographic characteristics and diagnoses (42). While the theorized interactions between domains have not been consistently replicated, the independent effects of the ITS domains have been repeatedly found to be associated with suicide outcomes, thereby supporting the use of these constructs as targets of this intervention. (43, 44)

Figure 2. Interpersonal Theory of Suicide (adapted from Van Orden(3)) and Peer Mentorship

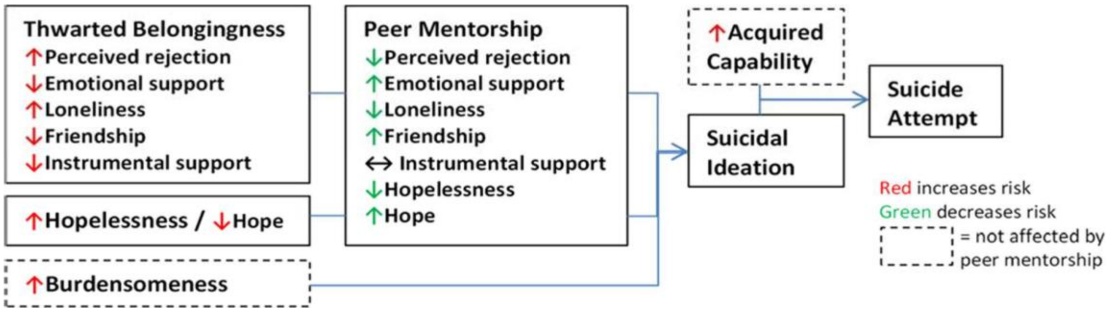

There are many postulated benefits of peer mentorship, notably including improved belongingness achieved by establishing shared experiences of mental health challenges and decreased hopelessness achieved as the peer mentor provides a role model of recovery. While the effects of poor belongingness (e.g., perceived rejection, loneliness, low emotional support) and hopelessness on increased risk of suicide are well established according to several reviews encompassing dozens of trials(3, 45-47), whether peer mentorship improves an individual's sense of belongingness or hopelessness has not been shown definitively as most trials of peer support have not included measures of these constructs.(48) In our review of 40 trials of peer support cited in 3 recent meta-analyses, 17 studies included measures of belongingness with 6 showing positive effects, and 4 studies included measures of hope or hopelessness with 3 showing positive effects.(49- 51) Based on the suggestive but inconsistent evidence that peer mentorship can improve belongingness and hopelessness, PREVAIL was designed with explicit structured content (e.g., identifying reminders of hope) to address these risk factors. In addition to the core mechanisms of suicide prevention drawn from the ITS, peer mentorship can potentially further reduce the risk of suicide through several other mechanisms including: monitoring and detection of escalating risk, greater activation and engagement in treatment, and improved self-management of mental health symptoms. (52) Aim 1 of this study will determine whether the aggregate benefits of peer mentorship are effective for reducing suicide attempts, while Aim 2 will assess whether the intervention has the hypothesized effects on hopelessness and belongingness. (Note: hope and hopelessness are used interchangeably in the writing of this proposal for brevity, as are thwarted belongingness and belongingness, but each may represent separate but correlated constructs and thus will be measured separately).

Implementation of an effective peer support intervention. Although peer support services began historically as an alternative to psychiatric treatment, there has been increasing integration of peer services into mental health treatment with the advent of professionalized, certified peer specialists. (5, 53) Peer specialists are individuals with a lived experience of mental illness who receive formal training, certification, and employment in providing peer support. PREVAIL was designed to be delivered by peer specialists in part because they are already integrated with many community mental health programs and provide a wide range of recovery-oriented, patient-centered services. (54) The State of Michigan has trained and certified 1,640 peer specialists, and an estimated 24,000 have been certified nationwide. (12) In 2014, 34 states also offered Medicaid reimbursement for peer support services, furthering sustainability of these services. (55) The State of Michigan also trains peer recovery coaches, individuals who have lived experience receiving public mental health services specific to substance use, and who serve similar populations in community mental health programs; Michigan offers reimbursements for peer recovery coach services through public substance abuse treatment funds. With the integration of peer providers into mental health treatment services, programs are increasingly involving peers in crisis services. (56-58) However, peer specialist certification training and peer recovery coach training does not currently address suicide or how peers might be most useful to individuals with suicidal ideation or behaviors. Thus, there is likely substantial demand for an effective protocol for peer providers who are supporting high-risk individuals. The training director for peer specialists in the State of Michigan, who has supported the development and study of PREVAIL, would offer training in the intervention immediately to currently employed peer specialists if found effective, as would potentially many other peer training directors. However, the decision by peer specialists, community mental health clinics, and hospitals to implement PREVAIL (if effective) could be negatively or positively influenced by a variety of factors. Prior studies of peer support implementation (59, 60) (including by consultant Chinman) identified confusion about the peer's role within a clinical team as a barrier. Whether the PREVAIL training and

protocols address this barrier, and whether other factors rise in prominence when accounting for the health system perspective (e.g., effect on readmissions) or the context of suicide prevention (e.g., acceptability of safety protocols) requires further, structured examination.(61) Examination of implementation concurrent with effectiveness (i.e., the hybrid trial design) will help explain trial findings if null and will increase population health impact of PREVAIL by reducing delays in implementation if effective.(14)

Preliminary Studies

PREVAIL R34 Pilot Study. All protocols necessary to conduct the proposed fully powered randomized controlled trial to test the effectiveness and mechanisms of PREVAIL have been successfully pilot tested.

Intervention development and peer mentor training. During the intervention development phase of the R34 project, an expert panel including suicide prevention researchers (King, Ilgen, Holloway), peer support researchers (Valenstein, Chinman), peer specialists (Coe, Derosa), the peer support training director for the State of Michigan (Werner), and an inpatient psychiatry unit director (Hirshbein) were convened to create an outline of the essential training needs for peer mentors to work with high-risk patients and address suicide risk according to the interpersonal theory of suicide. The outline of the training and intervention content was then used as the basis for developing the training manual (Other Attachment IIA). This manual was developed by the PI (Pfeiffer) in conjunction with Eduardo Vega and Dequincy Jones of the Center for Dignity, Recovery, and Empowerment (a peer support advocacy, service, and research organization); Kristen Abraham, a clinical psychologist with expertise in peer support; and Debra Levine, a clinical psychologist and post-doctoral fellow. Drafts of the training materials were circulated among the expert panel and revised in an iterative process. The training was then delivered over 3 days to the study's 4 peer mentors utilizing adult learning theory with small group discussions, role plays, and video vignettes. (The term "peer mentor" indicates a peer specialist or peer recovery coach with PREVAIL training.)

Figure 3. Consort Diagram for PREVAIL Pilot Study

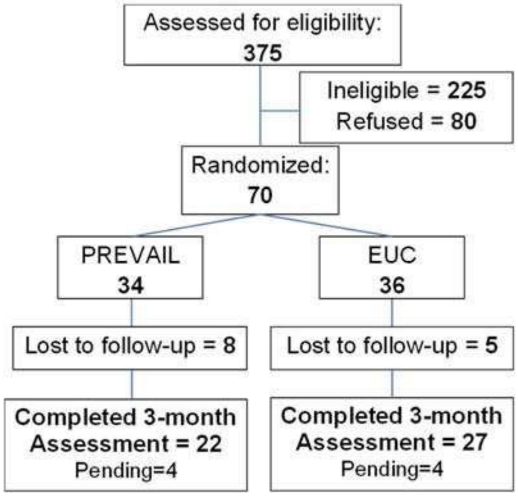

Acceptability and feasibility. A "pre-pilot" in which each peer mentor delivered the intervention to 1 or 2 participants was conducted; the only significant change to the study protocol was to allow mobile device and social media communication with participants. The pilot RCT phase of the study was launched and successfully enrolled 70 participants (Figure 3). 375 patients with medical record documentation of suicidal ideation or suicide attempt at admission were screened, 225 of whom were not eligible and 80 of whom refused participation. The most frequent reasons for ineligibility were unstable psychosis, cognitive disorder, or severe personality disorder as determined by the attending psychiatrist (n=89), distance (n=30), and prolonged hospitalization due to receipt of electroconvulsive therapy (n=30). Participation in the intervention exceeded our target thresholds with 95% of intervention participants completing a meeting with the peer mentor prior to discharge (target 80%) and a mean completion of 5.6 (SD 4.8) peer encounters over 3 months (target mean of 4). The most common reason for not completing any encounters after discharge (n=9) was feeling overwhelmed or too busy; participants were offered to switch to a new peer mentor, but none chose this option, suggesting the reasons for poor engagement were not specific to the individual peer mentors. Excluding participants pending follow-up (pilot study is still collecting follow-up data), 79% (49 of 62) have completed the 3- month assessments and 80% (28 of 35) have completed the 6-month study assessments. Exceeding our target of 60 for enrollment and target of 75% for follow-up assessments demonstrates feasibility to proceed to a larger-scale trial.

Supervision, fidelity, and refinement of the intervention. Fidelity to the intervention, including suicide safety protocols, was maintained by weekly group supervision meetings consisting of the peer mentors and the principal investigator (a psychiatrist) or a co-investigator psychiatrist or psychologist. Each participant encounter was discussed, training materials and intervention protocols were reviewed, and audio-recordings of individual sessions were presented for feedback. Following the initial phases of the trial, the structure of the intervention session content was refined to facilitate fidelity by establishing a consistent format across content areas. The format used the mnemonic "ILSM" which stands for "Invite, Learn, Share, Motivate", and a semi- structured conversation guide was created for each content domain in this format (Other Attachment IIB). The format incorporates motivational interviewing concepts and techniques—encouraging peers to first understand the participant's experiences and ideas for change with intentional sharing of the peer mentor's experiences to offer new ideas and validate shared struggles. The ILSM conversation guides were developed collaboratively with the 4 peer mentor interventionists and study co-investigators. A fidelity measure of adherence to the ILSM format was developed and used to rate sessions via audio-recordings (Other Attachment IIC). The measure, based on the Yale Adherence and Competence Scale(62), uses a 4-point scale to rate the skill (1 representing "very poor" to 4 representing "skilled") and extensiveness (1 representing "not discussed" to 4 representing "a lot of discussion" [e.g., >15 minutes]) with which ILSM conversations are delivered. A rating of 2 or greater on both skill and extensiveness was considered adequate fidelity to the

intervention. Fidelity to general communication and peer support skills (e.g., validation, sharing, avoidance of medical advice) were also rated on the 1 to 4 point "skill" scale with a score of 3 or greater indicating fidelity to the intervention. Two research assistants were trained on use of the fidelity measure until inter-rater agreement was >80%. Among 20 sessions rated using the measure, 85% demonstrated adequate fidelity to the ILSM model and 72.5% of general skills were performed with adequate fidelity. These ratings clearly demonstrate peer mentors were able to incorporate PREVAIL content into their contacts with participants.

Outcomes and safety. Comparisons of outcomes between the intervention and enhanced usual care control groups were not conducted because any finding would be an unreliable estimate of the true effect given the small sample size.(11) The R34 randomized control group allowed pilot testing of all necessary protocols in preparation for this R01 study. Across the entire sample, 24% of participants reported any post-enrollment suicide attempt at 3 months according to the Columbia Suicide Severity Rating Scale.(63) Suicidal ideation according to the Beck Scale for Suicidal Ideation improved from 23.3 (SD 7.6) at baseline to 6.6 (SD 7.7) at 3 months.(64) Despite limited power, improvements on belongingness measures of loneliness and perceived rejection, but not emotional support, were statistically significant at 3 months.(65) There was also statistically significant improvement on a measure of hopelessness, but not hope, at 3 months.(66, 67) These findings demonstrate feasibility of measuring the primary outcomes and potential mediators in a larger trial. A data and safety monitoring board (DSMB) was convened every 6 months to review adverse events. Two participants were readmitted after study staff contacted Emergency Medical Services due to acute suicide risk, and in one of these cases the participant had already overdosed on medication when she met with the peer mentor. One participant randomized to the enhanced usual care condition died by suicide within 2 weeks of enrollment; his only interaction with the study was the enrollment meeting and baseline assessment on the inpatient unit. No adverse events were attributed to study participation. Peer mentors were not treated differently from other research staff in terms of monitoring their mental health; however, there were no incidents in which the mental health of the peer mentors interfered with their work.

Other Studies of Peer Support. Drs. Pfeiffer and Valenstein have conducted prior multi-site RCTs involving peer support, including an ongoing trial that utilizes peer specialists to enhance engagement in computer- based cognitive behavioral (c-CBT) therapy for depression and a completed trial of mutual dyadic peer support among depressed VA patients. The latter of these trials found no differences in improvement in depression among participants randomized to be paired for purposes of mutual peer support compared to those randomized to usual care.(39) These findings directly informed the use of a peer mentor model vs. a mutual support model for the PREVAIL intervention. Drs. Pfeiffer and Valenstein also conducted a single-arm VA pilot study of peer support by a peer mentor following psychiatric hospitalization for depression, which informed the need for the more extensive, suicide-specific training program developed during the R34.(68)

Other Studies of Suicide Risk & Prevention. Dr. King is internationally recognized for her expertise in the conduct of RCTs of suicide prevention interventions for youth and young adults, including completion of a randomized controlled trial of a social network intervention.(69,70) Dr. King has also contributed substantially to our understanding of risk-management and ethical considerations in intervention research to prevent suicide.(71) Drs. King and Pfeiffer served as co-investigators on a DoD-funded randomized controlled trial of a CBT-based suicide prevention trial (PI: Holloway). The core CBT intervention in the study was administered while patients were admitted to a military psychiatric hospital, and Drs. King and Pfeiffer led the development of the post-discharge therapist-delivered booster sessions. Co-I Dr. Ahmedani is a nationally recognized suicide prevention researcher who has investigated patterns of care within health systems prior to suicide and is an advisor to the National Zero Suicide Initiative.(72) Drs. Pfeiffer, Valenstein, and Kim have also previously examined the associations between hopelessness, belongingness, and suicidal ideation.(44)

Implementation and Qualitative Studies. Co-I Dr. Forman is a qualitative methodologist and implementation scientist who has worked with Drs. Pfeiffer and Valenstein on a qualitative evaluation of the implementation of the Buddy 2 Buddy peer support program in the Michigan National Guard and a mixed methods study of barriers and facilitators to receipt of quality depression care following psychiatric hospitalization across two VA medical centers.(36, 73) Dr. Forman is a Co-I on a VA peer-supported c-CBT study which uses a similar hybrid effectiveness-implementation design to the current study. Consultant Laura Damschroder is an implementation scientist and the lead developer of the Consolidated Framework for Implementation Research (CFIR), the conceptual framework guiding Aim 3 of this study.(61) Dr. Forman and Ms. Damschroder have collaborated previously on implementation studies. (74-76) Consultant Dr. Chinman has conducted a series of studies regarding the implementation of peer support services, including the integration of peer specialists into mental health treatment settings across the VA health system. (53)

Study Rationale. New approaches to suicide prevention are needed to meet the rising tide of suicide deaths in the U.S. Peer mentorship represents a novel approach to reducing suicide risk by addressing poor belongingness and hopelessness, two risk factors for suicide supported by theory, evidence, and advocates. This study of peer mentorship will be the first effectiveness trial of a peer-delivered intervention designed to reduce suicide attempts and suicidal ideation. This study advances peer support research by the inclusion of intermediate targets in the intervention design and assessment of potential mediators. A safe and effective peer-based intervention to prevent suicide attempts could achieve wide scale implementation due to a growing, professionalized, and integrated peer specialist workforce that increasingly supports high-risk individuals. The hybrid effectiveness-implementation design used in this study is an innovative approach to reducing delays in translating evidence from clinical trials into practice. A major limitation to clinical trial research is that widespread implementation of effective interventions may take decades or never occurs after publication

of the trial results. (13) Hybrid effectiveness-implementation trial designs are intended to address this problem by studying the barriers and strategies for future implementation simultaneous to the determination of effectiveness. (14) Even if the specific intervention proves not to be effective, assessment of stakeholder experiences with the intervention may provide explanations for the null findings, guide future research, and inform ongoing initiatives to increase peer involvement in suicide prevention.

STUDY DESIGN

We will conduct a single-blind randomized controlled trial of the PREVAIL peer mentorship for suicide prevention intervention compared to enhanced usual care among 490 participants at high-risk for suicide recruited from inpatient psychiatric units.

Study Flow

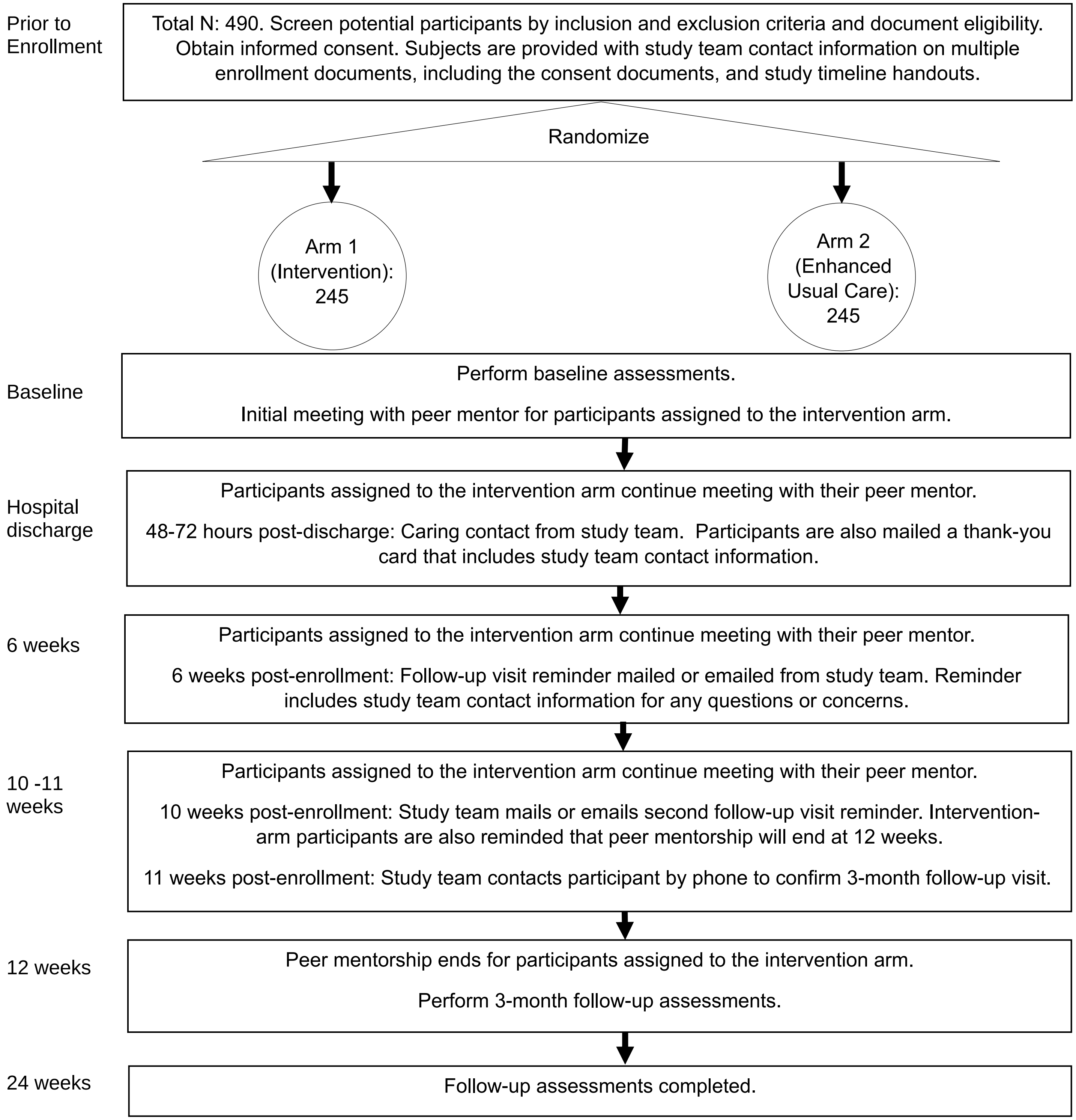

STUDY PROCEDURES

Schedule of Activities

	Pre-screening (Pre-Consent)	Enrollment - Week 1, Day 1	Baseline - Week 1, Day 1	Intervention - Week 1	Intervention - Week 2	Intervention - Week 3	Intervention - Week 4	Intervention - Week 5	Intervention - Week 6	Intervention - Week 7	Intervention - Week 8	Intervention - Week 9	Intervention - Week 10	Intervention - Week 11	Intervention - Week 12	Intervention - Weeks 12-16	Intervention - Weeks 16-23	Intervention - Week 24	Withdrawal
Review eligibility	X																		
Eligibility confirmation		X																	
Informed consent		X	X																
Outcome Evaluation																			
Suicide Attempts (Columbia Suicide Severity Rating Scale)			X												X			X	
Suicidal Ideation (Beck Suicide Scale)			X												X			X	
Medically serious suicide attempts (medical record)															X			X	
Self efficacy (Self Efficacy to Avoid Suicidal Action Scale)			X												X			X	
Hopelessness (Beck Hopelessness Scale)			X												X			X	
Quality of life (Quality of Life Enjoyment and Satisfaction Questionnaire Short Form)			X												X			X	
Functional status (Short Form 12)			X												X			X	
Perceived social support (Multidimensional Scale of Perceived Social Support)			X												X			X	
Depression (Patient Health Questionnaire (PHQ-9))			X												X			X	
Hope (State Hope Scale)			X												X			X	
Burdensomeness (Interpersonal Needs Questionnaire)			X												X			X	
Perceived emotional support (NIH Toolbox Adult Social Relationship Scales)			X												X			X	
Perceived instrumental support (NIH Toolbox Adult Social Relationship Scales)			X												X			X	

Perceived friendship (NIH Toolbox Adult Social Relationship Scales)			X												X			X	
Loneliness (NIH Toolbox Adult Social Relationship Scales)			X												X			X	
Perceived rejection (NIH Toolbox Adult Social Relationship Scales)			X												X			X	
Utilization of health care services (NIH Toolbox Adult Social Relationship Scales)			X												X			X	
Medication adherence (Single-Item Self Rating Scale for Medication Adherence)			X												X			X	
Perceived meaning in life (Meaning in Life Questionnaire)			X												X			X	
Randomization			X																
Enhanced Usual Care & Experimental Interventions – Peer mentorship			X	X	X	X	X	X	X	X	X	X	X	X	X	X			
Event reporting		X	X	X	X	X	X	X	X	X	X	X	X	X	X	X	X	X	X

DESCRIPTION OF ACTIVITIES

Pre-consent activities

Pre-screening – each day, study staff at each recruitment site will review the medical records of patients admitted to their inpatient psychiatric unit to determine if they meet preliminary inclusion criteria. Patients will be eligible for the study if they: 1) are age 18 years or older, 2) are currently admitted to an inpatient psychiatric unit and have medical record documentation of suicidal ideation or suicide attempt at the time of admission, 3) have a Beck Suicide Scale score of 5 or higher for the 1-week period prior to admission, 4) are fluent in English, and 5) are able to be reached reliably by telephone.

Eligibility confirmation – study staff will contact the attending psychiatrist of each patient who meets preliminary inclusion criteria. The attending psychiatrist will determine if peer mentorship is not appropriate due to unstable psychosis, cognitive disorder, or severe personality disorder. Study staff will approach patients who remain eligible, and will administer the Mini-Cog to determine if the patient is unable to provide informed consent due to substantial cognitive impairment.

Informed consent – study staff will obtain informed consent from interested individuals before they are discharged from the inpatient unit. The study staff member will review all study activities, emphasize that participation is voluntary, discuss potential risks and benefits of participating, and review the participant's responsibilities. An individual is considered enrolled once informed consent has been provided.

Post-consent activities and outcomes assessments

Outcome assessments are performed at three time points across an individual's participation in the study: baseline (while the participant is admitted to the inpatient unit), 3 months, and 6 months from their enrollment in the study. Beck Hopelessness Scale, Beck Scale for Suicidal Ideation, Columbia Suicide Severity Rating Scale, State Hope Scale, Interpersonal Needs Questionnaire, Multidimensional Scale of Perceived Social Support, Meaning of Life Questionnaire, Single-Item Self Rating of Medication Adherence, NIH Adult Toolbox Social Relationship Scales, Patient Health Questionnaire (PHQ-9), Quality of Life Enjoyment and Satisfaction Questionnaire Short Form, Self- Efficacy to Avoid Suicidal Action Scale, Working Alliance Inventory – Client, Short Form 12, and health service utilization inventory (lifetime or 3 month interval) – these will be collected via self-reported measures at baseline, 3 month, and 6 month assessments.

Randomization and Blinding. Randomization will be conducted using a minimization algorithm implemented by the University of Michigan's Consulting for Statistics, Computing & Analytics Research (CSCAR) center.(80) The two variables included in the minimization are gender and whether the participant made a suicide attempt immediately prior to hospital admission. These variables were chosen to avoid the potential confounding that could arise if by chance these variables were not equally distributed during random assortment.(81) Randomization will be stratified by study site. After a participant has completed consent procedures and the baseline assessment, a research assistant will access the CSCAR web application, input the two variables, and receive the allocation assignment. All follow-up assessments will be completed by a separate research assistant who will be blinded to study arm assignment.

Control & Experimental Interventions. Once study arm is ascertained, the study team will commence with study intervention activities. The study staff member will schedule the participant's 3 month assessment and provide them with a copy of the Informed Consent Document, and then either (1) the baseline visit will be complete, or (2) the study staff member will instruct the participant to select a peer mentor from a list of short written descriptions of peer mentors currently available to accept a new patient, and will coordinate an initial meeting between the participant and their selected peer mentor to take place on the inpatient unit before the participant is discharged.

Outcome Assessments. The 3 and 6 month outcome assessments should occur within the windows listed on the schedule of activities, through in-person meetings with separate research staff blinded to study arm assignment. In cases where in-person attendance is not feasible, the measures can be completed over the phone or internet, though in these cases the participant's location and availability of an emergency contact person will first be confirmed in the event of acute suicidality. If a participant fails to complete an assessment within the prescribed window, that assessment will be documented as incomplete.

Withdrawal. If participants withdraw or decline to finish the peer mentorship intervention, every efort will be made to gather primary outcome data (Columbia Suicide Severity Rating Scale, Beck Scale for Suicide Ideation). These surveys may be done in person or over the phone. In the event that a participant withdraws before completing all study activities, staff will administer a brief semi- structured Early Withdrawal Interview to gather feedback on the participant's experience with study activities.

Retention

Research staff may encounter difficulty contacting participants for the purpose of completing study activities. In order to minimize attrition and maintain high follow-up completion rates, staff will collect thorough contact information at the time of enrollment by having each participant complete a Participant Locator Form.

Research staff will provide participants with multiple reminders for each follow-up assessment visit. First, research staff will contact participants 48 hours after their discharge from the inpatient unit to re-introduce the study. Staff will mail a reminder postcard to the participant five weeks prior to each follow-up assessment visit, and a reminder letter two weeks prior to each visit. Staff will also call each participant one week prior and 24 hours prior to each scheduled assessment visit.

Research assistants may attempt to reach participants via phone, text, email, social media (Facebook or Instagram), or letter. A study cell phone will be used for phone and text contacts. This cell phone will be password encrypted and stored in a locked cabinet when not in use. Staff will maintain an outgoing voicemail message that provides the phone number for the National Suicide Prevention Lifeline.

Participants will be asked to provide contact information for up to three friends or family members who research staff may contact for assistance in reaching the participant. Research staff will not share any confidential information about study participation with these individuals. In the event that a participant cannot be reached using the contact information they have provided, research assistants will contact these individuals and ask if they can help us get in touch with the participant. Research assistants may contact the provided friends and family contacts via telephone, email, or letter. All email communications to participants or their designated contacts will be made from a study-specific email account (psy-prevail-study@med.umich.edu).

Research assistants will use a study Facebook and Instagram account to send private messages to participants through Facebook or Instagram, if the participant has provided their social media account information at enrollment and agrees that staff may contact them via social media. Research staff will not use study social media accounts to add any participants as “friends” in order to protect participant privacy.

When a participant cannot be reached by phone, research staff will mail a letter to the participant using the permanent mailing address they provided at enrollment, asking the participant to contact study staff.

Peer mentors will be provided study cell phones to contact participants. These cell phones will be password encrypted, and will be stored in a combination lockbox when not in use. Peer mentors may use these cell phones to text participants in order to schedule meetings if they are unable to reach them by phone call. Peer mentors will keep these study cell phones turned of outside of business hours when they are not meeting with a participant, and will maintain an outgoing voicemail greeting that provides the National Suicide Prevention Lifeline phone number.

Peer mentors may email participants for the purpose of scheduling mentorship sessions. Peers will use email for scheduling only if the participant has no working phone available where they can be reached. All email communications from peer mentors will be sent from Level 2 email accounts.

SELECTION AND ENROLLMENT OF PARTICIPANTS

Inclusion Criteria. The goal of the study inclusion and exclusion criteria is to identify a broadly representative sample of inpatient psychiatric patients at high risk for suicide based on a suicide attempt or suicidal ideation prior to admission. Including only those with a suicide attempt would limit the generalizability of the intervention. Recruitment from inpatient units facilitates the initial in- person peer support contact in a safe setting and ensures some intervention has taken place prior to when patients are at maximal risk immediately following discharge. (77) Patients will be eligible for the study if they: 1) are age 18 years or older, 2) are currently admitted to an inpatient psychiatric unit and have medical record documentation of suicidal ideation or suicide attempt at the time of admission, 3) have a Beck Suicide Scale score of 5 or higher for the 1-week period prior to admission, 4) are fluent in English, and 5) are able to be reached reliably by telephone. The age and language criteria are necessary because peer mentors will be English- speaking adults.

Exclusion Criteria. Patients will be excluded if they are: 1) substantially cognitively impaired (according to the Mini-Cog(78, 79); for patients unable to read an analog clock, the Mini Mental State Exam will be administered instead, and for patients who are screened via telephone, the Callahan Six-Item Cognitive Screener (119) will be used), 2) unable to provide informed consent for any reason (including incompetency), 3) determined by the patient’s attending psychiatrist that peer mentorship is not appropriate due to unstable psychosis, cognitive disorder, or severe personality disorder, 4) already receiving or intending to receive individual peer mentorship from a Certified Peer Support Specialist, or group-based peer support with a focus on suicide prevention, on a biweekly or more frequent basis, 5) residing outside of the state of Michigan, 6) planning to be discharged to another inpatient or residential facility, 7) receiving electroconvulsive therapy (which prolongs the hospital stay and complicates informed consent and peer interactions due to risks of memory impairment), or 8) have a legal guardian, meaning that the patient is not able to provide informed consent to participate.

Recruitment. Participants will be recruited from the University of Michigan Health System’s inpatient psychiatry unit (UM) and the Henry Ford Health System’s (HF) psychiatric units at Henry Ford Kingswood and Henry Ford Macomb Hospital – Mt. Clemens campus. The UM 25- bed unit was the primary source of recruitment for the R34 pilot study, whereas the HF site was added near the end of the pilot study to demonstrate feasibility of the intervention in more than one setting, ensure adequate accrual rates for a full

scale trial, and increase the sociodemographic diversity of study participants to improve generalizability of findings. The UM site admits approximately 800 patients per year, primarily from the hospital's Psychiatric Emergency Service, the largest such service for Washtenaw County. Henry Ford Kingswood is a 100-bed hospital located in Oakland County in a suburb of Detroit. We will recruit from a 28-bed adult unit of Kingswood that admits approximately 1200 patients per year. Recruitment from these sites during the R34 pilot study resulted in a population with severe suicidal ideation and frequent prior suicide attempts that is representative of the population in Michigan in terms of race/ethnicity. Henry Ford Macomb Hospital has a 48-bed adult inpatient psychiatric unit serving the Detroit and Macomb County area. Henry Ford Wyandotte Hospital has a 20-bed adult inpatient psychiatric unit located in southern Wayne County. Recruitment from UM and Henry Ford sites during the R34 pilot study resulted in a population with severe suicidal ideation and frequent prior suicide attempts that is representative of the population in Michigan in terms of race/ethnicity (Table 1). Directors of recruitment sites strongly endorse the study.

All patients admitted to the UM and HF inpatient psychiatry units will be initially screened for the intervention via review of their electronic medical records and consultation with the patient's attending psychiatrist. Those who meet initial study inclusion/exclusion criteria will then be approached by the research assistant (RA) to describe the study and complete the remaining screening items.

Enrollment. Based on estimates from the participating sites and pilot study results, we anticipate 480 (60% of 800) patients will be admitted to UM per year with medical record documentation of suicidal ideation or a suicide attempt. We anticipate that the number of patients admitted to Henry Ford Macomb and Wyandotte with medical record documentation of suicidal ideation or a suicide attempt will be similar to that seen at the previous Henry Ford Kingswood site (n=720 per year, or 60% of 1200). Of these, we estimate 60% will not meet eligibility criteria and 22% will refuse, such that 216 could be recruited per year. Enrollment of 490 participants during 3 years of recruitment is therefore feasible. The PI of the study (Paul Pfeiffer) will oversee recruitment via weekly calls with HF site PI (Brian Ahmedani) and coordinators from both institutions. Informed consent will be obtained from all participants (see Protection of Human Subjects). Baseline measures (see D2j) will be completed at the time of enrollment, prior to study arm assignment.

<u>Telephonic Screening and Recruitment During COVID-19 Pandemic</u>

Due to health concerns and limitations imposed on in-person research activities during the COVID-19 pandemic, screening and recruitment of subjects will take place telephonically during this time. Potentially eligible patients will be identified via chart review, and approved for recruitment by their attending provider as usual. Research staff will obtain eligible and approved patient(s)' on-unit cell phone number from inpatient unit staff and call the patient(s) to pitch the study using an oral script. Interested patients will receive a study packet from unit staff that includes (1) Consent to be Screened, (2) Consent to be a Part of a Research Study, (3) a printed copy of study measures, (4) printed copies of peer mentor bios, and (5) a study timeline handout that includes study team contact information. Patients will not have access to smart phones, tablets, or private computers on the inpatient units; therefore, all enrollment activities must take place over the phone, and a waiver of documentation of informed consent must be utilized for these enrollments. Subjects will keep the paper copies of consent forms for their records.
Next, the patient will be asked additional screening questions over the phone. Cognitive screening will be completed using the Callahan Six-Item Cognitive Screener, as this measure can be administered remotely (119). Informed consent to be screened for eligibility will be conducted and obtained verbally, and research staff will administer the Beck Suicide Scale over the phone. If the patient is eligible, research staff will proceed with conducting informed consent to participate in the study verbally.

Once a subject has consented to participate, research staff will collect subject contact information over the phone; this information will be entered directly into the study's securely stored identifiable database. Research staff will then administer the baseline measures over the phone. Subjects will provide their responses verbally, using the printed copy of the baseline measures to facilitate the process by having a visual for each question and answer choices. Research staff will enter subject responses directly into Qualtrics. They will randomize the subject per protocol. Subjects who are randomized to peer support will be informed which peer mentors are currently available, will read the printed copies of their bios, and select which peer they wish to work with. The peer mentor will conduct their first visit with the subject over the phone, while the individual is still hospitalized if possible. Last, research staff will provide the subject with information on their 3-month follow-up assessment visit.

Telephonic Suicide Risk Assessment Protocol. If, during the enrollment phone call, a patient discloses current suicidal ideation with some intent to act on thoughts of suicide while hospitalized, the research staff member will alert the patient's nursing staff immediately by placing the patient on hold and calling the unit's charge nurse, and asking to speak with the patient's assigned nurse, before proceeding with the enrollment visit.

<u>In-Person Recruitment During COVID-19 Pandemic</u>

When institutional policies on in-person human subjects research activities allow, the study team will have the option to conduct in-person recruitment from Michigan Medicine 9C using safety precautions to minimize the risk of COVID-19 transmission. These protocols have been developed in cooperation with Michigan Medicine 9C leadership and align with their policies and preferences for restarting in-person recruitment during the pandemic.

Activities that may be conducted in-person under this protocol include:

- Approaching potentially eligible patients individually, describing the study, and gauging interest
- Eligibility confirmation activities, including screening for substantial cognitive impairment.
- The informed consent process. When enrolling in-person, documentation of consent (via ink signatures) will be obtained on consent forms.
- Administration of screening and baseline measures
    - Baseline survey measures are administered via Qualtrics and participants will enter their responses directly into the survey form using a study laptop or tablet. Based on the study team's experience with verbal administration of baseline measures, we believe that allowing participants to complete these measures themselves using a computer can significantly reduce the length of the enrollment visit, which appears to be a barrier to telephone enrollment. In addition, some participants may feel more comfortable answering sensitive questions on a computer rather than sharing their answers aloud with study personnel, or may have privacy concerns about having measures read aloud on an inpatient unit. Therefore, although this protocol requires transmission of materials (a computer) between study personnel and the participant, we believe it is important to offer this method of survey administration in order to increase the recruitment rate and minimize refusals and incomplete enrollments.
    - As with in-person recruitment prior to the pandemic, measures may be verbally administered and participant responses entered into Qualtrics by study personnel based on participant needs or preferences (for example, if a participant has difficulty or discomfort using a computer).
- Collection of participant contact information and friends / family contacts (Participant Locator Form).
    - To minimize transmission of materials between study personnel and patients, study personnel will first attempt to collect this contact information verbally and enter the participant's responses into the Locator Form themselves. They will only offer participants the option to complete the form using pen and paper themselves if needed (for example, if a participant declines to share this information verbally due to privacy concerns about being overheard on the inpatient unit).
- Randomization using the web app developed by CSCAR
- Commencement of study arm activities. Participants will schedule their 3-month follow-up assessment, and if they have been randomized to peer mentorship, they will select which peer they would like to work with from the list of peers available that day.
- For participants assigned to peer mentorship, the first meeting with their study peer may take place either in person at Michigan Medicine 9C, or over the phone.

At the end of the enrollment visit, the participant will be provided with a folder that contains (1) signed copies of the informed consent documents for their records, (2) the Study Timeline handout specific to their assigned study arm that describes what activities they'll be doing throughout their participation, as well as the date and time of their scheduled 3- month follow-up, and (3) study team contact information. Study personnel will store hard copies of documents, including the study team's copies of signed informed consent documents and the Participant Locator Form, in designated locked filing cabinets at the North Campus Research Complex.

The following activities will continue to be conducted remotely:

- For participants assigned to peer mentorship, all meetings with the study peer after discharge will continue to take place remotely via phone or video call. PREVAIL peer mentors will continue not to visit participants in the community.
- 3- and 6-month follow-up assessments.
- Recruitment may continue to take place over the phone in addition to in-person. Examples of reasons telephone recruitment may continue to be used include but are not limited to: a staff member needing to limit in-person contact due to elevated risk for severe COVID-19 complications, or a potentially eligible patient having a COVID-19 diagnosis or being assessed for COVID-19.

If in the future the study team intends to resume any additional in-person activities, the study will seek regulatory approval before doing so.

When recruiting in person, study personnel will follow the Description of Activities protocols described in 6.2.1 – 6.2.4 for screening, recruitment, enrollment, collection of study measures, and randomization, with the following changes:

Safety Procedures to Minimize Transmission Risk During In-Person Recruitment

- Study personnel will adhere to current hospital and inpatient psychiatry unit policies for health screening, such as use of the ResponsiBlue app, prior to entering the facilities. Any study team member who is experiencing symptoms or was exposed to COVID-19 will adhere to current hospital policies for determining whether they may conduct any in-person recruitment activities.
- Study personnel will adhere to current hospital and inpatient psychiatry unit policies for use of Personal Protective Equipment (PPE) and hygiene while on the premises and when interacting with patients. This may include wearing a surgical mask that covers the nose and mouth at all times, use of protective eyewear, and hand hygiene.

- Study personnel will adhere to social distancing guidelines. This includes avoiding prolonged, close contact (within 6 feet) with patients and hospital staff whenever possible. They will minimize contact with any patients not being recruited. Patients not eligible for recruitment and those who hear the study pitch but are not interested in participating will therefore not have close or prolonged contact with study personnel. When screening or enrolling a patient, study personnel will be required to have prolonged contact (greater than 15 minutes) with that individual. During the enrollment process, study personnel will maintain a distance of at least 6 feet from the patient whenever possible. Closer contact may be briefly required at times (i.e., when passing study materials to the patient), but minimized whenever possible.
- The study team will avoid in-person interaction with any patients currently admitted to 9C who test positive for COVID-19 or who have developed symptoms since admission or are being evaluated for possible COVID-19 infection. A confirmed or potential diagnosis of COVID-19 does not make a patient ineligible for study participation; telephone recruitment may still be used with potentially eligible patients who have tested positive or are being evaluated for COVID-19.
- The study team will minimize the number of staff members visiting the inpatient unit at any given time. Only one member of the study team will be present to meet with a patient for enrollment.
- Screening and baseline measures may be collected on a laptop or tablet via direct patient entry. Study personnel may also provide patients with a pen to fill out or sign study documents, such as the Informed Consent Document. Per Michigan Medicine protocol, any shared materials like this that are handled by patients will first be cleaned and then disinfected using an EPA-registered disinfectant approved for use against SARS-CoV-2 between uses. Paper forms that cannot be sanitized will be stored before they are transported back to the study team's office for filing.
- Prior to the pandemic, the study team used the Mini Cog for pre-consent screening for substantial cognitive impairment. However, because the clock drawing task of the Mini Cog requires transmission of paper and writing utensils between the patient and study personnel, and the study team has successfully used the Callahan Six-Item Cognitive Screener, which does not require any sharing of materials between persons, in its place during telephone recruitment, the study team will continue using Callahan Six-Item Cognitive Screener instead of the Mini Cog for in-person recruitment during the pandemic in order to minimize sharing of materials that could lead to infection.
- Study personnel will wash hands before and after all patient interaction, including between interactions with different patients, and prior to leaving the hospital. If soap and water are not available, they will use an alcohol-based hand sanitizer. Soap and water will be used whenever hands are visibly dirty.
- Study materials such as laptops/tablets, folders, and printed materials will be stored at the study team's office in a locked cabinet prior to use. Study personnel will don PPE and wash their hands prior to handling these materials. They will remove and transport only materials that will be necessary for recruitment each day. Forms or folders that are handled by patients and that do not need to be collected and stored by study staff or given to a participant for their records and do not contain any sensitive information (such as blank consent forms or study timeline handouts) will be disposed of at the hospital, and will never be transported back to the study team's office or re-used for future recruitment.

All study team members will complete the required UM trainings, Human Research During COVID-19 Training Module and the Return-to-Work Training Attestation for Human Research, prior to conducting any in-person recruitment activities.

Contact Tracing Protocol. The study team will maintain comprehensive contact records that include the dates and times of each hospital visit, which study team member(s) attended, and which patients they interacted with, including patients who had directly contact with study personnel but did not enroll. Per Michigan Medicine inpatient psychiatry, all patients admitted to unit 9C are tested for COVID-19; those who test positive are isolated. If a patient develops symptoms during admission, they are immediately separated from other patients and isolated to their room to avoid any further potential exposures until they are fully assessed by a clinician; typically, this assessment also involves a COVID-19 test. If a patient becomes COVID-positive during admission, 9C personnel will notify the PREVAIL project manager, who will refer to study records to determine if an exposure could have occurred. If an exposure could have occurred, the study team will adhere to current Michigan Medicine policies for employee exposure, such as contacting the Michigan Medicine Occupational Health Services (OHS) hotline to confirm if an exposure is considered to have occurred and determine if post-exposure testing or any other prevention steps are required. If a study team member tests positive for COVID-19, they will follow current Michigan Medicine guidelines for notifying the University of Michigan Infection Prevention & Epidemiology (IPE), as well as calling the OHS hotline to report the positive test. Employees with a current or suspected case of COVID-19 will not conduct any in-person study activities in accordance with current OHS guidelines on how long employees diagnosed with COVID-19 must remain off work. Asymptomatic employees awaiting COVID-19 test results due to an exposure will obtain guidance from OHS on whether they may come to work.

<u>Post-Discharge Recruitment</u>

While the study team will make every effort to recruit and enroll eligible patients during their hospital admission, potentially eligible patients who the study team is unable to reach or who are unable to finish enrolling while inpatient may be contacted by the study team for recruitment and enrollment up to one week following their hospital discharge. Candidates for post- discharge recruitment will be

patients who met the eligibility criteria during their hospital admission and were deemed appropriate for study participation by their provider per the criteria. Post-discharge recruitment may also include patients who began the screening and enrollment process while hospitalized, but were discharged before they could finish. In these cases, the post-discharge enrollment process will begin where the enrollment left off during their admission (i.e., if the last step in the enrollment process conducted during hospital admission was obtaining informed consent, the patient may be contacted post-discharge to proceed with the baseline measures and the remainder of enrollment activities per the protocols below).

Eligibility Screening. Study personnel will obtain patient contact information from the medical chart and will make several attempts to contact patients via phone, text, and/or email. When a patient is reached, staff will pitch the study over the phone using the Recruitment Script and gauge interest. Interested patients will be assessed for cognitive impairment using the Callahan Six-Item Cognitive Screener (119). Consent to be screened for eligibility will be conducted per the protocols described below in the section "Post-Discharge Informed Consent" for patients who pass the Callahan screener. Once consent to be screened is obtained, study personnel will administer the Beck Suicide Scale (BSS) verbally over the phone.

Enrollment and Baseline Measures. For patients who remain eligible after administration of the BSS, consent to participate in the study will be obtained per the informed consent protocols described below. Once a subject has consented to participate, study personnel will collect subject contact information for the Participant Locator Form over the phone; this information will be entered by study personnel directly into the study's securely stored identifiable database. The baseline Columbia Suicide Severity Rating Scale (CSSRS) will be administered verbally over the phone, and staff will enter subject responses directly into Qualtrics. The remainder of the baseline measures will be administered either verbally over the phone, or a Qualtrics link will be emailed or texted to the subject to complete the measures online, with instructions to pause before the Credibility-Expectancy Questionnaire (CEQ). Study personnel will remain on the phone while the subject completes these measures. They will then randomize the subject per protocol using the CSCAR randomization app, and the subject will proceed with completing the CEQ. Subjects who are randomized to peer support will be informed which peer mentors are currently available, will be provided with their bios (staff will either read the bios aloud over the phone, or will email or text the subject a Qualtrics link to read the bios online), and will select which peer they wish to work with. Last, staff will schedule the subject's 3-month follow-up assessment visit. After enrollment, the study team will mail or email the study timeline handout, which includes study team contact information and a timeline of their study activities, to the participant.

Privacy and Confidentiality. University-managed phones and a study-specific Level 2 email account will be used for patient communications. Phones are password-protected and enrolled in Intelligent Hub through Michigan Medicine to prevent a breach of privacy if a phone is lost or stolen. Recruitment messages left in voicemails or sent via text message or email will be HIPAA-compliant and will not contain any information about a patient's medical history or treatment or sensitive details about the study. A Post-Discharge Recruitment Message Script will be used for these purposes.

Post-Discharge Informed Consent

It is expected that some patients will not have access to internet, email, or a smart phone, tablet, or computer from home; in these cases, a waiver of documentation of informed consent is requested. Study personnel will read the Informed Consent Script, pausing frequently to check in with the patient and gauge their understanding of what is being asked of them and providing ample opportunity to ask questions. For each signature line in the consent forms, research staff will ask the patient to provide verbal consent. Once consent is obtained, research staff will document the date that the subject consented in the study's de-identified database. The study team will mail these subjects paper copies of consent forms for their records.

For patients who do have access to email and internet via a smart phone, tablet, or computer, documentation of consent will be obtained electronically via Sign Now. The study team will upload the currently approved (1) Consent to be Screened and (2) Consent to be Part of a Research Study forms into Sign Now without changes, adding signature fields for both the patient and Principal Investigator designee. Study personnel will make a copy of each informed consent template and add the patient and themselves as signers. The patient will receive the electronic consent forms via email. They will review the consent form and be provided ample opportunity to ask questions. Both the patient and the study team member will electronically sign the documents, which will then be saved in the Identifiable Data folder of the study's access-restricted Maize drive folder. Electronic versions of the signed documents are automatically emailed to the patient via Sign Now for their records.

Post-Discharge Suicide Risk Assessment and Safety Protocols

At the beginning of the post-discharge enrollment call, study personnel will obtain the patient's current location, for use in the event that a wellness check must be initiated. Study measures that ask about suicidality – the BSS and CSSRS – will be administered verbally over the phone so that positive responses are identified by study personnel in real time. If a subject indicates any level of suicidal ideation or recent attempt on these measures or any time during the enrollment call, the study's suicide risk assessment, which utilizes an algorithm of scripted risk assessment questions and "action steps" dependent on subject responses, will be administered. Based on the level of risk determined by the algorithm, study personnel may perform one or more of the following: a) recommend the subject call or be transferred to the national suicide crisis hotline, the University of Michigan's Psychiatric Emergency Services (PES), or the nearest emergency department to immediately address suicide risk and determine whether emergency medical services

or police should be activated to conduct a wellness check, b) notify the participant's outpatient treatment providers regarding a non-imminent increase in suicide risk, c) notify a designated on-call study team clinician when the suicide risk management algorithm has been activated, and when any questions arise regarding risk management. The on-call study clinician will be available via pager or cell phone at all times when there is potential contact between study personnel and patients. Study phones used for phone recruitment are equipped with an outgoing voicemail message stating that voicemail may not be checked outside of business hours and that the caller should contact 911 or the National Suicide Lifeline in an emergency. Study email messages will include the same information below the signature line.

OUTCOMES

Primary Outcomes

The primary outcome is to determine the effectiveness of the PREVAIL peer mentorship intervention for reducing suicide attempts and suicidal ideation among recently hospitalized adult psychiatric patients at high risk for suicide. In a two-site, randomized controlled trial (N=490), participants in the 3-month peer mentorship intervention arm will be less likely to report a suicide attempt on the Columbia Suicide Severity Rating Scale (CSSR-S) and will report less severe suicidal ideation on the Beck Scale for Suicidal Ideation (BSI) over the 6 months following enrollment compared to those in an enhanced usual care control condition.

1. Suicide attempts (measured by the Columbia Suicide Severity Rating Scale) [Time Frame: 6-months]. Any suicide attempt as measured according to an electronic self-report version of the Columbia Suicide Severity Rating Scale (CSSR-S). The definition of suicide attempt for the primary outcome will consist of any actual suicide attempt, aborted suicide attempt, or interrupted suicide attempt according to the CSSR-S.

2. Change in suicidal ideation (measured by the Beck Suicide Scale (BSS)) [Time Frame: Baseline, 6-months] Patient's current suicidal ideation as measured by the Beck Suicide Scale (BSS). The BSS is a self-report 19-item scale preceded by five screening items. The BSS and its screening items assess thoughts, plans and intent to commit suicide. All 24 items are rated on a three-point scale (0 to 2). Total scores could range from 0 to 38 (if the screening items are included). No specific cut-of scores exist to classify severity. Increasing scores reflect greater suicide risk.

Secondary Outcomes

1. Medically serious suicide attempts (measured by the medical record) [Time Frame: 6-months]. Any suicide attempt as notated in the patients electronic medical record. The definition of suicide attempt for the primary outcome will consist of any actual suicide attempt, aborted suicide attempt, or interrupted suicide attempt. Medical record data on suicide attempts will be collected when a participant reports receiving care at an Emergency Room or hospital for reasons related on mental health or substance abuse on the Heath Services Inventory (HSI) at the 3 and 6 month outcome assessments, or when a participant reports seeking medical attention for suicidal behavior during the Columbia Suicide Severity Rating Scale at the 3 and 6 month outcome assessments. Whenever a participant reports received medical attention on these measures, study staff will ask the participant to provide the name of the treating facility. Before the follow-up visit is completed, the participant will be asked to sign a Request for Outside Records, allowing study staff to request medical records from any facility outside of study performance sites. Signing this form is voluntary. If a participant chooses not to sign a form, the participant's self-report will be used to measure medically serious suicide attempts.

2. Self efficacy to avoid suicidal action (measured by the Self Efficacy to Avoid Suicidal Action Scale) [Time frame: 6 months]

3. Suicidal ideation (worst point; measured by the Beck Suicide Scale (BSS)) [Time Frame: 6 months]

Other Pre-Specified Outcomes

Exploratory analysis on potential mediators will also measure the effects of PREVAIL on the following outcomes, and the potential role of these factors as mediators:

1. Change in hopelessness, as measured by the Beck Hopelessness Scale (BHS), a 20-item measure of the degree of pessimism and negativity about the future. Summed scores range from 0 to 20. Scores provide a measure of the severity of self-reported hopelessness: 0-3 minimal, 4-8 mild, 9-14 moderate, and 15-20 severe. (66)

2. Change in quality of life, as measured by the Quality of Life Enjoyment and Satisfaction Questionnaire Short Form (Q-LES-Q-SF), a 14-item measure of satisfaction with a variety of life domains such as physical health, work, and social relationships. The scoring of the Q-LES-Q-SF involves summing only the first 14 items to yield a raw total score. The last two items are not included in the total score but are standalone items. The raw total score ranges from 14 to 70. Higher level of enjoyment and satisfaction with life are reflected in higher scores. (96)

3. Change in functional status, as measured by the Short Form 12 (SF-12), which covers domains including: (1) physical

functioning; (2) role-physical; (3) bodily pain; (4) general health; (5) vitality; (6) social functioning; (7) role emotional; and (8) mental health. Summary scores are calculated by summing factor-weighted scores across all 8 subscales, with factor weights derived from a US-based general population sample. Physical and Mental Health Composite Scores (PCS & MCS) are computed using the scores of twelve questions and range from 0 to 100, where a zero score indicates the lowest level of health measured by the scales and 100 indicates the highest level of health. (100)

4. Change in perceived social support, as measured by the Multidimensional Scale of Perceived Social Support (MDPSS), which contains 12-item Likert scale items with three subscales to address diferent sources of support: family, friends, and significant other (118)

5. Change in depression, as measured by the Patient Health Questionnaire (PHQ-9), a measure of the severity of depression. (103, 104)

6. Change in hope, as measured by the State Hope Scale, a 6-item measure that contains two sub-scales reflecting respondents' personal capacity for change (agency) and knowledge regarding how to achieve change (pathways) (114).

7. Change in burdensomeness, as measured by the burdensomeness subscale of the Interpersonal Needs Questionnaire (INQ).

8. Change in thwarted belongingness, as measured by the thwarted belongingness subscale of the Interpersonal Needs Questionnaire (INQ).

9. Changes in perceived emotional support, as measured by the 8-item emotional support scale of the NIH Toolbox Adult Social Relationship Scales (ASRS) (65)

10. Changes in perceived instrumental support, as measured by the 8-item instrumental support scale of the NIH Toolbox Adult Social Relationship Scales (ASRS) (65)

11. Changes in perceived friendship, as measured by the 8-item friendship scale of the NIH Toolbox Adult Social Relationship Scales (ASRS) (65)

12. Changes in loneliness, as measured by the 5-item loneliness scale of the NIH Toolbox Adult Social Relationship Scales (ASRS) (65)

13. Changes in perceived rejection, as measured by the 8-item perceived rejection scale of the NIH Toolbox Adult Social Relationship Scales (ASRS) (65)

14. Self-reported utilization of health care services, as measured by the adapted Health Services Inventory (87).

15. Medication adherence, as measured by the Single-Item Self Rating (SISR) Scale for Medication Adherence

16. Change in perceived meaning in life, as measured by the Meaning in Life Questionnaire (MLQ), a 10-item measure of the presence of and search for meaning in life (115).

STUDY INTERVENTIONS

Delivery and Duration

The peer mentorship intervention will be delivered by peer providers – peers specialists or peer recovery coaches - trained and certified by the State of Michigan. Michigan is a leader in the training and deployment of peer specialists with over 1,600 peer specialists currently trained. The State training for peer specialists consists of 24 hours of didactic and group-based learning sessions covering role definitions and boundaries, effective communication, combating negative self-talk, problem solving, and supporting recovery through the use of the peer specialist's own recovery story and other recovery dialogues, while the training for peer recovery coaches focuses on multiple pathways to recovery and motivational interviewing. Peer specialists and peer recovery coaches are offered the same continuing education programs provided by the state, and both types of peer providers utilize their lived experience with recovery to support others. The study will hire certified peer providers with at least one year of professional experience providing peer support to individuals with mental health conditions. Peer specialists will also be required to be in stable recovery (i.e., continuous employment with no psychiatric hospitalization in the past year) and must be capable of speaking to a lived experience with suicidal thoughts or behaviors.

Peer providers will be hired for the study from the pool of those already trained in the intervention during the R34, and new peer

providers will be recruited with the assistance of the director of peer specialist training in the State of Michigan, Pam Werner, who maintains a database of all state-trained peer specialists. Ms. Werner served on the expert panel that developed the PREVAIL intervention, assisted with hiring during the R34, and will continue to support the project throughout the R01 (see Letter of Support). Ms. Werner will facilitate recruitment of peer specialists for the study via direct personal contacts and e-mail announcements to peer specialists who reside in or near Washtenaw and Oakland Counties. In order to allow participants the opportunity to choose from among several PREVAIL peer mentors, we will hire 6 to 8 peer providers with diverse backgrounds to deliver the intervention on a part-time hourly basis. We do not anticipate difficulty hiring peer providers based on our experience during the R34 and with other peer support research trials.

Concomitant Treatment. Contamination of the EUC group would most likely occur via receipt of non-study sources of peer support. We will exclude participants who are currently receiving or intending to receive either individual peer mentorship from a Certified Peer Support Specialist, or group-based, suicide prevention-focused peer support, on a biweekly or more frequent basis outside of the study, and will measure non-study peer support via the adapted Health Services Inventory measure. (87) Indirect contamination could occur via communication between PREVAIL and EUC participants while on the inpatient unit, though any such occurrences are expected to be of minor significance relative to the subsequent greater dose of peer mentorship received by PREVAIL participants.

Allowed Interventions. Once a participant is enrolled into the study, there is no restriction on the types of other care they may receive during their participation. If a participant is assigned to the peer mentorship intervention arm, study staff will send a letter to the participant's outpatient mental health provider informing them that the patient will be receiving peer mentorship as part of this study.

Prohibited Interventions. Participants are not prohibited from receiving any other type of intervention once they are enrolled in this study. Participants are informed during the enrollment visit that participation in more than one study at the same time may increase risks to them, and encouraged to gain approval from researchers before taking part in more than one study.

Lifestyle Considerations. Participants are not required to alter any lifestyle behavior or activities while participating in this study.

Intervention Discontinuation. Researchers may discontinue a participant's involvement in the PREVAIL intervention if it is not in the participant's best interest to remain in the study. If a participant informs the researchers that they wish to stop participating in intervention activities, the researchers will inform the participant that they may still complete the 3 and 6 month outcome assessments. Every effort will be made to retain participants who do not complete the prescribed research intervention in order to collect outcome measures from them.

Treatment Fidelity. Peer mentors will attend a weekly group supervision meeting (one for each recruitment site) led by a psychiatrist or clinical psychologist to review each active participant. The meeting allows clinicians to provide guidance regarding difficulties the peer mentors may encounter that are beyond their training or scope. The meetings also reinforce fidelity to the intervention by reviewing training materials, planning future sessions, or reviewing audio-recorded portions of prior sessions to provide feedback on the use of ILSM conversations and general support skills. The group format also allows peer mentors to receive support from other mentors. Fidelity to the PREVAIL intervention will also be monitored through formal fidelity ratings of a random sample of 20% of each peer mentor's audio-recorded sessions utilizing the fidelity rating scale developed during the pilot study (see C1c). Peer mentors who deliver a session without adequate fidelity will receive individual supervision and more frequent monitoring.

Suicide risk is managed by peer mentors via a structured protocol (Other Attachment IIA, page 39-40). At each session, peers ask participants if they are thinking about suicide or have made a recent suicide attempt. If endorsed, additional questions regarding plans and intent are asked. Depending on the participant's responses, the peer mentor will immediately contact a study clinical supervisor (psychiatrist or clinical psychologist) who may speak with the participant to determine the appropriate disposition (e.g., activate emergency medical services) or provide further instructions. The participant's mental health clinician(s) will also be notified of an increase in suicide risk. This protocol was successfully used during the R34 pilot study, and in two instances participants were referred to emergency medical services based on acute risk.

Overall Compliance. A participant will be done with the intervention when they have completed the last session with the peer mentor, which will typically occur within one week of the 3-month outcome assessment.
Treatment Design. During the intervention development phase of the R34 project, an expert panel including suicide prevention researchers (King, Ilgen, Holloway), peer support researchers (Valenstein, Chinman), peer specialists (Coe, Derosa), the peer support training director for the State of Michigan (Werner), and an inpatient psychiatry unit director (Hirshbein) were convened to create an outline of the essential training needs for peer mentors to work with high-risk patients and address suicide risk according to the interpersonal theory of suicide. The outline of the training and intervention content was then used as the basis for developing the training manual (Other Attachment IIA). This manual was developed by the PI (Pfeiffer) in conjunction with Eduardo Vega and Dequincy Jones of the Center for Dignity, Recovery, and Empowerment (a peer support advocacy, service, and research organization); Kristen Abraham, a clinical psychologist with expertise in peer support; and Debra Levine, a clinical psychologist and post-doctoral fellow. Drafts of the training materials were circulated among the expert panel and revised in an iterative process.

Interventionist Training. The PREVAIL intervention training will be delivered to new hires over 3 days according to the intervention training manual (Other Attachment IIA). The training will be modified slightly from the R34 training in that the ILSM conversations (Other Attachment IIB) will be integrated within appropriate modules, and the "PAUSE" communication skills module will removed due to poor fit among the peer mentors during the R34. The first day of the PREVAIL training is intended to review the basic skills of peer support from the perspective of working with individuals at high risk for suicide. This includes a focus on listening and validation skills. The second day focuses on specific techniques for addressing hopelessness and belongingness. This includes sharing of one's own recovery story as it relates to suicide, developing physical reminders of hope (e.g., a hope kit), setting hopeful goals, increasing the support one receives from others, and coping with loss. The third day focuses on skills related to motivational interviewing and addressing suicidal crises (e.g., reinforcing safety plans, self- soothing coping skills, and escalating management to a clinical supervisor). The training also includes modules on prevention of vicarious trauma among peer mentors, working with individuals who are difficult to engage, and guidelines for tailoring session content to the individual. The training includes a mix of small group discussion, role plays, and exemplar video vignettes.

Support and Supervision of Peer Mentors. Peer mentors will attend a weekly group supervision meeting led by a psychiatrist or clinical psychologist to review each active participant and receive clinician guidance regarding difficulties the peer mentors may encounter that are beyond their training or scope. A study clinician is also on-call any time a peer mentor is meeting with a subject to assist with clinical issues or risk management as needed.

Additionally, the study investigator will conduct biannual, one-on-one meetings with each peer mentor to review their performance and address any concerns raised by the peer mentor, including any concerns about the impact of the study on their own mental health. In the case of a participant death or medically serious suicide attempt, a postvention will be initiated modeled after the protocol developed for University of Michigan department of psychiatry faculty and staff. One of the study team investigators will meet with the peer specialist who had contact with the participant to provide emotional support and reassurance, recommend he or she consider receiving additional support from a mental health provider not affiliated with the study, and inquire whether they want to take a break from study activities or leave the study. If they choose to continue with the study, a follow-up meeting will occur to assess their continued ability and comfort working with high-risk individuals.

Maintaining Appropriate Boundaries Between Peers and Participants

All peer mentors will be trained on maintaining professional therapeutic boundaries with participants. Training will include review of the Certified Peer Support Specialist Code of Ethics, review of the study's policies on therapeutic boundaries and complaints resolution.

Internal Policy on Therapeutic Boundaries

The interactions of all PREVAIL staff with participants will abide by the following:

- Touching of a participant is limited to shaking hands and patting of the back/shoulder area. The only exceptions to this is in the instance when a participant has physical limitations which requires assistance with activities of daily living or ambulation.
- Gifts are not to be accepted from participants with the sole exception of a greeting card.
- Each visit to a participant's home must be arranged with and consented to by the participant. Uninvited visits to the participant's home, place of work, or any other expected location are not allowed.
- Sharing personal pictures of family and friends should be limited to portrait-style photos in good taste.
- Disclosure of personal information not directly related to a therapeutic treatment intervention is discouraged.
- Stafff are not to provide participants' identifying data to persons outside the PREVAIL research team, including personal telephone or residence address information unless required by law or in case of an immediate risk of injury or death.
- Stafff may not assist a participant with a personal hygiene–related activity.
- Stafff are to seek immediate clinical supervision if at any point a participant is intoxicated or inappropriately clothed.
- During home visits, staff are prohibited from meeting in a bedroom with the door closed or sitting on a participant's bed.

Participant Complaint Remedial Action Policy

PURPOSE: Establish a standard for disciplinary action relative to substantiated participant complaints related to interactions with staff.

DEFINITIONS

- ABUSE – CLASS I: a non-accidental act, or provocation of another to act, by research staff which contributes, causes or could have caused death, serious physical harm or sexual abuse of a participant.

- ABUSE – CLASS II:
  - A non-accidental act, or provocation of another to act, by research staff which caused or contributed to non-serious physical harm to a participant; or
  - The use of unreasonable force on a participant by research staff, with or without apparent harm; or
  - Any action, or provocation of another to act, by research staff, which causes or contributes to emotional harm to a participant; or
  - An action taken on behalf of a participant, by assuming incompetence, although a guardian has not been appointed or sought, which results in substantial economic, material or emotional harm to the participant.
- ABUSE – CLASS III: the use of language or other means of communication by research staff to degrade, threaten or sexually harass a participant.
- ADULT: a person 18 years of age or older.
- BODILY FUNCTION: the usual action of any region or organ of the body.
- NEGLECT – CLASS I: Acts of commission or omission by research staff which result from non-compliance with a standard of care or treatment required by law, rules, policies, guidelines, written directives, procedures or individual plan of service and which cause or contribute to serious physical harm to a participant; or the failure to report abuses or neglect of a participant when the abuse or neglect results in death or serious physical harm to the participant.
- NEGLECT – CLASS II: Acts of commission or omission by research staff which result from non-compliance with a standard of care or treatment required by law, rules, policies, guidelines, written directive, procedures or individual plan of service and which cause or contribute to non-serious physical harm or emotional harm to a participant; or the failure to report abuse or neglect of a participant when the abuse or neglect results in non-serious harm or emotional harm to the participant.
- NEGLECT – CLASS III: Acts of commission or omission by research staff which result from non-compliance with a standard of care or treatment required by law, rules, policies, guidelines, written directive, procedures or individual plan of service which either placed or could have placed a participant at risk of physical harm; or the failure to report abuse or neglect of a participant when the abuse or neglect places a participant at risk of serious or non-serious harm.
- NON-SERIOUS PHYSICAL HARM: physical damage suffered by a participant and which, at the time of examination by a registered nurse or physician, could not have caused death, or is determined not to be an impairment of bodily function or determined to be a temporary disfigurement.
- PHYSICAL MANAGEMENT: physical containment of a participant by direct contact between staff and participant for the purpose of restricting a maladaptive behavior.
- SERIOUS PHYSICAL HARM: physical damage suffered by a participant which, at the time of examination by a physician or registered nurse, is determined to have caused or could have caused death, or is determined to have caused an impairment of bodily function or determined to be a permanent disfigurement.
- RESEARCH STAFFF: any person employed by or under contract with the PREVAIL research study while conducting activities on behalf of the study.
- SEXUAL ABUSE: any sexual contact between research staff and a participant.
- SEXUAL CONTACT: the intentional touching or penetration of a participant's intimate parts (genitals, buttocks, breasts, groin, inner thigh or rectum) or the intentional touching of the clothing which covers those intimate parts, if that action can be reasonable seen as being for the purpose of arousal or gratification.
- INTIMATE PARTS: the genitalia, buttock and breast of a human being, as well as the groin, inner thigh and rectum.
- EMOTIONAL HARM: impaired psychological functioning, growth or development of a significant nature, as determined by a psychiatrist or psychologist.
- UNREASONABLE FORCE: means physical management or force that is applied by an employee, volunteer or agent of a provider to a participant where there is not immediate risk of physical harm to staff or other participants and no immediate risk of significant property damage and that is any of the following:
  - Not in compliance with approved behavior management techniques.
  - Not in compliance with the participant's individual treatment plan, and/or
  - Used when other less restrictive measures were not attempted.
- SUBSTANTIATED COMPLAINT: to support with proof or evidence.
- UNSUBSTANTIATED COMPLAINT: An allegation that cannot be supported by the preponderance of evidence standard of proof.

<u>PROCEDURES</u>:

- Complaints received directly from participants or any actions that could be the basis of a complaint will be investigated at the direction of the principal investigator.
- A substantiated complaint coded as Abuse Class I, II, and/or III is grounds for immediate termination of employment.
- A substantiated complaint coded as Neglect I is grounds for immediate termination of employment.
- A substantiated complaint coded as Neglect II will result in disciplinary action up to and including termination.
- A substantiated complaint coded as Neglect III will result in disciplinary action.

- Failure to report abuse or neglect of a participant when abuse or neglect occurs, regardless of the abuse or neglect class will result in disciplinary action up to and including termination.
- The recurrence of a substantiated complaint within a 12-month period will result in the termination of employment.
- Substantiated complaints made against the same staff member more than 12-months apart demonstrating patterns of non-compliance will result in disciplinary action up to and including termination.
- Staff who are involved in a complaint that involves Abuse Class I, II, and III and/or Neglect I may be placed on an unpaid leave of absence until the investigation has concluded and a determination has been made.

Delivery of Treatment
Participants randomized to the peer mentorship intervention will be given a short, written description of each of the peer mentors based on who is currently available to accept additional participants. These descriptions are written in the peer mentor's own voice and generally include some demographic information, description of his or her prior mental health challenges, and philosophy of peer support. These descriptions are intended to improve fit between participant and peer mentor and provide the participant with agency in establishing the relationship, as one would have with a natural support.

The peer mentor selected by the participant will then meet with the participant the next day while the participant is still in the hospital. This first session is intended to break the ice, establish "peerness" by the peer mentor briefly sharing some of his or her personal experience with mental health challenges and/or suicidal thinking or behaviors, and discuss how future sessions will proceed and the limits of confidentiality, particularly in the case of acute suicide risk. Subsequent sessions will be scheduled according to the participant's preferences, with a suggested maximum frequency of twice weekly for the first two weeks, weekly for weeks 3 to 8, and then every other week for the last month. In the R34 trial, participants' preferences and availability for contact varied widely, though several participants who completed only a few mentorship sessions still expressed appreciation of the visits during semi-structured qualitative assessments at 3 months. Allowable meeting locations include public places in the community (e.g., coffee shop, park), the participant's home, or a research clinic space. Meetings can also be by phone, which comprised 26% of sessions in the R34.

Preference for the 3-month timeframe was assessed and confirmed by peer mentors and participants during the R34. In order to minimize possible distress that participants may experience at the loss of support at 3 months, and to protect participants to whom an abrupt end to the peer relationship may be harmful, peer mentors may offer participants the option of 3 additional sessions over 4 weeks following the end of the 3-month intervention. These additional sessions will take place over the phone.

Session content is flexible and allows for the peer mentor to provide general supportive listening, validation, and sharing. Session duration is on average 1 hour with at least 15 minutes discussing hope or belongingness according to the ILSM conversation guides. The choice of conversation is determined by the peer mentor based on the participant's needs with input during group supervision. The session structure and content are intentionally highly flexible to allow for genuineness in the peer relationship, thereby increasing acceptability and implicit belongingness. One exception to this flexibility is that during the first session after hospital discharge, peer mentors are instructed to review the participant's suicide safety plan that was created during their inpatient stay and revise as necessary. The 6-week session is also a "check in" point for the peer mentor to gauge preferences for more- or less-structured conversations or sharing and remind participants they are half-way through the intervention so they anticipate termination at 3 months. Without this reminder, some participants were distressed (e.g., tearful, but not suicidal) at the loss of support at 3 months during the pilot.

Enhanced Usual Care Condition. An enhanced usual care (EUC) control condition was chosen: 1) to address ethical concerns that high-risk study participants should receive some enhancement in care, 2) to address concerns that comparing peer mentorship to no enhancement in care lacks equipoise, 3) to increase expectancy and credibility among control participants, and 4) to increase external validity by demonstrating the intervention is superior to another credible health system approach to suicide prevention.(82) The EUC condition will consist of a "caring message" from the study team via e-mail or text message (based on the participant's preference) 24-72 hours after discharge. An example message is, "We hope things are going well for you since you left the hospital. If you wish to reply, we'd be glad to hear from you". A list of local mental health resources will be available if participants reply and during the 3 and 6-month follow-up assessments.

The EUC condition is modeled on prior studies of caring letters and brief contacts by health professionals after suicidal crisis and national recommendations to provide post-crisis follow-up contacts.(31, 83-86) Responses will be addressed by a research assistant trained in suicide risk protocols; after-hours responses will be automatically sent crisis resources.

Participants randomized to the peer mentorship arm will also receive EUC.
A control condition consisting of contacts with a peer specialist without PREVAIL training was not chosen due to ethical concerns that well-intended but poorly trained individuals may provide harmful messages to suicidal individuals ("suicide is selfish", "suicide is an easy way out") or fail to detect or manage acute risk. An attention-only control group using trained professionals would diminish the relative effect of peer mentorship (attention is a nonspecific but active component of the intervention) and would require an increase in

sample size that would greatly increase the complexity and cost of the trial. We decided it would be a more judicious and pragmatic use of resources to first test the effectiveness of PREVAIL compared to EUC, and consider future comparative effectiveness trials if effectiveness is demonstrated in this initial trial. We note we will measure the hypothesized mechanisms of peer mentorship and, if effective, future studies could explore differences between peer mentorship and non-specific attention on these intermediate outcomes.

SAFETY

Potential Risks. A potential risk to study participants is clinical deterioration of their psychiatric condition and/or an increase in suicidal thoughts or behaviors related to receiving the peer mentorship intervention. This risk is anticipated to be minimal based on the conduct of the R34 pilot study, where suicidal ideation improved for the whole population from baseline to 3 months according to the Beck Scale for Suicidal Ideation, and no adverse events were attributed to study participation. Additionally results of a prior RCT showed peer mentorship resulted in fewer psychiatric hospital readmissions (Sledge et. al., 2011); another large RCT demonstrated that peer mentorship was effective for preventing worsening depression (Dennis et. al., 2009). An additional RCT of mutual peer support for depression conducted by study team members (Valenstein et. al., 2015) showed no indication of clinical deterioration or increased suicidal ideation from participation in the intervention.

Another potential risk to study participants is loss of confidentiality related to self-report assessment data, medical record data, or audio recorded sessions or interviews. The risk of an inadvertent breach of confidentiality is minimal (see below regarding protections against risk). Because of the nature of the study (i.e., peer mentorship to reduce future suicide risk), it is necessary to obtain detailed information about past and current suicidal thoughts, plans and behaviors. Loss of confidentiality may also occur in the event that the participant is assessed by a study clinician to be high risk for suicide and a breach of confidentiality is conducted to ensure the participants safety (e.g., contacting a participant's family member or health care provider). Such a breach would only occur if there is no reasonably safe alternative. The informed consent process and documents will explain mandatory reporting requirements for information regarding intention to harm self or others (e.g., suicide, homicide) prior to participating in the study.

There is also a minimal risk of psychological discomfort to study participants from the questions asked in the assessments. Participants may become anxious or uncomfortable as a result of being asked personal questions. The study team members conducting assessments are trained to respond to this emotional distress and to refer the participant to their mental health provider or other appropriate resources as necessary. All participants are free to terminate the assessments at any time or refuse to respond to any questionnaire item. Additionally, there is a popular misperception that enquiring about suicidal thoughts, plans or prior behaviors could increase the likelihood that an individual will make a suicide attempt. Research has not found that suicide assessments increase the risk of suicidal behaviors and this misperception has had the unfortunate consequence of decreasing research on potentially suicidal individuals (see review in Pearson et al., 2001). However, the study of individuals at elevated risk for suicide does require a clear set of procedures to manage potential crisis situations. These are reviewed in detail in the Data Safety and Monitoring plan.

Potential Benefits. It is believed that research participants may be helped in a number of ways. Participants who take part in the peer mentorship arm may benefit from an improved sense of hope and belongingness as the peer mentor establishes a supportive relationship and role-models recovery. The peer mentors are trained and supervised to handle potential distress caused by participating in the intervention conditions. The peer mentors will have immediate "on call" access to a supervising mental health clinician at all times they are meeting with a participant. The peer mentorship condition includes standard care treatment through the University of Michigan and Henry Ford Health System inpatient psychiatry units, including assessments and referrals if deemed appropriate by the clinical staff. All participants in the control condition will receive the same standard of care. In addition, participants will receive a caring contact 24 to 72 hours after discharge and written referral information regarding services and community resources for individuals experiencing suicidal thoughts, plans or related psychiatric problems. The potential benefits for the research are expected to outweigh the risks to participants.

Assessment of Potential Risks & Benefits. After years of steady increases, in 2014 the suicide rate in the U.S. reached its highest rate in decades with over 42,000 suicide deaths in that year. Suicide has surpassed motor vehicle accidents as a leading cause of death in the U.S., is the 10th leading cause of death overall, and is in the top 4 leading causes of death for those younger than 65. For every suicide death, approximately 25 times as many people (over 1 million Americans) attempt suicide, resulting in emergency department visits or hospitalizations costing the U.S. $10.4 billion dollars in health care expenditures and lost productivity. The costs of suicide extend to include negative health and social functioning effects on surviving loved ones. Preventing suicide is a priority across a range of US federal health departments, and new approaches to suicide prevention are critically needed. Randomized controlled trials have shown that peer support interventions are effective in reducing symptoms of depression, reducing the likelihood of psychiatric hospital readmission, increasing hopefulness, and improving quality of life. However, there are few data on the effectiveness of these approaches in suicide prevention. Developing an effective intervention for preventing suicidal behaviors would represent a major advancement given the few existing effective interventions to date. This study will add to the knowledge base in this critical area. Given the potential for this study to improve the quality of life for patients with varying levels of access to evidence-based mental health services, the potential benefits outweigh the risks outlined above.

<u>Event Reporting Schedule</u>

Any adverse events not listed as "expected" below will be reported to the UM or Henry Ford Health System IRB per standard reporting guidelines when relevant, and to the DSMB and the Project Oficer for this grant. The person responsible for reporting adverse events will be Dr. Pfeiffer. The timing of the reporting of any adverse event to the IRB and DSMB by Dr. Pfeiffer will be dependent on the severity of the event. For all *related* adverse events not listed as "expected" below, we will adhere to the standard reporting guidelines regarding the timing of report. More specifically, life-threatening adverse events will be reported as soon as possible but within 7 calendar days; non-threatening potentially serious adverse events that are causally related to the research will be reported in writing within 14 calendar days, and non-serious adverse events will be reported in aggregate form with annual reviews.

<u>Expected Adverse and Serious Adverse Events:</u> Given that this study is recruiting from inpatient psychiatric units, and that the eligibility criteria include suicidal ideation immediately prior to current inpatient psychiatric admission, there are several serious and non-serious adverse events that are expected, not as result of the study activities, but rather as a result of the characteristics of the study population. The following adverse events are considered expected as a result of the characteristics of the study population:

1. Suicide-related death
2. Medically serious suicide attempts (e.g., requiring *medical* hospitalization)
3. Psychiatric hospitalizations or emergency room visits due to suicidal intent or behavior
4. Suicide attempts not requiring medical hospitalization
5. Breach of confidentiality associated with reporting suicidality to agency staff, appropriate authorities, and/or mental health personnel.
6. Suicidal thoughts

Though these adverse events are considered expected for the study population; determining that an event is "unrelated" to study activities requires comprehensive evaluation and tracking for the population of this study. In order to ensure patient safety and monitor any disparity between the intervention and enhanced usual care groups, a study specific reporting plan will be used for the two most serious expected adverse event categories. Suicide deaths and medically serious suicide attempts will be reported to the IRB as "unlikely related" at minimum, as potential changes in trajectory due to study participation should be monitored for both intervention groups. A brief records review will be completed for suicides and hospitalizations in order to confirm that there is no mention of study participation as a causal factor. Please see below for reporting timetable and guideline detail. These events will be reported to the DSMB bi-annually, unless frequency increases beyond that which is expected for the population. Of note: If there is evidence that an adverse event is "related" to study participation, it will be reported individually within 14 calendar days of study team discovery per standard reporting guidelines.

<u>Reporting Plan</u>

Note: Adverse events ***not*** *listed above will be reported using the standard reporting guidelines and timetable*

Event	Relation to study	Reporting Timeframe
Suicide death	Reported as "unlikely related" at minimum.	Individually within 7 days of study team discovery.
Medically serious suicide attempt	Reported as "unlikely related" at minimum.	Individually within 7 days of study team discovery.
Psychiatric hospitalizations or emergency room visits due to suicidal intent or behavior	Unrelated	Not reported per standard reporting guidelines.
	Related	Reported individually within 14 calendar days of study team discovery per standard reporting guidelines.
Suicide attempts not requiring medical hospitalization	Unrelated	Not reported per standard reporting guidelines.
	Related	Reported individually within 14 calendar days of study team discovery per standard reporting guidelines.
Breach of confidentiality associated with reporting suicidality to agency staff, appropriate authorities, and/or mental health personnel.	Unrelated or Related	Not reported per standard reporting guidelines.
Suicidal thought	Unrelated or Related	Not reported per standard reporting guidelines.

Safety Monitoring
The Principal Investigator, Dr. Pfeiffer, ultimately will be responsible for monitoring the data and safety with involvement from all of the study investigators. It should be noted that all research projects involving human participants at the University of Michigan require approval from the University of Michigan Medical School's Institutional Review Board (IRB); IRB approval will also be obtained from the Henry Ford Health System IRB. In addition, because of the sensitive nature of the data being collected, a Certificate of Confidentiality will be obtained for this study from the National Institute on Health (NIH).

Dr. Pfeiffer will ensure that all relevant IRB policies, procedures and stipulations are being followed. Dr. Pfeiffer also will be responsible for ensuring that other investigators and project staff adhere to the UM IRB policies including: (1) all participants will understand, agree to and sign a written consent form before participating; (2) strict adherence to a participant's right to withdraw or refuse to answer questions will be maintained; (3) the assessments will be completely confidential and no names will be associated with the assessment data; (4) consent forms and identifying information will be kept separate from the actual participant data; (5) all identifying information (consents, tracking data) will be kept locked at all times and computer files will be saved with passwords; (6) participants will be informed in writing in the consent form how to contact the PI, the study coordinator, and UM IRB ofice with any questions and/or concerns.

Dr. Pfeiffer will directly supervise the data manager and the project coordinator and will be responsible for monitoring confidentiality procedures. Quality control and reliability of screening, baseline and follow-up assessments will be monitored by Dr. Pfeiffer throughout the trial via regular meetings and observation of the project coordinator conducting standardized assessments. Dr. Kim, the study biostatistician, will monitor the quality of the data files via supervision of the data manager.

A Data Safety and Monitoring Board will be created for this project. The DSMB will be composed of three faculty not involved with the project, who have expertise in randomized controlled trials, psychiatric emergencies, and statistical analysis. The DSMB will review the protocol before the study is initiated, with an emphasis on participant safety, and can recommend changes. Once the study begins, the committee will meet bi-annually throughout the study to review data on adverse events, recruitment, and adherence to the protocol.

Definitions for Adverse Events and Serious Adverse Events

- **Adverse Event (AE)** is any experience or abnormal finding that has taken place during the course of a research project and was harmful to the subject participating in the research, or increased the risks of harm from the research, or had an unfavorable impact on the risk/benefit ratio.

- **Serious Adverse Event (SAE)** is any adverse experience occurring at any dose or level of participation that results in any of the following outcomes: death, a life-threatening experience, hospitalization or prolongation or existing hospitalization, a persistent or significant disability or capacity, or a congenital anomaly or birth defect.

STATISTICAL ANALYSES PLAN

Sample Size. Based on our pilot study findings and those of other randomized controlled trials of psychosocial interventions that included suicide attempts as a primary outcome (91, 92, 109), we powered the study with the assumption that frequency of suicide attempts at 6 months will be 22% in the intervention arm vs. 36% in the control arm. We will enroll 490 patients in total, and assuming a 25% dropout rate, 368 (184 per group) will provide primary outcome data. The proposed sample size is expected to provide 84% power to detect the desired difference between the two groups based on a two-sided 0.05 level test. The estimate of effect is based on an assumption that the effect of PREVAIL will be similar to that reported in a trial of cognitive behavioral therapy for suicide among suicide attempters (N=120) where the re-attempt rate at 6 months was 25% among intervention recipients vs. 40% for controls. (91) Although there is no prior literature comparing the effects of peer support to psychotherapy on suicide-specific outcomes, two meta-analyses have concluded the effects of peer support on depression are similar to psychotherapy. (49, 51) Our estimate of effect is conservatively less than that found for brief psychodynamic interpersonal therapy, where in one trial (N=119) the self-harm rate at 6 months was 9% in the intervention arm compared to 28% for controls. (92) For the primary outcome of suicidal ideation according to the Beck Scale for Suicidal Ideation at 6 months and measures of hope and belongingness at 3 months (Aim 2), our sample of 368 will provide 90% power to detect a standardized effect size (Cohen's d) of .34. For the mediator analysis, the proposed sample size is expected to provide 80% power to detect a significant mediator assuming that the intervention explains about 2% of the variance of the mediator (corresponding to "small" effect based on Cohen's criteria), and about 7% of the variance of suicide ideation is explained by the mediator adjusting for the intervention effect, or vice versa based on joint significance testing for the mediation effect. (110)

Data Analysis

Baseline Analyses. We will examine the randomization process to determine if there were any between-group differences at baseline,

particularly in variables that are thought to be potentially associated with suicide attempts or ideation. T-tests or chi-square analyses, as appropriate, will be used to test for differences in the following baseline variables between study groups: age, severity of suicidal ideation prior to admission, burdensomeness, hope/hopelessness, and measures of belongingness. When there are significant differences in the distribution of these variables, they will be included as covariates in multivariable analyses. Balance is expected for sex and recent suicide attempt due to randomization by minimization.

Data Verification/Univariate Analyses: We will examine the distribution of all study variables for extreme values, missing data, variances, possible coding errors, skewness and whether or how to categorize skewed data. We will describe means (SD) for the continuous measures and frequencies of dichotomous outcomes by study group for each assessment time for the entire sample and for each of the study sites, separately.

Primary Outcome

Primary analyses will determine the effectiveness of PREVAIL in decreasing the risk of any suicide attempt and in decreasing suicidal ideation at 6 months post-randomization. Primary analytic cohort will be "intent to treat", such that all randomized participants will be included according to their original study arm assignment, regardless of whether they subsequently drop out of participation in the intervention. Secondary analyses will be conducted using a "per protocol" cohort where only participants who complete 6 or more peer encounters will be compared to those assigned to the EUC arm. We will evaluate predictors of compliance by defining 6 or more peer encounters as compliance and will also obtain complier average difference (a.k.a. complier average causal effect) assuming a balanced proportion of compliance between arms. The estimates of the three analytic cohorts (intent to treat, per protocol and compliance average difference) will provide more robust understanding of the effect of PREVAIL. (106)

Bivariate/Multivariate Analyses: To test for intervention effects at 6 months on the primary outcomes, a logistic regression model will be used for the dichotomous outcome of suicide attempt (as assessed by CSSR-S) and a multiple regression model will be used for suicidal ideation (as assessed by BSI). Each model will include the study arm as the primary predictor and will also include study site, gender, and prior suicide attempt, which were the balancing variables used in randomization. We will also adjust for additional covariates that show imbalances in baseline analyses. Outcomes at 6 months post-randomization will be our primary analysis in order to assess whether the intervention has lasting effects, but we will also assess immediate effects at 3 months using similar analytic approaches.

Effects over time: We will also assess the intervention effects over time using longitudinal data for both suicidal ideation and suicide attempt. For suicidal ideation, we will use a linear mixed-effects model with ideation assessed at baseline, 3 months, and 6 months. The model will allow the full use of all observed data despite missing outcomes at one or two assessment times and will give unbiased estimates as long as the missingness does not depend on the unobserved missing data (missing at random). For suicide attempt, we will use a generalized linear mixed-effects model with logit link for any suicide attempt in the prior 3 months as a binary response variable assessed at 3 and 6 months. These models will include participant as random intercepts to take into account the correlation of data within person over time. The models will allow estimation of the time-averaged effect of the intervention if the response to the intervention is immediate and lasting, or estimate the differential effect of the intervention over time if a significant interaction between time and intervention is found, such as different trends between the study groups. Whether we compare the time- averaged effects or the rates of changes between two groups, we will be guided by careful graphical exploration of the longitudinal data over time between the two groups. For example, if we find decreasing BSI scores from baseline to 3 months in both groups, but with a larger decrease in the intervention group, this will be modeled with interaction terms of time indicators for 3 and 6 months each by intervention group. The coefficients from the interaction terms of the BSI model will then allow testing for the between-arm difference in decrease in BSI from baseline to 3 months and baseline to 6 months. Similarly, the interaction term of the suicide attempt model will allow testing if the odds ratio of suicide attempt associated with the intervention differs from month 3 to month 6.

Secondary Outcomes

The effects of the intervention on the primary hypothesized mediators of hope (Hope Scale and Beck Hopelessness Scale) and belongingness (NIH Toolbox measures) will be assessed using multiple regression models based on a similar model as described under aim 1 for suicide ideation. Primary interest will be comparisons between treatment arms at 3 months (primary analysis) and 6 months (secondary analysis). Similar exploratory analyses will be used to assess intervention effects at 3 and 6 months on functioning, quality of life, depression, patient activation, healthcare utilization, and medication adherence.

Figure 4. Mediation Model of Intervention Effectiveness

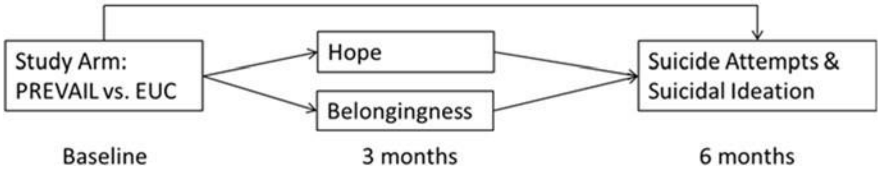

Mediation of Intervention Effects on Suicide Attempts and Suicidal Ideation: We will examine whether any improvements in the primary outcomes of suicide attempts or suicidal ideation are mediated through improvements in hope and belongingness in

primary exploratory analyses. The role of functioning, quality of life, depression, patient activation, healthcare utilization and medication adherence as mediators will be assessed in secondary exploratory analyses. To test whether a variable might be a mediator, we will first test the associations between the intervention arm and the mediator (described in above bivariate analyses), and then the mediator and the outcome of interest using bivariate analyses (e.g., hope at 3 months and suicidal ideation at 6 month). A variable will be considered a potential mediator if the variable at the 3-month assessment is associated with the intervention arm and with the outcome at 6 months as determined by a significance level of $p<.10$.

Potential mediators (at 3 months) will then be included as covariates in multivariate regression models predicting the primary outcomes at 6 months (Figure 4). If, as hypothesized, the intervention's effects on the primary outcomes are mediated through hope and belongingness (or other exploratory mediators), the variance in the 6-month outcomes explained by the intervention group will be less once the mediator variables are included in the models. Changes from baseline to 3 months will also be examined as potential mediators.

The intervention effects on any and number of readmissions to a psychiatric inpatient unit, emergency department visits, and outpatient mental health treatment visits (e.g., medication prescriber or psychotherapist) will be analyzed using generalized linear models with appropriate link function.

Approach to Missing Data. Our initial analyses will only use observed data. When data are missing for items within scales, we will use recommended imputation procedures rather than excluding participants from the analyses. For missing follow-up assessments, we will check if missingness is associated with any baseline variables including sites, and will include any such baseline variables in the models evaluating the intervention efect described above. In addition, as described under Efect Over time, the mixed- efects model is expected to provide an unbiased estimate of the intervention efect if missingness is at random. As a secondary analysis, we will also conduct an analysis that imputes missing data. We will likely have some missing baseline values in addition to missing follow-up assessments. Thus we will conduct multiple imputations based on chained equations (sequential regression multivariate imputation) to impute both baseline and follow-up data and will summarize the data using appropriate combination rules. (107, 108)

REGULATORY, ETHICAL AND STUDY OVERSIGHT CONSIDERATIONS

Informed Consent Process

Patients who appear to meet study inclusion and exclusion criteria based on review of medical records and consultation with the attending psychiatrist will be approached by the research assistant (RA) to describe the study, provide information about the randomization and 3 and 6-month assessments, as well as the risks, benefits, and limitations to confidentiality. Patients who state they are interested will be asked to complete a brief cognitive screener to assess whether they are substantially cognitively impaired and therefore unable to give informed consent. Patients who are not substantially cognitively impaired will complete a pre-screening consent form allowing study staff to administer the Beck Suicide Scale and items 4 and 5 of the Columbia Suicide Scale. Patents who have a score >5 on the Beck Suicide Scale will be eligible to participate and obtain informed consent. During the informed consent process, the study will be described and patients will have ample and repeated opportunities to ask questions.

We are requesting a waiver of consent for recruitment purposes. We will be screening medical records to determine patient eligibility. Due to the large number of patients admitted to the inpatient psychiatric unit, it would be dificult to obtain consent from each patient prior to screening. The risk to patients is minimal in order to allow staff to pre-screen medical records. Patients who are interested in participating will provide informed consent before study activities begin.

Telephonic Informed Consent Procedures During COVID-19 Pandemic: Subjects who enroll over the telephone due to COVID-19 restrictions will be provided with paper copies of consent forms for their records, and consent will be obtained verbally over the phone after reviewing the consent documents in detail and providing the patient ample opportunity to ask questions. Research staff will allow patients as much time as they wish to review the consent documents, and then they will read an informed consent script to the patient. Research staff will pause frequently and check in with the patient, asking if they understand what is being asked of them, and if they have any questions. For each signature line in the consent forms, research staff will ask the patient to provide verbal consent. Once consent is obtained, research staff will document the date that the subject consented in the study's de-identified database.

Confidentiality and Privacy. Every efort will be made to ensure that study data are always confidential, and never stored so that data can be linked to a particular person. Confidentiality will be explained to participants, including assurances that law enforcement personnel, and any others outside the research staff will not be informed of their responses. Limits to confidentiality will be explained verbally and in writing (e.g., physical or sexual abuse of minors, imminent threat to harm self or others).

Future Use of Stored Data. Data will be retained for study recordkeeping purposes until the end of analysis period and at least seven years from the end of the study period, in keeping with the relevant record-keeping guideline. There will not be any change in the conditions or arrangements for storage of research data/specimens during the retention period.

Data Handling & Recordkeeping

Data collection is the responsibility of the clinical trial staff at the site under the supervision of the site investigator. The investigator is responsible for ensuring the accuracy, completeness, legibility, and timeliness of the data reported.

All source documents should be completed in a neat, legible manner to ensure accurate interpretation of data. Preliminary eligibility data will be collected from participant's electronic medical records and entered into a 21 CFR Part 11- compliant Microsoft Access database provided by the University of Michigan. This data will also be stored in a Box folder approved for use with sensitive data by the University of Michigan. Any data that contains direct identifiers will be stored separately from deidentified data. Data backups will be performed regularly. This data will be linked by a coded identifier to direct identifiers (name, phone number, address, and medical record number) but stored separately. Data need to be linked to subjects' identities to help facilitate follow-up data collection procedures. Identifiers will be retained in accordance with the requirements of record-keeping for regulatory purposes. Clinical data will be entered directly from the electronic medical record. Outcome assessment data will be collected at baseline, 3 months, and 6 months using Qualtrics, an Application Server Provider (ASP) with a Software-as-a-Service (SaaS) platform for the creation and distribution of online surveys and related services. The platform records response data, and performs analysis and reporting. All services are located online. Qualtrics servers are protected by high-end firewall systems, and vulnerability scans are performed regularly. Complete penetration tests are performed yearly. All have quick fail over points with redundant hardware, and complete backups are performed nightly. Qualtrics uses Transport Layer Security (TLS) encryption, known as SSLv3.1, for all Internet transmitted data. Surveys may be protected with passwords. Qualtrics services are hosted by trusted third party data centers that are SSAE-16 SOC 1 Type II audited. All data at rest are encrypted, and data on deprecated hard drives are destroyed by U.S. DOD methods and delivered to third-party data destruction service.

Outcome assessments may be collected using paper surveys if internet access is unavailable during the time of the assessment, or based on a participant's needs or preferences. Paper copies of outcome assessment data will be linked to direct identifiers using a coded identifier. Deidentified data will always be stored separately from identifiable data. All paper records will be stored behind lock and key in locations that are only accessible to study staff who have been granted permission to use the data. If outcome assessments are conducted off-site, paper copies of outcome assessment data may be transported by study staff from the location of the outcome assessment to the secure storage site. Data will be transported directly back to the storage site by study staff, and will remain on our person at all times.

Peer research assistants will transport any handwritten clinical notes in a combination lockbox. These handwritten notes will be de-identified. Handwritten notes will be linked to direct identifiers using a coded identifier, and will stored separately from identifiable data behind lock and key in locations that are only accessible to study staff who have been grated permission to use the data. Peer research assistants may choose to keep electronic clinical notes using a Box folder that has been approved for storage of sensitive data by the University of Michigan. Electronic notes will be de-identified and linked to direct identifiers using a coded idientifier.

Per the Informed Consent protocol, research staff will collect a Suicide Safety Plan from participants assigned to the Peer Mentorship intervention upon discharge from the inpatient unit. These safety plans will be stored electronically in a Box folder approved by the University of Michigan for storage of sensitive data. Safety Plans contact direct identifiers and will therefore be stored separately from deidentified data.

Quality Assurance and Quality Control. Dr. Pfeiffer will directly supervise the data manager and the project coordinator and will be responsible for monitoring confidentiality procedures. Quality control and reliability of screening, baseline and follow-up assessments will be monitored by Dr. Pfeiffer throughout the trial via regular meetings and observation of the project coordinator conducting standardized assessments. Dr. Kim, the study biostatistician, will monitor the quality of the data files via supervision of the data manager.

Training

Dr. Pfeiffer will ensure that all relevant IRB policies, procedures and stipulations are being followed. Dr. Pfeiffer also will be responsible for ensuring that other investigators and project staff adhere to the UM IRB policies including: (1) all participants will understand, agree to and sign a written consent form before participating; (2) strict adherence to a participant's right to withdraw or refuse to answer questions will be maintained; (3) the assessments will be completely confidential and no names will be associated with the assessment data; (4) consent forms and identifying information will be kept separate from the actual participant data; (5) all identifying information (consents, tracking data) will be kept locked at all times and computer files will be saved with passwords; (6) participants will be informed in writing in the consent form how to contact the PI, the study coordinator, and UM IRB ofice with any questions and/or concerns.

Dr. Pfeiffer in cooperation with Co-Investigators will be responsible for providing training to all research staff working with participants with regard to procedures for managing potential clinical deterioration, suicidal crisis situations, and/or adverse events. This training will include information regarding evaluating warning signs of acute suicidal ideation, planning, or intent that could occur during contact with participants, and means of addressing such issues. Peer mentors will inquire about suicidal ideation at every encounter with participants, and participants complete self-report measures of suicidal ideation at baseline, 3-month, and 6-month

assessments conducted by RAs. In the case that a participant indicates any level of suicidal ideation or recent attempt during encounters with any study staff (including peer mentors), a suicide risk management protocol will be used. The protocol utilizes an algorithm of scripted risk assessment questions and "action steps" dependent on the participant responses. Based on the level of risk determined by the algorithm, study staff may perform one or more of the following: a) recommend the participant call or be transferred to the national suicide crisis hotline, the University of Michigan's Psychiatric Emergency Services (PES), or the nearest emergency department to immediately address suicide risk and determine whether emergency medical services or police should be activated to conduct a wellness check in the patient's home or community, b) notify the participant's outpatient treatment providers regarding a non-imminent increase in suicide risk, c) notify a designated on-call study team clinician (Pfeiffer, Ahmedani, or Abraham,) when the suicide risk management algorithm has been activated, and when any questions arise regarding risk management. The on-call study clinician will be available via pager or cell phone at all times when there is potential contact between study staff (including peer mentors) and participants.

Participants will be instructed on how to contact the national suicide crisis hotline, PES, or closest emergency department if they are experiencing a suicidal crisis. Peer mentors and study team members are not to further assess or manage suicide risk beyond the established protocol. In order to avoid participants contacting peer mentors in crisis when study clinicians are not available, peer specialists will be provided with cellular phones specifically for communication with study participants, and participants will be clearly informed that these cell phones will be of when not in use, and they are not monitored at all times. The voicemail messages on these phones will have instructions on how to contact crisis services. When Peer mentors will meet with participants outside of regular business hours; clinician back up will be prearranged. Written safety procedures for conducting community-based meetings include use of public locations, during daylight hours, and mandatory call-in procedures. In our prior research, using such procedures with similar populations, no incidents where staff safety concerns became an issue occurred during follow-up assessments. All safety concerns will be reported immediately to Dr. Pfeiffer.

Protocol Deviations. A protocol deviation is any noncompliance with the clinical trial protocol, International Conference on Harmonisation Good Clinical Practice (ICH GCP), or Manual of Procedures (MOP) requirements. The noncompliance may be either on the part of the participant, the investigator, or the study site staff. As a result of deviations, corrective actions are to be developed by the site and implemented promptly. It is the responsibility of the site investigator to use continuous vigilance to identify and report major protocol deviations that may adversely impact safety of participants, or impact integrity/validity of the data, and minor protocol deviations as part of a pattern or suggesting a systemic problem in study conduct that potentially places subjects or others at a greater risk of harm than was previously known or recognized, within seven (7) calendar days of becoming aware of the event or information.

Monitoring

The Principal Investigator, Dr. Pfeiffer, will be responsible for monitoring the data safety and quality. It should be noted that all research projects involving human participants at the University of Michigan, including the proposed, require approvals from the University of Michigan Medical School's Institutional Review Board (IRBMED). In addition, because of the sensitive nature of the data being collected, a Certificate of Confidentiality will be requested.

Dr. Pfeiffer will ensure that all relevant IRBMED policies, procedures, and stipulations are being followed. They will also will be responsible for ensuring that other investigators and project staff adhere to the UM IRBMED policies including: (1) All participants will understand, agree to and sign a written consent form before participating; (2) strict adherence to a participant's right to withdraw or refuse to answer questions will be maintained; (3) study assessments will be confidential and no names will be associated with the assessment data; (4) consent forms and identifying information will be kept separate from the actual participant data; (5) all identifying information (consents, tracking data) will be kept locked in a filing cabinet at all times and computer files will be saved with passwords; and (6) participants will be informed, in the consent form, how to contact the PI, the study coordinator, and the IRBMED ofice with any questions and/or concerns. Dr. Pfeiffer will be responsible for reporting any amendments to the UM IRBMED prior to implementation of any changes to the protocol. Dr. Pfeiffer in cooperation with Co-Investigators will be responsible for providing training to all research staff working with participants with regard to procedures for managing potential clinical deterioration, suicidal crisis situations, and/or adverse events. This training will include information regarding evaluating warning signs of acute suicidal ideation, planning, or intent that could occur during contact with participants, and means of addressing such issues. All safety concerns will be reported immediately to Dr. Pfeiffer.

An independent Data Safety Monitoring Board will be established that will review: (a) research protocols and plans for data and safety monitoring, (b) adverse events and unforeseen outcomes, (c) ethical issues related to recruitment activities, (d) risk management policies and activities, and (e) conduct intermediary data analyses, when appropriate. The board will be approved by the PIs, sponsoring Institute, and the University of Michigan Medical School IRB. DSMB members will be responsible for defining the events that will trigger additional "as needed" reviews and the criteria for retaining or removing patients from studies if they suffer from an adverse event. In addition, the DSMB will make recommendations to the study investigators about continuation or discontinuation of the project. These responsibilities are in line with the "NIH Policy for Data and Safety Monitoring" and further Guidance on Data and Safety Monitoring for Phase I and Phase II Trials." The DSMB members include scientists and clinicians with expertise in randomized controlled trials, psychiatric emergencies, and statistical analysis. The DSMB will meet in year 1 prior to the enrollment of the first

participant and then every 6 months until the trial concludes, and will be informed promptly of all serious adverse events. The DSMB will meet "as-needed" to discuss new study protocols and adverse events occurring between scheduled meetings.

The DSMB members are: Richard Balon, Lisa Brenner, and Timothy Hofer. Richard Balon, M.D. is the Associate Chair for Psychiatry in the Wayne State University School of Medicine, with expertise in psychopharmacology and anxiety and mood disorders. Lisa Brenner, Ph.D. is an Associate Professor for the University of Colorado Denver School of Medicine and the Director of the VA Rocky Mountain Mental Illness Research Education and Clinical Center (MIRECC). Dr. Brenner conducts suicide research with a focus on individuals with traumatic brain injury. Timothy Hofer, M.D. is a senior health services researcher and professor of internal medicine at the University of Michigan with expertise in quality, safety, and analytic methods. Dr. Hofer served on the DSMB for the R34 pilot study. All DSMB members are free of conflicts of interest with the proposed study. In keeping with the "NIH Policy for Data and Safety Monitoring", additional members will be included in the DSMB meeting as necessary to interpret the data and ensure patient safety. After each meeting, a summary report of discussion and resulting recommendations will be drafted and submitted to the local IRB and to NIH at each annual review.

The Data and Safety Monitoring Plan will be reviewed and approved by the Medical IRB at the University of Michigan. Monitoring is intended to protect subjects' rights and safety, and to ensure the integrity and quality of the data collected. The project coordinator will be responsible for creating a Regulatory Binder, which will contain the C.V. of all of the investigators, research staff, and safety monitoring committee (see below). All of the investigators and research staff involved in this trial will have completed the University of Michigan on-line educational programs on protection of human rights, Good Clinical Practice, and scientific ethics. Documentation of completion will be filed in the Regulatory Binder. The binder will also contain all communications to the IRB, including the initial application, study protocol, any amendments, annual IRB renewal, IRB approvals, and a summary of adverse events. It will be the responsibility of the project coordinator to maintain and update the Regulatory Binder.

The project coordinator will review consent forms, case report forms (CRF), and source data two weeks after the first subject is consented for screening, two weeks after the first subject is randomized, and then on at least a quarterly basis. Each review will be documented in the Monitoring Log and accompanied by a written Monitoring Report to be filed in the Regulatory Binder. The following materials will be monitored at each review:

a. Written informed consent for 100% of subjects
b. Inclusion/exclusion criteria for 100% of subjects
c. Adverse events and serious adverse events for 100% of subjects
d. All communications with the IRB, the sponsor, and other regulatory agencies
e. Any protocol violations
f. Completeness of data collection

In addition, all CRF for the first 3 randomized subjects will be verified completely, including completeness and consistency of questionnaires and interviews. Thereafter, every fifth subject will be verified for 100% of entries. If more than five errors are noted, then every case will be checked at the next review.

The PI will have the following responsibilities pertaining to data safety and monitoring during the course of the study:

a. Review of all adverse events as they occur
b. Review of all breaches of confidentiality if they occur
c. Communication with the IRB, the sponsor, or other regulatory agencies in the event of a serious adverse event or breach of confidentiality according to the specified guidelines
d. Periodic reviews of the literature regarding procedures used in the study to determine if any new information affects the safety or benefit-to-risk ratio of study participation. If such information emerges, then amendments documenting that information will be filed with the IRB.

C. Protocol Reference List

1. U.S. Department of Health and Human Services (HHS) Office of the Surgeon General and National Alliance for Suicide Prevention. National Strategy for Suicide Prevention: Goals and Objective for Action. Washington, DC. September 2012.
2. Curtin SC, Warner M, Hedegaard H. Increase in Suicide in the United States, 1999-2014. In: Centers for Disease Control and Prevention (CDC), editor. NCHS Data Brief No 2412016.
3. Van Orden KA, Witte TK, Cukrowicz KC, Braithwaite SR, Selby EA, Joiner TE, Jr. The interpersonal theory of suicide. Psychol Rev. 2010;117(2):575-600.
4. Zalsman G, Hawton K, Wasserman D, van Heeringen K, Arensman E, Sarchiapone M, et al. Suicide prevention strategies revisited: 10-year systematic review. Lancet Psychiatry. 2016;3(7):646-59.
5. Davidson L, Chinman M, Sells D, Rowe M. Peer support among adults with serious mental illness: a report from the field. Schizophr Bull. 2006;32(3):443-50.
6. Cook JA, Steigman P, Pickett S, Diehl S, Fox A, Shipley P, et al. Randomized controlled trial of peer-led recovery education using Building Recovery of Individual Dreams and Goals through Education and Support (BRIDGES). Schizophr Res. 2012;136(1-3):36-42.
7. Cook JA, Copeland ME, Jonikas JA, Hamilton MM, Razzano LA, Grey DD, et al. Results of a Randomized Controlled Trial of Mental Illness Self-management Using Wellness Recovery Action Planning. Schizophr Bull. 2012;38(4):881-91.
8. Dennis CL, Hodnett E, Kenton L, Weston J, Zupancic J, Stewart DE, et al. Effect of peer support on prevention of postnatal depression among high risk women: multisite randomised controlled trial. Br Med J. 2009;338.
9. Preyde M, Ardal F. Effectiveness of a parent "buddy" program for mothers of very preterm infants in a neonatal intensive care unit. Can Med Assoc J. 2003;168(8):969-73.
10. Robinson-Whelen S, Hughes RB, Taylor HB, Hall JW, Rehm LP. Depression self-management physical program for rural women with disabilities. Rehabil Psychol. 2007;52(3):254-62.
11. Lancaster GA, Dodd S, Williamson PR. Design and analysis of pilot studies: recommendations for good practice. J Eval Clin Pract. 2004;10(2):307-12.
12. Werner P. Director of Peer Support, Michigan Department of Health and Human Services. Personal interview via e-mail. Personal interview via e-mail. edDecember 13, 2017.
13. Institute of Medicine. Crossing the Quality Chasm: A New Health System for the 21st Century. The National Academies Press; 2001.
14. Curran GM, Bauer M, Mittman B, Pyne JM, Stetler C. Effectiveness-implementation hybrid designs: combining elements of clinical effectiveness and implementation research to enhance public health impact. Med Care. 2012;50(3):217-26.
15. Zero Suicide. What is Zero Suicide? : Education Development Center, Inc.; 2015 [updated January 18, 2017]; Available from: http://actionallianceforsuicideprevention.org/sites/actionallianceforsuicideprevention.org/files/zero_suicide_final6.pdf.
16. National Institute of Mental Health. Strategic Plan for Research. 2015.
17. Centers for Disease Control and Prevention (CDC). Web-based Injury Statistics Query and Reporting System (WISQARS). National Center for Injury Prevention and Control, CDC (producer). Available from http://www.cdc.gov/injury/wisqars/index.html. 2015.
18. Centers for Disease Control and Prevention (CDC). Suicide Facts at a Glance. 2015 [cited 2017 January 8]; Available from: https://www.cdc.gov/violenceprevention/pdf/suicide-datasheet-a.pdf.
19. Pitman A, Osborn D, King M, Erlangsen A. Effects of suicide bereavement on mental health and suicide risk. Lancet Psychiatry. 2014;1(1):86-94.
20. Fleegler EW, Lee LK, Monuteaux MC, Hemenway D, Mannix R. Firearm Legislation and Firearm-Related Fatalities in the United States. JAMA Intern Med. 2013;173(9):732-40.
21. Pirkis J, Spittal MJ, Cox G, Robinson J, Cheung YTD, Studdert D. The effectiveness of structural interventions at suicide hotspots: a meta-analysis. Int J Epidemiol. 2013;42(2):541-8.
22. Hawton K, Bergen H, Simkin S, Dodd S, Pocock P, Bernal W, et al. Long term effect of reduced pack sizes of paracetamol on poisoning deaths and liver transplant activity in England and Wales: interrupted time series analyses. BMJ-British Medical Journal. 2013;346.
23. Nordentoft M, Qin P, Helweg-Larsen K, Juel K. Restrictions in means for suicide: An effective tool in preventing suicide: The Danish experience. Suicide Life-Threat Behav. 2007;37(6):688-97.
24. LeFevre ML, Force USPST. Screening for Suicide Risk in Adolescents, Adults, and Older Adults in Primary Care: US Preventive Services Task Force Recommendation Statement. Ann Intern Med. 2014;160(10):719-+.
25. Isaac M, Elias B, Katz LY, Belik SL, Deane FP, Enns MW, et al. Gatekeeper Training as a Preventative Intervention for Suicide: A Systematic Review. Can J Psychiat-Rev Can Psychiat. 2009;54(4):260-8.
26. Britton PC, Bossarte RM, Thompson C, Kemp J, Conner KR. Influences on Call Outcomes among Veteran Callers to the National Veterans Crisis Line. Suicide Life-Threat Behav. 2013;43(5):494-502.
27. Mann JJ, Apter A, Bertolote J, Beautrais A, Currier D, Haas A, et al. Suicide prevention strategies – A systematic review. Jama-Journal of the American Medical Association. 2005;294(16):2064-74.
28. Luxton DD, June JD, Comtois KA. Can Postdischarge Follow-Up Contacts Prevent Suicide and Suicidal Behavior? A Review of the Evidence. Crisis. 2013;34(1):32-41.

29. Bertolote JM, Fleischmann A, De Leo D, Phillips MR, Botega NJ, Vijayakumar L, et al. Repetition of suicide attempts: data from emergency care settings in five culturally different low- and middle-income countries participating in the WHO SUPRE-MISS Study. Crisis. 2010;31(4):194-201.
30. Fleischmann A, Bertolote JM, Wasserman D, De Leo D, Bolhari J, Botega NJ, et al. Effectiveness of brief intervention and contact for suicide attempters: a randomixed controlled trial in five countries. Bull World Health Organ. 2008; 86(9):703-9.
31. Motto JA, Bostrom AG. A randomized controlled trial of postcrisis suicide prevention. Psychiatr Serv. 2001;52(6):828-33.
32. Cipriani A, Hawton K, Stockton S, Geddes JR. Lithium in the prevention of suicide in mood disorders: updated systematic review and meta-analysis. BMJ-British Medical Journal. 2013;346.
33. Meltzer HY, Alphs L, Green AI, Altamura AC, Anand R, Bertoldi A, et al. Clozapine treatment for suicidality in schizophrenia - International Suicide Prevention Trial (InterSePT). Archives of General Psychiatry. 2003;60(1):82-91.
34. Crawford MJ, Thomas O, Khan N, Kulinskaya E. Psychosocial interventions following self-harm - Systematic review of their efficacy in preventing suicide. Br J Psychiatry. 2007;190:11-7.
35. Mohr DC, Hart SL, Howard I, Julian L, Vella L, Catledge C, et al. Barriers to psychotherapy among depressed and nondepressed primary care patients. Ann Behav Med. 2006;32(3):254-8.
36. Pfeiffer PN, Bowersox N, Birgenheir D, Burgess J, Forman J, Valenstein M. Preferences and Barriers to Care Following Psychiatric Hospitalization at Two Veterans Affairs Medical Centers: A Mixed Methods Study. J Behav Health Serv Res. 2015.
37. Fullerton CA, Busch AB, Normand SL, McGuire TG, Epstein AM. Ten-year trends in quality of care and spending for depression: 1996 through 2005. Arch Gen Psychiatry. 2011;68(12):1218-26.
38. Pfeiffer PN, Ganoczy D, Zivin K, McCarthy JF, Valenstein M, Blow FC. Outpatient follow-up after psychiatric hospitalization for depression and later readmission and treatment adequacy. Psychiatr Serv. 2012;63(12):1239-42.
39. Valenstein M, Pfeiffer PN, Brandfon S, Walters H, Ganoczy D, Kim HM, et al. Augmenting Ongoing Depression Care With a Mutual Peer Support Intervention Versus Self-Help Materials Alone: A Randomized Trial. Psychiatr Serv. 2016;67(2):236-9. PMC5180417
40. Eisen SV, Schultz MR, Mueller LN, Degenhart C, Clark JA, Resnick SG, et al. Outcome of a Randomized Study of a Mental Health Peer Education and Support Group in the VA. Psychiatr Serv. 2012;63(12):1243-6.
41. Solomon P, Draine J. THE EFFICACY OF A CONSUMER CASE-MANAGEMENT TEAM - 2-YEAR OUTCOMES OF A RANDOMIZED TRIAL. J Ment Health Adm. 1995;22(2):135-46.
42. Joiner TE, Van Orden KA, Witte TK, Selby EA, Ribeiro JD, Lewis R, et al. Main Predictions of the Interpersonal-Psychological Theory of Suicidal Behavior: Empirical Tests in Two Samples of Young Adults. J Abnorm Psychol. 2009;118(3):634-46.
43. Christensen H, Batterham PJ, Soubelet A, Mackinnon AJ. A test of the Interpersonal Theory of Suicide in a large community-based cohort. J Affect Disord. 2013;144(3):225-34.
44. Pfeiffer PN, Brandfon S, Garcia E, Duffy S, Ganoczy D, Myra Kim H, et al. Predictors of suicidal ideation among depressed Veterans and the interpersonal theory of suicide. J Affect Disord. 2014;152- 154:277-81.
45. McMillan D, Gilbody S, Beresford E, Neilly L. Can we predict suicide and non-fatal self-harm with the Beck Hopelessness Scale? A meta-analysis. Psychol Med. 2007;37(6):769-78.
46. Stack S. Suicide: A 15-year review of the sociological literature part II: Modernization and social integration perspectives. Suicide Life-Threat Behav. 2000;30(2):163-76.
47. Stanley IH, Hom MA, Rogers ML, Hagan CR, Joiner TE. Understanding suicide among older adults: a review of psychological and sociological theories of suicide. Aging Ment Health. 2016;20(2):113-22.
48. Hogan BE, Linden W, Najarian B. Social support interventions: do they work? Clin Psychol Rev. 2002;22(3):383-442.
49. Pfeiffer PN, Heisler M, Piette JD, Rogers MAM, Valenstein M. Efficacy of peer support interventions for depression: a meta-analysis. General Hospital Psychiatry. 2011;33(1):29-36. PMC3052992
50. Fuhr DC, Salisbury TT, De Silva MJ, Atif N, van Ginneken N, Rahman A, et al. Effectiveness of peerdelivered interventions for severe mental illness and depression on clinical and psychosocial outcomes: a systematic review and meta-analysis. Soc Psychiatry Psychiatr Epidemiol. 2014;49(11):1691-702.
51. Bryan AEB, Arkowitz H. Meta-Analysis of the Effects of Peer-Administered Psychosocial Interventions on Symptoms of Depression. American Journal of Community Psychology. 2015;55(3-4):455-71.
52. Salvatore T. Peer specialists can prevent suicides. Behav Healthc. 2010;30(9):31-2.
53. Chinman M, Shoai R, Cohen A. Using organizational change strategies to guide peer support technician implementation in the Veterans Administration. Psychiatr Rehabil J. 2010;33(4):269-77.
54. Grant EA, Reinhart C, Wituk S, Meissen G. An Examination of the Integration of Certified Peer Specialists into Community Mental Health Centers. Community Mental Health Journal. 2012;48(4):477-81.
55. Open Minds. OPEN MINDS Market Intelligence Report. 2014 [January 10, 2017]; Available from: https://www.openminds.com/intelligence-report/states-reimburse-collaborative-documentation-peer-supportspecialists/.
56. Shattell MM, Harris B, Beavers J, Tomlinson SK, Prasek L, Geevarghese S, et al. A Recovery-Oriented Alternative to Hospital Emergency Departments for Persons in Emotional Distress: "The Living Room". Issues Ment Health Nurs. 2014;35(1):4-12.

57. Greenfield TK, Stoneking BC, Humphreys K, Sundby E, Bond J. A randomized trial of a mental health consumer-managed alternative to civil commitment for acute psychiatric crisis. American Journal of Community Psychology. 2008;42(1-2):135-44.
58. Zero Suicide. The Role of Peer Support Services in Caring for Those at Risk of Suicide [Powerpoint slides]. 2015; Available from: http://zerosuicide.sprc.org/sites/zerosuicide.actionallianceforsuicideprevention.org/files/The%20Role%20of%20Peer%20Support%20Services%20slides%206-2-15.pdf.
59. Chinman M, Lucksted A, Gresen R, Davis M, Losonczy M, Sussner B, et al. Early experiences of employing consumer-providers in the VA. Psychiatr Serv. 2008;59(11):1315-21.
60. Dixon L, Krauss N, Lehman A. Consumers as service providers: the promise and challenge. Community Ment Health J. 1994;30(6):615-25; discussion 27-34.
61. Damschroder LJ, Aron DC, Keith RE, Kirsh SR, Alexander JA, Lowery JC. Fostering implementation of health services research findings into practice: a consolidated framework for advancing implementation science. Implement Sci. 2009;4.
62. Carroll KM, Nich C, Sifry RL, Nuro KF, Frankforter TL, Ball SA, et al. A general system for evaluating therapist adherence and competence in psychotherapy research in the addictions. Drug Alcohol Depend. 2000;57(3):225-38.
63. Posner K, Brown GK, Stanley B, Brent DA, Yershova KV, Oquendo MA, et al. The Columbia-Suicide Severity Rating Scale: initial validity and internal consistency findings from three multisite studies with adolescents and adults. Am J Psychiatry. 2011;168(12):1266-77.
64. Beck AT, Steer RA, Ranieri WF. Scale for Suicide Ideation: psychometric properties of a self-report version. J Clin Psychol. 1988;44(4):499-505.
65. Cyranowski JM, Zill N, Bode R, Butt Z, Kelly MA, Pilkonis PA, et al. Assessing social support, companionship, and distress: National Institute of Health (NIH) Toolbox Adult Social Relationship Scales. Health Psychol. 2013;32(3):293-301.
66. Beck AT, Steer RA. Manual for the Beck Hopelessness Scale. San Antonio, TX: Psychological Corp; 1988.
67. Snyder CR, Sympson SC, Ybasco FC, Borders TF, Babyak MA, Higgins RL. Development and validation of the State Hope Scale. J Pers Soc Psychol. 1996;70(2):321-35.
68. Pfeiffer PN, Valenstein M, Ganoczy D, Henry J, Dobscha SK, Piette JD. Pilot study of enhanced social support with automated telephone monitoring after psychiatric hospitalization for depression. Soc Psychiatry Psychiatr Epidemiol. 2016.
69. King CA, Kramer A, Preuss L, Kerr DCR, Weisse L, Venkataraman S. Youth-nominated support team for suicidal adolescents (Version 1): A randomized controlled trial. J Consult Clin Psychol. 2006;74(1):199-206.
70. Pearson JL, Stanley B, King CA, Fisher CB. Intervention research with persons at high risk for suicidality: Safety and ethical considerations. J Clin Psychiatry. 2001;62:17-26.
71. King CA, Kramer AC. Intervention Research with Youths at Elevated Risk for Suicide: Meeting the Ethical and Regulatory Challenges of Informed Consent and Assent. Suicide Life-Threat Behav. 2008;38(5):486-97.
72. Ahmedani BK, Simon GE, Stewart C, Beck A, Waitzfelder BE, Rossom R, et al. Health care contacts in the year before suicide death. J Gen Intern Med. 2014;29(6):870-7.
73. Pfeiffer PN, Blow AJ, Miller E, Forman J, Dalack GW, Valenstein M. Peers and peer-based interventions in supporting reintegration and mental health among National Guard soldiers: a qualitative study. Military Medicine. 2012;177(12):1471-6.
74. Krein SL, Damschroder LJ, Kowalski CP, Forman J, Hofer TP, Saint S. The influence of organizational context on quality improvement and patient safety efforts in infection prevention: a multi-center qualitative study. Soc Sci Med. 2010;71(9):1692-701.
75. Damschroder LJ, Banaszak-Holl J, Kowalski CP, Forman J, Saint S, Krein SL. The role of the champion in infection prevention: results from a multisite qualitative study. Qual Saf Health Care. 2009;18(6):434-40.
76. Forman J, Creswell JW, Damschroder L, Kowalski CP, Krein SL. Qualitative research methods: key features and insights gained from use in infection prevention research. Am J Infect Control. 2008;36(10):764- 71.
77. Troister T, Links PS, Cutcliffe J. Review of predictors of suicide within 1 year of discharge from a psychiatric hospital. Curr Psychiatry Rep. 2008;10(1):60-5.
78. Milian M, Leiherr AM, Straten G, Muller S, Leyhe T, Eschweiler GW. The Mini-Cog versus the Mini- Mental State Examination and the Clock Drawing Test in daily clinical practice: screening value in a German Memory Clinic. Int Psychogeriatr. 2012;24(5):766-74.
79. Borson S, Scanlan J, Brush M, Vitaliano P, Dokmak A. The Mini-Cog: A cognitive 'vital signs' measure for dementia screening in multi-lingual elderly. Int J Geriatr Psychiatr. 2000;15(11):1021-7.
80. Altman DG, Bland JM. Treatment allocation by minimisation. BMJ. 2005;330(7495):843.
81. Zhang J, McKeown RE, Hussey JR, Thompson SJ, Woods JR. Gender differences in risk factors for attempted suicide among young adults: findings from the Third National Health and Nutrition Examination Survey. Ann Epidemiol. 2005;15(2):167-74.
82. Freedland KE, Mohr DC, Davidson KW, Schwartz JE. Usual and Unusual Care: Existing Practice Control Groups in Randomized Controlled Trials of Behavioral Interventions. Psychosom Med. 2011;73(4):323- 35.
83. Luxton DD, Thomas EK, Chipps J, Relova RM, Brown D, McLay R, et al. Caring letters for suicide prevention: implementation of a multi-site randomized clinical trial in the U.S. military and Veteran Affairs healthcare systems. Contemp Clin Trials. 2014;37(2):252-60.

84. Cedereke M, Monti K, Ojehagen A. Telephone contact with patients in the year after a suicide attempt: does it affect treatment attendance and outcome? A randomised controlled study. Eur Psychiat. 2002;17(2):82-91.
85. Vaiva G, Ducrocq F, Meyer P, Mathieu D, Philippe A, Libersa C, et al. Effect of telephone contact on further suicide attempts in patients discharged from an emergency department: randomised controlled study. Br Med J. 2006;332(7552):1241-4.
86. Knesper D, American Association of Suicidology, & Suicide Prevention Resource Center. Continuity of care for suicide prevention and research: Suicide attempts and suicide deaths subsequent to discharge from the emergency department or psychiatry inpatient unit. Newton, MA: Education Development Center, Inc.2010.
87. Booth BM, Kirchner J, Fortney J, Ross R, Rost K. Rural at-risk drinkers: correlates and one-year use of alcoholism treatment services. J Stud Alcohol. 2000;61(2):267-77.
88. Mundt JC, Greist JH, Jefferson JW, Federico M, Mann JJ, Posner K. Prediction of suicidal behavior in clinical research by lifetime suicidal ideation and behavior ascertained by the electronic Columbia-SuicideSeverity Rating Scale. J Clin Psychiatry. 2013;74(9):887-93.
89. Beck AT, Steer RA, Garbin MG. PSYCHOMETRIC PROPERTIES OF THE BECK DEPRESSION INVENTORY - 25 YEARS OF EVALUATION. Clin Psychol Rev. 1988;8(1):77-100.
90. Beck AT, Brown GK, Steer RA, Dahlsgaard KK, Grisham JR. Suicide ideation at its worst point: A predictor of eventual suicide in psychiatric outpatients. Suicide Life-Threat Behav. 1999;29(1):1-9.
91. Brown GK, Ten Have T, Henriques GR, Xie SX, Hollander JE, Beck AT. Cognitive therapy for the prevention of suicide attempts: a randomized controlled trial. JAMA. 2005;294(5):563-70.
92. Guthrie E, Kapur N, Mackway-Jones K, Chew-Graham C, Moorey J, Mendel E, et al. Randomised controlled trial of brief psychological intervention after deliberate self poisoning. Br Med J. 2001;323(7305):135-7.
93. Beck AT, Brown G, Berchick RJ, Stewart BL, Steer RA. Relationship between hopelessness and ultimate suicide: a replication with psychiatric outpatients. Am J Psychiatry. 1990;147(2):190-5.
94. Cook JA, Copeland ME, Floyd CB, Jonikas JA, Hamilton MM, Razzano L, et al. A randomized controlled trial of effects of Wellness Recovery Action Planning on depression, anxiety, and recovery. Psychiatr Serv. 2012;63(6):541-7.
95. Van Orden KA, Cukrowicz KC, Witte TK, Joiner TE. Thwarted belongingness and perceived burdensomeness: construct validity and psychometric properties of the Interpersonal Needs Questionnaire. Psychol Assess. 2012;24(1):197-215.
96. Endicott J, Nee J, Harrison W, Blumenthal R. Quality of Life Enjoyment and Satisfaction Questionnaire: a new measure. Psychopharmacol Bull. 1993;29(2):321-6.
97. Wisniewski SRSR, Rush AJAJ, Bryan CC, Shelton RR, Trivedi MHMH, Marcus SS, et al. Comparison of quality of life measures in a depressed population. J Nerv Ment Dis. 2007;195(3):219-25.
98. Rush AJ, Trivedi MH, Wisniewski SR, Nierenberg AA, Stewart JW, Warden D, et al. Acute and longerterm outcomes in depressed outpatients requiring one or several treatment steps: a STAR*D report. Am J Psychiatry. 2006;163(11):1905-17.
99. Ware JE, Kosinski M, Keller SD. A 12-item short-form health survey - Construction of scales and preliminary tests of reliability and validity. Med Care. 1996;34(3):220-33.
100. Jenkinson C, Layte R, Jenkinson D, Lawrence K, Petersen S, Paice C, et al. A shorter form health survey: Can the SF-12 replicate results from the SF-36 in longitudinal studies? Journal of Public Health Medicine. 1997;19(2):179-86.
101. Green CA, Perrin NA, Polen MR, Leo MC, Hibbard JH, Tusler M. Development of the Patient Activation Measure for Mental Health. Adm Policy Ment Health. 2010;37(4):327-33.
102. Salyers MP, Matthias MS, Spann CL, Lydick JM, Rollins AL, Frankel RM. The role of patient activation in psychiatric visits. Psychiatr Serv. 2009;60(11):1535-9.
103. Kroenke K, Spitzer RL. The PHQ-9: A new depression diagnostic and severity measure. Psychiatr Ann. 2002;32(9):509-15.
104. Kroenke K, Spitzer RL, Williams JBW. The PHQ-9 - Validity of a brief depression severity measure. Journal of General Internal Medicine. 2001;16(9):606-13.
105. Lu M, Safren SA, Skolnik PR, Rogers WH, Coady W, Hardy H, et al. Optimal recall period and response task for self-reported HIV medication adherence. AIDS Behav. 2008;12(1):86-94.
106. Sheiner LB. Is intent-to-treat analysis always (ever) enough? British Journal of Clinical Pharmacology. 2002;54(2):203-11.
107. Raghunathan TE, Lepkowski JM, Van Hoewyk J, Solenberger P. A Multivariate Technique for Multiple Imputing Missing Values Using a Sequence of Regression Models. Survey Methodology. 2001;27(1):85-95.
108. Rubin DB. Multiple Imputation for Nonresponse in Surveys. New York: Wiley; 1987.
109. Rudd MD, Bryan CJ, Wertenberger EG, Peterson AL, Young-McCaughan S, Mintz J, et al. Brief cognitive-behavioral therapy effects on post-treatment suicide attempts in a military sample: results of a randomized clinical trial with 2-year follow-up. Am J Psychiatry. 2015;172(5):441-9.
110. Fritz MS, Mackinnon DP. Required sample size to detect the mediated effect. Psychol Sci. 2007;18(3):233-9.
111. Kirk MA, Kelley C, Yankey N, Birken SA, Abadie B, Damschroder L. A systematic review of the use of the Consolidated Framework for Implementation Research. Implement Sci. 2016;11.
112. Patton MQ. Qualitative Research and Evaluation Methods, 3rd Edition. Thousand Oaks, CA: Sage Publications; 2002.
113. Sandelowski M, Barroso J. Writing the proposal for a qualitative research methodology project. Qual Health Res. 2003;13(6):781-820.

114. Snyder, C. R., Sympson, S. C., Ybasco, F. C., Borders, T. F., Babyak, M. A., & Higgins, R. L. (1996). Development and validation of the State Hope Scale. Journal of personality and social psychology, 70(2), 321.
115. Steger, M. F., Frazier, P., Oishi, S., & Kaler, M. (2006). The meaning in life questionnaire: Assessing the presence of and search for meaning in life. Journal of counseling psychology, 53(1), 80.
116. Devilly, G. J., & Borkovec, T. D. (2000). Psychometric properties of the credibility/expectancy questionnaire. Journal of behavior therapy and experimental psychiatry, 31(2), 73-86.
117. Czyz, E. K., Bohnert, A. S., King, C. A., Price, A. M., Kleinberg, F., & Ilgen, M. A. (2014). Self-Efficacy to Avoid Suicidal Action: Factor Structure and Convergent Validity among Adults in Substance Use Disorder Treatment. Suicide and Life-Threatening Behavior, 44(6), 698-709.
118. Zimet, G. D., Dahlem, N. W., Zimet, S. G., & Farley, G. K. (1988). The multidimensional scale of perceived social support. Journal of personality assessment, 52(1), 30-41.
119. Callahan CM, Unverzagt FW, Hui SL, Perkins AJ, Hendrie HC. Six-item screener to identify cognitive impairment among potential subjects for clinical research. Medical care. 2002 Sep 1;40(9):771-81.

D. Summary of Protocol Amendments

Date	Amendment Description
September 2018	• Refined exclusion criterion for concomitant peer support to only exclude patients from study participant due to outside peer support if they are receiving or plan to receive individual peer mentorship from a Certified Peer Support Specialist or plan to meet with a suicide prevention-focused support group at least biweekly<br>• Specified that patients with a legal guardian are excluded from study participation (unable to consent)<br>• Added recruitment procedures for when we are unable to offer participants a choice of which peer mentor they wish to work with. Research staff will offer patients a short, written description of the available peer during recruitment (prior to consent), to avoid enrolling a participant who is not comfortable working with the available peer<br>• Collect a voluntary Request for Outside Records from participants who report an ER visit or hospitalization outside Michigan Medicine or HFHS.
April 2019	• New retention protocols: (1) To bypass filtering of Facebook messages when using social media to contact hard-to-reach participants, we will send a Facebook friend request (from a private study account) as well as a private message. Participants are informed of this during enrollment and can opt out. (2) We will utilize a public records search service to find new contact information for hard-to-reach participants. (3) We will mail 2 seasonal greeting cards over 6 months to participants as an additional reminder for their next follow-up assessment.<br>• Refined fidelity rating instrument for evaluating audio recordings of peer mentorship sessions for improved clarity and interrater reliability.<br>• Adopted a protocol for assessing whether to continue an assessment with an intoxicated participant.<br>• Increased payments for the 3- and 6-month outcome assessments from $25 to $35 to improve retention, and added potential for some participants to receive an additional $20 incentive who miss scheduled follow-up visits or have not responded to contact attempts. Participant may be offered an additional $10 for both the 3- and 6-month assessments, or an additional $20 at 6-month time point if they did not receive the additional $10 for the 3-month assessment.<br>• Research staff may consult with a designated nurse or resident to determine if a patient is appropriate for recruitment when the attending provider is not available to consult.
July 2019	• New exclusion criterion: exclude participants with homicidal or violent ideation or behavior at the time of admission.<br>• We will mail a post-enrollment thank you card to new participants to improve retention.<br>• Added a question to the 6-Month Intervention Arm Participant Debrief survey that asks participants for feedback about the 3-month duration of the peer mentorship intervention.<br>• Shortened the Participant Early Withdrawal Debrief Survey to 2 items, in order to increase the response rate, as no participants were completing these surveys.<br>• Added the Mini Mental State Exam (MMSE) as an allowable alternative to the Mini Cog as a cognitive screening measure for patients who do not know how to read an analog clock.
February 2020	• Changed the exclusion of participants with Homicidal ideation (HI) to past 6-months: Previously, patients were excluded from participant if they had documentation of homicidal or violent ideation at the time of their admission. We extended the exclusion window for violent / homicidal ideation to include the past 6 months prior to inpatient admission.<br>• Extended the 6-month follow-up window: On the recommendation of study co-investigators, we amended the window for 6-month follow-ups to allow for data collection immediate after the 3-month window ends for very difficult-to-reach participants, to eliminate any gap between follow-up assessment windows.
March 2020	Amended study intervention protocol to include policies for therapeutic boundaries and description of remedial actions the study team will take if these policies are violated.
June 2020	Added protocol for resuming recruitment (via telephone) from UM inpatient psychiatry unit.
January 2021	Added protocols for resuming in-person recruitment (with COVID-19 infection prevention precautions)
March 2021	At the request of IRBMED, amended protocol to (1) clarify protocols for how peers are supported during work with high-risk participants, including protocols for weekly supervision and debriefing after serious adverse events; (2) add a protocol for biannual, one-on-one check-ins between the PI and peer mentors, and (3) add a protocol for checking in with participants halfway through the intervention period .
September 2021	Revised study sites; added Henry Ford Macomb Hospital
January 2022	Revised inclusion/exclusion criteria: previously, participants had to live within 50 miles of a study peer specialist. Given the change to remote intervention delivery following the COVID-19 pandemic, this restriction is no longer necessary. We changed this criterion to only exclude patients if they live outside the state of Michigan.
