# Supplementary material for: Peer Support Intervention for Suicide Prevention Among High-Risk Adults in Michigan: A Randomized Clinical Trial
Source: JAMA Netw Open. 2025 May 28;8(5):e2510808. doi: 10.1001/jamanetworkopen.2025.10808 (PMC12120652; doi:10.1001/jamanetworkopen.2025.10808)
Supplement: Supplement 2. — eTable 1. Full Demographic Characteristics, Including Missing Values eTable 2. Complete Baseline and Follow-Up Measure by Study Arm and Engager Status eTable 3. Missing Values by Study Arm and Engager Status eMethods. Generalized Linear Mixed-Effects Model Methods and Results eTable 4. Generalized Linear Mixed-Effects Models of Primary Outcomes Across All Assessment Time Points [file jamanetwopen-e2510808-s002.pdf]

Supplemental Online Content

Pfeiffer PN, Abraham KM, Lapidos A, et al. Peer support intervention for suicide prevention among high-risk adults in Michigan. *JAMA Netw Open*. 2025;8(5):e2510808. doi:10.1001/jamanetworkopen.2025.10808

eTable 1. Full Demographic Characteristics, Including Missing Values

eTable 2. Complete Baseline and Follow-Up Measure by Study Arm and Engager Status

eTable 3. Missing Values by Study Arm and Engager Status

eMethods. Generalized Linear Mixed-Effects Model Methods and Results

eTable 4. Generalized Linear Mixed-Effects Models of Primary Outcomes Across All Assessment Time Points

This supplemental material has been provided by the authors to give readers additional information about their work.

© 2025 Pfeiffer PN et al. *JAMA Network Open*.

eTable 1. Full Demographic Characteristics, Including Missing Values

Characteristic	PREVAIL (N=229)	EUC (N=226)
	Participants, No. (%)	Participants, No. (%)
Gender		
Female	134 (58.5)	139 (61.5)
Male	78 (34.1)	76 (33.6)
Transgender/Non-binary/Not listed	17 (7.4)	11 (4.9)
Missing	0 (0.0)	0 (0.0)
Race and Ethnicity*		
Black, Afro-Caribbean, or African American	45 (19.7)	46 (20.4)
East Asian or Asian American	9 (3.9)	7 (3.1)
Hawaiian/Pacific Islander	2 (0.9)	0 (0)
Latino or Hispanic American	8 (3.5)	15 (6.6)
Middle Eastern or Arab American	0 (0)	6 (2.7)
Native American or Alaskan Native	6 (2.6)	7 (3.1)
Non-Hispanic White or Euro-American	172 (75.1)	161 (71.2)
South Asian or Indian American	4 (1.7)	4 (1.8)
"Other, Please Describe:"	4 (1.7)	4 (1.8)
Missing	1 (0.4)	0 (0.0)
Age, mean (SD), y	32.4 (14.0)	31.6 (13.5)
Occupation		
Homemaker/caregiver	11 (4.8)	15 (6.6)
Retired	10 (4.4)	9 (4.0)
Working full or part-time	115 (50.2)	123 (54.4)
Volunteer	3 (1.3)	5 (2.2)
Unemployed or on disability	58 (25.3)	57 (25.2)
Student	67 (29.3)	62 (27.4)
Missing	2 (0.9)	1 (0.4)
Education, %		
High school or less	73 (31.9)	63 (27.9)
Some college	98 (42.8)	98 (43.4)
College graduate or advanced degree	58 (25.3)	65 (28.8)
Missing	0 (0.0)	0 (0.0)
Marital Status, %		
Married	45 (19.7)	35 (15.5)
Divorced/separated	30 (13.1)	29 (12.8)
Widowed	2 (.9)	9 (4.0)
Never Married	137 (59.8)	134 (59.3)
Living together, not married	15 (6.6)	19 (8.4)
Missing	0 (0.0)	0 (0.0)
Annual income, %		

© 2025 Pfeiffer PN et al. *JAMA Network Open.*

Under $5,000	56 (24.5)	60 (26.5)
$5,000 to $14,999	48 (21.0)	48 (21.2)
15,000 to $24,999	41 (17.9)	34 (15.0)
$25,000 to $49,999	43 (18.8)	44 (19.4)
$50,000 or more	35 (15.2)	36 (15.9)
Missing	6 (2.6)	4 (1.8)
Living situation, %		
Lives with spouse or partner	65 (28.4)	64 (28.3)
Lives with parents	61 (26.6)	67 (29.7)
Lives with children under age 18	39 (17.0)	43 (19.0)
Lives with other relatives or friends	61 (26.6)	47 (20.8)
Lives with others, not relatives or friends	7 (3.1)	16 (7.1)
Lives alone	32 (14.0)	41 (18.1)
Homeless or between housing	22 (9.6)	14 (6.2)
Missing	0 (0.0)	0 (0.0)

© 2025 Pfeiffer PN et al. *JAMA Network Open*.

eTable 2. Complete Baseline and Follow-Up Measures by Study Arm and Engager Status[a]

Outcome Measures	Baseline PREVAIL (N=229)	Baseline EUC (N=226)	3 Months PREVAIL ITT (N=149)	3 Months Engagers (N=108)	3 Months EUC (N=158)	6 Months PREVAIL ITT (N=141)	6 Months Engagers (N=103)	6 Months EUC (N=142)
Beck Scale for SI	22.0 (7.9)	22.7 (7.0)	4.1 (6.6)	3.9 (6.1)	4.4 (6.8)	4.9 (7.3)	4.0 (6.4)	4.3 (6.6)
Beck Scale for SI – Worst Point	--	--	10.7 (10.5)	10.8 (10.4)	10.5 (9.9)	7.8 (9.1)	7.5 (8.8)	8.7 (9.3)
Beck Hopelessness Scale	8.4 (5.9)[b]	9.8 (6.1)	6.3 (5.6)	5.8 (5.5)	6.5 (5.3)	6.2 (5.8)	5.8 (5.4)	6.6 (5.7)
INQ Perceived Burdensomeness	16.0 (8.9)	16.2 (8.5)	12.0 (8.2)	11.6 (8.4)	11.9 (7.7)	12.8 (8.6)	12.4 (8.2)	11.9 (7.4)
INQ Thwarted Belongingness	22.3 (7.1)	23.0 (6.9)	18.9 (7.8)	18.3 (7.9)	19.5 (8.0)	19.0 (8.4)	18.6 (8.1)	20.2 (8.2)
Hope Scale	27.3 (10.9)	25.6 (10.3)	32.1 (10.4)	33.4 (9.8)[b]	30.6 (9.8)	32.0 (11.5)	33.1 (10.8)	31.3 (10.4)
NIH Emotional Support	27.5 (8.7)	27.2 (7.9)	31.3 (7.9)	32.1 (7.2)	31.0 (7.3)	31.0 (8.0)	31.9 (7.2)	30.6 (7.6)
NIH Instrumental Support	25.8 (9.8)	26.1 (9.4)	28.5 (9.7)	29.0 (9.6)	28.9 (9.1)	27.5 (9.7)	27.5 (9.7)	28.7 (9.1)
NIH Friendship	21.4 (8.1)	22.6 (8.3)	24.3 (9.0)	24.7 (9.1)	24.7 (8.4)	24.8 (8.8)	25.0 (8.7)	24.4 (8.9)
NIH Loneliness	17.7 (4.7)	17.9 (4.7)	15.2 (6.0)	15.0 (6.1)	15.0 (5.3)	14.7 (5.7)	14.4 (5.5)	15.3 (5.7)
NIH Rejection	22.4 (8.1)	22.8 (7.7)	18.4 (8.5)	17.9 (8.2)	19.1 (8.2)	18.6 (8.7)	17.9 (8.4)	18.7 (7.8)
Suicide self-efficacy	31.8 (14.1)	29.3 (13.6)	36.1 (13.4)	36.5 (13.6)	34.6 (13.0)	37.1 (11.7)	37.6 (11.2)	35.5 (12.0)
Q-LES-Q-SF	39.2 (10.3)	38.8 (9.8)	46.4 (11.4)	47.4 (11.6)	45.8 (10.2)	46.1 (12.0)	47.6 (11.0)	46.5 (10.8)
SF-12 Physical health subscale	51.7 (12.2)	51.4 (13.2)	48.6 (13.3)	49.0 (13.2)	48.9 (12.7)	49.2 (12.4)	50.3 (12.2)	48.2 (13.7)
SF-12 Mental health subscale	23.5 (10.7)	23.8 (11.0)	36.0 (12.4)	36.9 (12.3)	36.3 (11.8)	35.6 (13.5)	36.7 (13.7)	36.4 (12.6)
MSPSS total mean	4.7 (1.6)	4.6 (1.4)	5.1 (1.6)	5.2 (1.6)	4.9 (1.4)	5.0 (1.7)	5.2 (1.6)	5.0 (1.3)
MSPSS significant other subscale	5.1 (1.9)	4.9 (1.9)	5.4 (1.9)	5.5 (1.9)	5.3 (1.8)	5.4 (1.9)	5.6 (1.8)	5.5 (1.6)
MSPSS family subscale	4.4 (2.0)[b]	4.1 (1.8)	4.8 (2.1)	4.8 (2.1)	4.7 (1.9)	4.6 (2.1)	4.9 (2.0)	4.6 (1.8)
MSPSS friend subscale	4.6 (1.9)	4.8 (1.7)	5.0 (1.8)	5.2 (1.8)	4.8 (1.8)	5.0 (1.9)	5.2 (1.8)	5.0 (1.8)
Patient Health Questionnaire (PHQ-9)	18.3 (6.6)	18.3 (5.8)	10.8 (6.9)	10.2 (6.8)	10.7 (6.9)	11.6 (7.3)	11.0 (7.1)	10.8 (6.7)
MLQ – Presence	18.4 (8.4)	17.3 (8.1)	22.0 (8.9)	22.4 (8.7)	20.5 (8.5)	21.5 (8.7)	21.8 (8.7)	19.9 (8.5)
MLQ – Search	25.8 (7.7)	25.5 (7.1)	25.8 (7.4)	25.5 (7.7)	25.1 (7.0)	25.7 (7.3)	25.6 (7.4)	25.0 (7.0)
Any Inpatient Psychiatry Admission[c]	49.6%	46.9%	18.1%	20.0%	13.0%	10.5%	11.3%	10.5%
Any Mental Health Service Use[c]	85.5%	80.5%	79.0%	84.0%	81.5%	64.2%	62.9%	73.7%

Abbreviations: ITT = intent to treat sample; EUC = enhanced usual care; SI = Suicidal ideation; INQ = Interpersonal Needs Questionnaire; NIH = National Institutes of Health; Q-LES-Q = Quality of Life Enjoyment and Satisfaction Questionnaire Short Form; SF-12 = 12-item Short Form; MDPSS = Multidimensional Scale of Perceived Social Support; MLQ = Meaning in Life Questionnaire

a. Engagers = intervention participants who completed ≥ 6 sessions with a peer specialist.
b. P<.05 for t-test comparing to EUC
c. Lifetime utilization at baseline, interval utilization at 3 and 6 months.

© 2025 Pfeiffer PN et al. *JAMA Network Open*.

eTable 3. Missing Values by Study Arm and Engager Status[a]

Outcome Measure	Baseline PREVAIL (N=229)	Baseline EUC (N=226)	3 Months PREVAIL ITT (N=149)	3 Months Engagers (N=108)	3 Months EUC (N=158)	6 Months PREVAIL ITT (N=141)	6 Months Engagers (N=103)	6 Months EUC (N=142)
Suicide Attempt	0	0	7	3	7	10	9	7
Beck Scale for SI	0	0	0	0	0	0	0	0
Beck Scale for SI – Worst Point	--	--	0	0	0	0	0	0
Beck Hopelessness Scale	1	0	11	8	10	6	5	8
INQ Perceived Burdensomeness	1	0	11	8	11	6	5	9
INQ Thwarted Belongingness	1	0	11	8	11	6	5	9
Hope Scale	1	0	11	8	11	6	5	9
NIH Emotional Support	1	0	11	8	11	6	5	9
NIH Instrumental Support	1	0	11	8	11	6	5	9
NIH Friendship	1	0	11	8	11	6	5	9
NIH Loneliness	1	0	11	8	11	6	5	9
NIH Rejection	1	0	11	8	11	6	5	9
Suicide self-efficacy	0	0	12	8	10	8	6	8
Q-LES-Q-SF	1	0	11	8	10	6	5	8
SF-12 Physical health subscale	2	4	13	8	13	7	6	8
SF-12 Mental health subscale	2	4	13	8	13	7	6	8
MDPSS total mean	2	0	11	8	11	6	5	9
MDPSS significant other subscale	2	0	11	8	11	6	5	9
MDPSS family subscale	2	0	11	8	11	6	5	9
MDPSS friend subscale	2	0	11	8	11	6	5	9
Patient Health Questionnaire (PHQ-9)	1	0	11	8	11	6	5	9
MLQ – Presence	1	0	13	9	12	6	5	9
MLQ – Search	1	0	13	9	12	6	5	9
Any Inpatient Psychiatry Admission	2	0	11	8	12	7	6	9
Any Mental Health Service Use	2	0	11	8	12	7	6	9

Abbreviations: ITT = intent to treat sample; EUC = enhanced usual care; SI = Suicidal ideation; INQ = Interpersonal Needs Questionnaire; NIH = National Institutes of Health; Q-LES-Q = Quality of Life Enjoyment and Satisfaction Questionnaire Short Form; SF-12 = 12-item Short Form; MDPSS = Multidimensional Scale of Perceived Social Support; MLQ = Meaning in Life Questionnaire

a. Engagers = intervention participants who completed ≥ 6 sessions with a peer specialist.

© 2025 Pfeiffer PN et al. *JAMA Network Open*.

eMethods. Generalized Linear Mixed-Effects Model Methods and Results

Methods supplement:

To examine suicidal ideation over time, we used a mixed effects model with logit link with any lifetime suicide attempt at baseline and any suicide attempt in the prior 3 months at 3 and 6 months as the dependent variable. To examine suicidal ideation over time, we used a linear mixed-effects model with ideation assessed at baseline, 3- and 6-months as the dependent variable. Mixed-effects models included an intervention indicator, time indicators (for 3- and 6-months), and intervention by time interaction terms. Intervention effects on each outcome at 3 and 6 months were determined according to the respective interaction terms. All mixed-effects models included patients as random intercepts with unstructured correlation structure and were adjusted for study sites, gender, and prior suicide attempt used in stratified randomization and minimization, and for potentially confounding covariates shown to differ between arms at baseline and baseline predictors of missing follow-up. With respect to missing data, linear mixed-effects models are expected to provide an unbiased estimate of the intervention effect assuming missingness is at random conditional on baseline predictors of missing follow-up.

Results supplement:

In the longitudinal data models, the interaction term between PREVAIL arm and time was not significant for suicide attempts or suicidal ideation at either time point (eTable 4).

© 2025 Pfeiffer PN et al. *JAMA Network Open*.

eTable 4. Generalized Linear Mixed-Effects Models of Primary Outcomes Across All Assessment Time Points

	Suicide Attempts over 6 months			Suicide Ideation over 6 months		
	Odds Ratio		P-value	Beta coefficient		P-Value
Parameters	Point Estimate	95% CI		Point Estimate	95% CI	
Intercept	2.72	(2.32, 3.19)	<.001	19.97	(15.86, 24.07)	<0.001
Time at 3 months	0.44	(0.41, 0.47)	<.001	-17.93	(-19.29, -16.57)	<.0001
Time at 6 months	0.45	(0.42, 0.48)	<.001	-18.30	(-19.73, -16.88)	<.0001
PREVAIL Intervention (ref: EUC)	0.94	(0.88, 0.99)	.03	-0.20	(-1.46, 1.07)	.76
PREVAIL X 3 months	1.07	(0.98, 1.18)	.14	0.32	(-1.63, 2.26)	.75
PREVAIL X 6 months	1.02	(0.93, 1.13)	.63	0.74	(-1.22, 2.69)	.46
Recruitment Site A (ref: Site B)	0.99	(0.94, 1.03)	.58	-1.20	(-2.08, -0.32)	<.01
Male (ref: Female)	0.95	(0.89, 1.00)	.06	0.18	(-0.75, 1.10)	.71
Transgender/Non-binary/Gender not listed (ref: Female)	1.00	(0.90, 1.11)	.96	0.58	(-1.07, 2.23)	.49
Unstable housing	1.10	(1.00, 1.21)	.05	-0.15	(-1.93, 1.62)	.87
Hopelessness	1.00	(1.00, 1.01)	.19	0.28	(0.19, 0.36)	<.001
Family support	0.99	(0.97, 1.00)	.18	-0.30	(-0.56, -0.04)	.03
Friend support	1.00	(0.99, 1.01)	.89	0.08	(-0.26, 0.41)	.64
Emotional support	1.00	(1.00, 1.00)	.88	0.04	(-0.05, 0.12)	.41
Instrumental support	1.00	(1.00, 1.00)	.31	-0.03	(-0.11, 0.04)	.34
Perceived rejection	1.00	(1.00, 1.00)	.98	0.05	(-0.03, 0.13)	.18

EUC = enhanced usual care.

© 2025 Pfeiffer PN et al. *JAMA Network Open*.
