# Supplementary material for: Peer Support Intervention for Suicide Prevention Among High-Risk Adults in Michigan: A Randomized Clinical Trial
Source: JAMA Netw Open. 2025 May 28;8(5):e2510808. doi: 10.1001/jamanetworkopen.2025.10808 (PMC12120652; doi:10.1001/jamanetworkopen.2025.10808)
Supplement: Supplement 3. — Data Sharing Statement [file jamanetwopen-e2510808-s003.pdf]

Data Sharing Statement

Pfeiffer. Peer Support Intervention for Suicide Prevention Among High-Risk Adults in Michigan. *JAMA Netw Open*. Published May 28, 2025. doi:10.1001/jamanetworkopen.2025.10808

Data
Additional Information: NCT03373916
Data available: Yes
Data types: Deidentified participant data
How to access data: Deidentified data (only from participants who provided broad consent for future research use; a small number of study participants declined to consent to this) is available through the NIH Data Archive: https://nda.nih.gov/nda/access-data-info
When available: beginning date: 12-31-2023

Supporting Documents
Document types: None

Additional Information
Who can access the data: Data will be made available by request. Instructions for requesting data access are available online here: https://nda.nih.gov/nda/access-data-info.
Types of analyses: Data available in this repository is only from study participants who provided broad consent for unspecified future use of their data, so we have no restrictions on what types of analyses data can be made available for.
Mechanisms of data availability: Data access is managed solely by NIH. Details on how data access is granted are available here: https://nda.nih.gov/nda/access-data-info
